# Mapping Executive Function Performance Based on Resting-State EEG in Healthy Individuals: A Systematic and Mechanistic Review

**DOI:** 10.3390/jcm15031306

**Published:** 2026-02-06

**Authors:** James Chmiel, Donata Kurpas

**Affiliations:** 1 Institute of Neurofeedback and tDCS Poland, ul. 3 Maja 25-27, 70-215 Szczecin, Poland; 2 Division of Research Methodology, Department of Nursing, Faculty of Nursing and Midwifery, Wrocław Medical University, 51-618 Wrocław, Poland

**Keywords:** EEG, electroencephalography, electroencephalogram, executive functions, working memory, decision making, inhibitory control, brain oscillations, neurophysiology

## Abstract

**Introduction**: Resting-state EEG (rsEEG) is a scalable window onto trait-like “executive readiness,” but findings have been fragmented by task impurity on the executive-function (EF) side and heterogeneous EEG pipelines. This review synthesizes rsEEG features that reliably track EF in healthy samples across development and aging and evaluates moderators such as cognitive reserve. Materials and methods: Following PRISMA 2020, we defined PECOS-based eligibility (human participants; eyes-closed/eyes-open rsEEG; spectral, aperiodic, connectivity, topology, microstate, and LRTC features; behavioral EF outcomes) and searched MEDLINE/PubMed, Embase, PsycINFO, Web of Science, Scopus, and IEEE Xplore from inception to 30 August 2025. Two reviewers were screened/double-extracted; the risk of bias in non-randomized studies was assessed using the ROBINS-I tool. Sixty-three studies met criteria (plus citation tracking), spanning from childhood to old age. **Results**: Across domains, tempo, noise, and wiring jointly explained EF differences. Faster individual/peak alpha frequency (IAF/PAF) related most consistently to manipulation-heavy working may and interference control/vigilance in aging; alpha power was less informative once periodic and aperiodic components were separated. Aperiodic 1/f parameters (slope/offset) indexed domain-general efficiency (processing speed, executive composites) with education-dependent sign flips in later life. Connectivity/topology outperformed local power: efficient, small-world-like alpha networks predicted faster, more consistent decisions and higher WM accuracy, whereas globally heightened alpha/gamma synchrony—and rigid high-beta organization—were behaviorally sluggish. Within-frontal beta/gamma coherence supported span maintenance/sequencing, but excessive fronto-posterior theta coherence selectively undermined WM manipulation/updating. A higher frontal theta/beta ratio forecasts riskier, less adaptive choices and poorer reversal learning for decision policy. Age and reserve consistently moderated effects (e.g., child frontal theta supportive for WM; older-adult slow power often detrimental; stronger EO ↔ EC connectivity modulation and faster alpha with higher reserve). Boundary conditions were common: low-load tasks and homogeneous young samples usually yielded nulls. **Conclusions**: RsEEG does not diagnose EF independently; single-band metrics or simple ratios lack specificity and can be confounded by age/reserve. Instead, a multi-feature signature—faster alpha pace, steeper 1/f slope with appropriate offset, efficient/flexible alpha-band topology with limited global over-synchrony (especially avoiding long-range theta lock), and supportive within-frontal fast-band coherence—best captures individual differences in executive speed, interference control, stability, and WM manipulation. For reproducible applications, recordings should include ≥5–6 min eyes-closed (plus eyes-open), ≥32 channels, vigilant artifact/drowsiness control, periodic–aperiodic decomposition, lag-insensitive connectivity, and graph metrics; analyses must separate speed from accuracy and distinguish WM maintenance vs. manipulation. Clinical translation should prioritize stratification and monitoring (not diagnosis), interpreted through the lenses of development, aging, and cognitive reserve.

## 1. Introduction

Executive functions (EFs) are domain-general control processes that coordinate perception, memory, and action to keep behavior aligned with current goals, especially in the face of novelty, conflict, or distraction. Decades of individual-differences research show that EFs display “unity and diversity”: partially separable components—updating (working memory), inhibitory control, and set-shifting (cognitive flexibility)—that nonetheless share a common EF factor capturing goal maintenance and interference control. Working memory denotes the short-term maintenance and manipulation of information (verbal and visuospatial) under the guidance of a “central executive.” A key update to the classic model is the episodic buffer, a limited-capacity store that binds information across modalities and to long-term memory, supporting integrated, consciously accessible representations [1]. Attention provides the selection and prioritization that EF relies on; influential accounts distinguish alerting, orienting, and executive attention networks, while dorsal and ventral attention systems coordinate top-down selection and stimulus-driven reorienting [2,3]. Decision making, in neuroeconomic terms, entails constructing and comparing subjective values for candidate actions; converging work implicates ventromedial/orbitofrontal prefrontal cortex (vmPFC/OFC) and ventral striatum in value representation, with lateral prefrontal regions exerting control over choice [4,5]. Inhibitory control is the ability to suppress prepotent or ongoing responses; right inferior frontal gyrus (rIFG), pre-SMA/ACC, and fronto-striatal loops form a core stopping circuit [6,7]. Cognitive flexibility (set-shifting) is the rapid reconfiguration of task sets or stimulus–response mappings in response to changing rules and contingencies; like other EF components, it recruits lateral prefrontal and parietal nodes within a shared control architecture [8].

Measurement leverages experimental paradigms and standardized batteries while grappling with the task-impurity problem (each task mixes executive with non-executive demands). For working memory, n-back tasks parametrize load and reliably tax maintenance/updating; NIH Toolbox List Sorting provides a standardized alternative [9,10]. Executive attention is commonly assessed with flanker and Attention Network Test variants that parse alerting, orienting, and conflict control [11]. Inhibitory control is measured with the Stroop (interference control), the Go/No-Go task (withholding prepotent responses), and the stop-signal task (SSRT indexing stopping latency); methodological consensus papers now specify best practices for stop-signal estimation [12,13]. Cognitive flexibility is captured by task-switching and the Dimensional Change Card Sort (NIH Toolbox) [14]. Decision-making measures include the Iowa Gambling Task (experience-based, affect-laden valuation) [15]. Because many classic tasks show robust experimental effects but weak test–retest reliability for individual-differences work, current recommendations emphasize multi-task batteries, latent-variable modeling, and careful reliability checks when linking EF to brain measures [16].

Executive functions change markedly across the lifespan, following a broadly nonlinear pattern from emergence and rapid growth in early childhood to protracted refinement through adolescence, relative stability in early and middle adulthood, and heterogeneous decline in later life. In preschool years, elementary forms of working memory, inhibitory control, and cognitive flexibility are already measurable and improve quickly as children learn to hold rules in mind, resist prepotent responses, and shift between task sets; these gains reflect both behavioral development and early specialization of control processes [17]. By early adulthood, many executive abilities reach a peak. Still, they do not peak at a single age: different components crest at other times (e.g., processing speed earlier than working memory or higher-order reasoning), and some skills show modest improvements into the fourth decade before stabilizing [18]. Large cross-sectional batteries in middle-aged individuals confirm protracted development into the 20s–30s, followed by domain-specific plateaus and subsequent declines, underscoring unity and diversity in executive trajectories [19]. Beginning in later midlife and accelerating in older age, many people experience declines in processing speed, interference control, task switching, and working memory updating; classic models attribute a sizeable share of age differences in “fluid” cognition to slowing, which reduces the time available for controlled operations and undermines the simultaneous maintenance of intermediate results [20,21].

Resting-state EEG (rsEEG) measures the brain’s spontaneous electrical activity. At the same time, a person is awake but not engaged in an explicit task, most often in standardized “eyes-closed” (EC) and “eyes-open” (EO) conditions. In routine practice and research, rsEEG captures ongoing cortical population dynamics with millisecond precision from electrodes placed on the scalp (e.g., the international 10–20/10–10 layouts) [22,23], yielding a time series that is rich enough to quantify rhythmic (oscillatory) and non-rhythmic (aperiodic) features, spatial topographies, and network organization [24]. EC and EO are not interchangeable: EO reduces posterior alpha and increases higher-frequency power (“alpha reactivity”), whereas EC amplifies the classic posterior alpha idling rhythm; alternating EC/EO blocks are therefore a simple probe of arousal and reactivity that can be embedded in a resting protocol [25]. Standard technical guidance covers montage, sampling, impedance, filtering, vigilance control, and minimum duration to obtain stable estimates [26].

At the most basic analytic level, rsEEG is transformed into the frequency domain to estimate the power spectral density (PSD). Canonical bands—delta (~0.5–4 Hz), theta (~4–8 Hz), alpha (~8–13 Hz), beta (~13–30 Hz), gamma (>30 Hz)—provide a convenient shorthand for predominant timescales of neural population activity [27]. Two complementary measures are commonly reported: absolute power (µV^2^/Hz) and relative power (the proportion of total power within a band) [28]. “Neurometrics” compares an individual’s band-limited values and spatial patterns to age-stratified normative databases, often by Z-scoring across electrodes and bands; this ages the signal (literally), because power, peak frequencies, and topographies change systematically over the lifespan [29,30,31,32,33,34]. These practices trace back to quantitative EEG frameworks that emphasize spectral analysis, topographic maps, and statistical methods for clinical and cognitive inference [30].

Beyond raw power, two spectral features have become especially informative in rest: the individual alpha peak frequency (IAF) and the aperiodic “1/f-like” background. IAF is the frequency at which alpha power peaks; it varies across people, declines with age, and shows trait-like stability across sessions [35,36]. The aperiodic component reflects broadband scale-free activity usually summarized by an exponent (spectral slope) and an offset (broadband level) [37,38]. Parameterizing spectra into periodic (true oscillations) and aperiodic parts prevents confounds (e.g., “alpha power” inflated by slope) and yields interpretable physiology [37,38]: steeper slopes and higher offsets generally indicate less neural “noise” [39], different excitation/inhibition balance [40], and higher population firing rates at rest [41]. With aging, exponents tend to flatten (more 1/f “noise”), and offsets often decrease [29]; both metrics relate to individual differences in processing speed [42], arousal [43], and vigilance [44] beyond classic band power.

Band ratios—compact summaries of relative slow- versus fast-wave activity—are another “form” of rsEEG widely used in cognitive and affective work. The frontal theta/beta ratio (TBR) indexes the balance between slow control/monitoring rhythms and faster sensorimotor/attention rhythms [45,46]; higher TBR at rest has been associated, for example, with more mind-wandering [47]. A related cross-frequency measure is delta–beta coupling (e.g., power–power correlations or phase–amplitude indices across time), often interpreted as an index of cortical–subcortical regulation and stress reactivity: more substantial frontal delta–beta coupling has been observed in more anxiogenic states [48,49]. These ratio/coupling metrics are sensitive but mechanistically heterogeneous and should be interpreted alongside absolute/relative power and aperiodic parameters.

Resting-state connectivity is a primary form: it asks how oscillations co-vary across regions. Because raw coherence is inflated by volume conduction and standard reference, modern practice favors measures that suppress zero-lag coupling—e.g., the imaginary part of coherence and the phase-lag index (PLI)—or moves into source space. It computes lagged metrics after electrical source imaging (e.g., sLORETA’s lagged connectivity) [50,51,52]. These pairwise couplings can then be embedded in graph-theoretic analyses (clustering coefficient, characteristic path length, small-worldness, global/local efficiency) to quantify segregation/integration of resting networks [53,54]. Healthy rsEEG typically exhibits small-world structure [55,56,57]; meaningful individual differences in fronto-occipital alpha connectivity at rest are associated with learning performance [58]. However, connectivity is exquisitely sensitive to referencing, filtering, and artifact handling, so conservative pipelines and replication are essential [59,60,61,62].

Another distinctive “form” is the microstate: the EEG scalp map remains quasi-stable for ~60–120 ms before abruptly switching to a new template [63]. Clustering the entire resting record yields a small set (typically four to five) of canonical classes (A–D or A–E) whose temporal properties—mean duration, occurrence rate, time coverage, global explained variance (GEV), and transition probabilities—summarize brain-wide dynamics at the sub-second scale [63]. Microstates show robust EC/EO modulations [64], trait reliability [64], and partial correspondence with large-scale functional systems (e.g., classes often linked to auditory, visual, and dorsal attention networks) [65]. Aging and arousal affect their dwell times and transitions [66,67].

The temporal structure itself can be a target: rsEEG amplitude fluctuations exhibit long-range temporal correlations (LRTCs) that are well captured by detrended fluctuation analysis (DFA) and Hurst exponents [68,69]. LRTCs quantify how present activity depends on its distant past—an index of multiscale stability versus flexibility [69,70]. Such scale-free dynamics vary across bands (often strongest in alpha) and individuals, with links reported to vigilance regulation [71,72].

Resting-state EEG offers a low-cost, scalable window onto trait-like neural dynamics—oscillatory power and peak frequencies, aperiodic 1/f parameters, band ratios, connectivity, microstates, and long-range temporal structure—that plausibly constrain the efficiency of executive control. However, the literature is still fragmented by task impurity on the EF side and heterogeneous acquisition and analysis choices on the EEG side (eyes-open vs. eyes-closed, referencing, spectral parameterization, sensor vs. source space). Clarifying whether any rsEEG features robustly track individual differences in EF in healthy populations is theoretically informative—linking large-scale intrinsic dynamics to the unity-and-diversity architecture of EF—and practically valuable for establishing normative benchmarks and guiding future longitudinal and interventional work. This systematic review will aim to synthesize the results of various types of resting-state EEG studies that have correlated EEG findings with executive function performance. We will address the questions of whether there are consistent biomarkers of EEG activity responsible for executive function performance, how differences in executive function performance in healthy individuals of different age groups differ in terms of EEG activity, and how parameters such as developmental stage (childhood, adulthood, or old age) or cognitive reserve influence executive function and EEG activity. Furthermore, the review will elucidate the EEG-based neural mechanisms responsible for executive function performance.

## 2. Materials and Methods

### 2.1. Protocol and Reporting

This review was designed and reported per the PRISMA 2020 statement and its Explanation/Elaboration guidance. A protocol specifying the research question, eligibility criteria, outcomes, and analysis plan was developed a priori. The protocol was registered in PROSPERO (CRD420251165555). See Supplementary Material File S1 for the checklist [73].

### 2.2. Eligibility Criteria

We defined eligibility using PECOS (Participants–Exposure–Comparator–Outcomes–Study design):

1. Participants. Human participants of any age (children, adolescents, adults, older adults), including healthy, community-dwelling samples and non-demented at-risk groups (e.g., subjective decline, MCI). Animal studies were excluded.
2. Exposure. Resting-state EEG recorded during eyes-closed and/or eyes-open conditions with no active task demands. Eligible EEG features included (but were not limited to) band-limited spectral power (delta, theta, alpha, beta, gamma; absolute/relative), individual/peak alpha frequency (IAF), aperiodic (“1/f”) parameters (exponent/slope and offset), band ratios (e.g., theta/beta, delta/beta), microstates (A–E and derivatives), connectivity/coherence/coupling indices (bivariate and multivariate; lagged/PLI/imaginary coherence), graph-theoretic/network metrics (e.g., clustering, characteristic path length, efficiency), and long-range temporal correlations (e.g., DFA/Hurst). Studies using only task-evoked EEG/ERPs without a resting acquisition were excluded.
3. Comparator. Not required. Studies could be single-group correlational analyses or between-group comparisons. When comparators were used (e.g., healthy vs. MCI), both arms had to include resting EEG and cognitive outcomes.
4. Outcomes. Behavioral measures of cognition administered independently of the resting recording, including working memory (e.g., digit span, n-back, Sternberg), inhibitory control/executive attention (e.g., Stroop, flanker, antisaccade, stop-signal), selective and sustained attention (e.g., SART, RVP, CPT, visual search), decision making (e.g., IGT, reversal learning), processing speed, reasoning, verbal fluency, and global cognition. Studies reporting diffusion-model parameters (e.g., drift rate) were eligible. Neurophysiological or imaging outcomes without behavioral cognition were excluded.
5. Study designs. Observational cross-sectional or longitudinal cohorts, secondary analyses of open datasets, and experimental studies that correlated baseline (pre-task) resting EEG with later task performance. Case reports, reviews, tutorials, editorials, conference abstracts without full text, and studies without sufficient statistical information were excluded unless the authors could obtain data.
6. Setting, time, language. Any setting, database inception to 30 August 2025. Only studies in English were eligible.

### 2.3. Information Sources

We searched the following bibliographic databases from inception to 30 August 2025: MEDLINE/PubMed, Embase, PsycINFO, Web of Science Core Collection, Scopus, and IEEE Xplore (for engineering/EEG methods papers with cognitive outcomes). We also hand-searched reference lists of included studies and relevant reviews and tracked forward citations in Web of Science and Google Scholar.

### 2.4. Search Strategy

The PubMed strategy (adapted per database syntax) combined EEG terms with resting-state and cognitive outcomes: “electroencephalography” OR “EEG” OR “qEEG” OR “resting-state EEG” OR “rest EEG” AND “working memory” OR “digit span” OR “n-back” OR “Sternberg” OR “attention” OR “sustained attention” OR “vigilance” OR “selective attention” OR “inhibition” OR “inhibitory” OR “Stroop” OR “flanker” OR “antisaccade” OR “stop-signal” OR “decision making” OR “Iowa Gambling” OR “reversal learning” OR “processing speed” OR “reasoning” OR “verbal fluency” OR “executive function” OR “cognitive function” OR “cognition”. Additionally, studies initially matching the scope of the review were searched for cited studies to identify similar studies that might fit the scope of the review. Furthermore, PubMed algorithms were used to suggest similar and cited articles.

### 2.5. Study Selection

**Figure 1:**
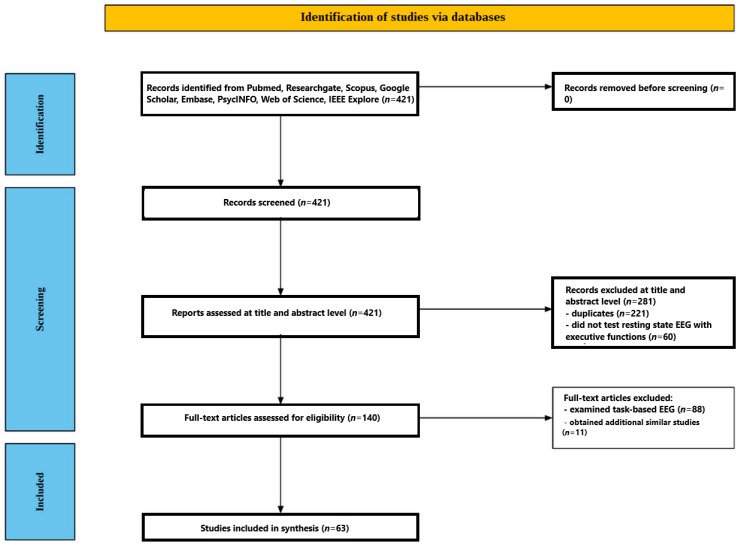
Flowchart depicting the phases of the systematic review.

Records were deduplicated (EndNote X9) and independently screened by two reviewers at the title/abstract and full-text levels against the prespecified criteria. Disagreements were resolved by consensus or a third reviewer. Reasons for full-text exclusion were documented. The selection process is summarized in a PRISMA flow diagram (Figure 1).

### 2.6. Data Collection Process

Two independent reviewers used a standardized extraction form (pilot-tested on a 10-study sample). Discrepancies were reconciled by discussion. When necessary, data were missing or ambiguous, and study authors were contacted up to 2 times.

### 2.7. Data Items

We extracted the following:

1. Study descriptors: Year, country, design (cross-sectional/longitudinal/experimental with baseline EEG), recruitment setting, inclusion/exclusion criteria.
2. Sample: N per group, age (mean/SD or range), sex distribution, handedness (if available), clinical status (healthy, subjective decline, MCI, AD), education/cognitive reserve proxies when reported.
3. EEG acquisition: System and montage (number of channels, 10–20 positions), sampling rate, reference, recording condition (eyes closed/open; duration), preprocessing (filters, artifact handling/ICA, bad channel criteria).
4. EEG features: Spectral power (absolute/relative; delta, theta, alpha sub-bands, beta sub-bands, gamma); peak alpha/IAF/PAF (site/derivation); aperiodic exponent/offset and decomposition method; ratios (theta/beta, delta/beta, alpha/theta, etc.); coherence/PLI/lagged connectivity (pairs/ROIs); graph metrics (clustering, characteristic path length, global/local efficiency, small-worldness); microstates (A–E; duration, occurrence, coverage, GEV; transitions); DFA/Hurst estimates; hemispheric asymmetries; eyes-open vs. eyes-closed contrasts.
5. Cognitive outcomes: Task names and versions; outcome metrics (accuracy, reaction time, drift rate, SSRT, error rates, composite scores); timing of testing relative to resting EEG; test–retest intervals for longitudinal studies.
6. Statistics: Effect sizes linking resting features to outcomes (correlations r or ρ, regression coefficients β/standardized b, group contrasts), covariates used (e.g., age, sex, education), multiple comparison adjustments, and whether results were preregistered.

### 2.8. Risk of Bias in Individual Studies

Since a significant portion of the analyzed studies were non-randomized, the ROBINS-I tool was used to assess the risk of bias.

## 3. Results

The search across various databases yielded 421 studies tentatively matching the criteria. Following deduplication, 200 records were identified. A total of 60 studies were excluded because they did not assess resting-state EEG in conjunction with executive functions. Based on title and abstract screening, 140 full-text articles were reviewed. Of these, 88 studies were excluded because they examined task-based EEG. After full-text screening, 52 studies met the inclusion criteria. An additional 11 studies were identified through citation tracking of included articles, bringing the total to 63 [74,75,76,77,78,79,80,81,82,83,84,85,86,87,88,89,90,91,92,93,94,95,96,97,98,99,100,101,102,103,104,105,106,107,108,109,110,111,112,113,114,115,116,117,118,119,120,121,122,123,124,125,126,127,128,129,130,131,132,133,134,135,136]. The included studies are presented in Table 1. The risk of bias is presented in Table 2.

### 3.1. Participant Characteristics

Across the included literature, samples were overwhelmingly neurologically healthy and community-dwelling, with explicit screening to exclude psychiatric or primary medical illness, substance use, and psychotropic medications; when stated, handedness was often restricted to right-handed participants [74,75,76,78,80,84,98,99,100,101,102,104,105,107,108,113,123,126,127,129]. Cognitive status in older cohorts was commonly verified with MMSE or MoCA (e.g., MMSE ≥ 25; MoCA for global cognition) [75,93,98,113].

Age. Participant ages spanned from early childhood to late old age. Many studies targeted older adults (≈60–80+ years), including cohorts with subjective cognitive complaints and cognitively normal aging; several included MCI or AD comparison groups [74,75,91,93,98,99,100,105,106,107,108]. Numerous studies examined young adults (late teens to early 30s) and midlife samples [78,80,81,82,83,84,85,89,90,101,102,117,118,124,125,126,127,128,129,130,131,132,133,134,135,136]. Developmental work covered children/adolescents [109,110,111,112].

Sample size. Cohorts ranged from tiny laboratory samples (n ≈ 10–30, e.g., [8,84,85,123]) through moderate samples (n ≈ 40–170, e.g., [90,94,95,113,117,118]) to large-scale datasets ([78] n = 550; [105] n = 287 overall with EEG subgroup; [108] n = 1703; [129] n = 235).

Sex distribution. Sex ratios varied: several convenience samples were female-skewed [75,90,112], others were balanced by design [78,101,106], and a few were single-sex for control purposes (all men: [85,130]; all women: [133,135]).

Health, cognition, and special subgroups. Beyond healthy volunteers, samples included at-risk aging (older adults with subjective decline but no dementia, MCI, or AD comparators) ([75,93,98,99]); sociodemographic contrasts via cognitive reserve (education/IQ/occupation), social vulnerability/SES, and incidental physical activity [87,90,92,94,113,125]; and developmental cohorts of typically developing children [109,110,111,112].

Education and cognitive reserve. Years of education and cognitive reserve proxies were frequently recorded and sometimes used for grouping or as covariates [87,90,94,105,113,129]. Undergraduate and university-based samples generally had high schooling [80,101,102,116,117,118,132,133,134,135,136].

### 3.2. EEG Characteristics

Recording conditions and montages. Nearly all studies used resting-state EEG with eyes closed, often alternating with eyes open blocks; several paired rest with brief cognitive tasks for reactivity analyses [74,76,78,81,84,89,93,98,100,101,106,107,108,115,122,126,127]. Montages ranged from low-density (19–32 electrodes) to high-density (60–128+): 19-channel clinical caps [74,92,97,128]; 31–32 channels [88,93,111,119,120]; 60–64 channels [80,82,83,84,115,117,118,121,122,123]; and 128 channels [129]; consumer-grade 14-channel headsets were also used [107]. Some older work analyzed temporo-occipital derivations specifically [91], while several studies summarized by lobar ROIs (frontal/central/parietal/temporal/occipital) [76,107,121].

Duration and structure. Rest intervals spanned 1–20 min, delivered in single blocks or EO/EC alternations (e.g., 1–2.5 min blocks, 4–7 min total, or 16–20 min protocols) [76,78,81,84,93,101,107,108,115,122]. Segment length choices for spectral estimates varied (e.g., 2 s vs. 8 s windows to trade sample count vs. low-frequency resolution) [93].

Spectral bands and power metrics. Power was quantified for canonical bands with study-specific cutoffs: delta (~0.1–4 Hz), theta (4–8 Hz), alpha (often split into α1/α2), beta (~12–30 Hz), and gamma (~30–50 Hz) [74,75,76,77,84,93,94,95,107,126]. Both absolute and relative power were used; some were standardized to age-normed Z scores ([74]). Peak/individual alpha frequency (PAF/IAF) and alpha power were frequent endpoints in lifespan and executive-control work [78,100,102,105,108,114,129].

Aperiodic (1/f) measures. Multiple studies decomposed spectra into periodic vs. aperiodic components, extracting exponent (slope) and offset as indices of excitation–inhibition balance or broadband activity [80,87,100,104,124,129].

Ratios and asymmetries. Widely used composite markers included theta/beta (often frontal), delta/beta, theta/alpha, beta/alpha, and theta–alpha ratio (TAR); several papers emphasized hemispheric asymmetry (e.g., prefrontal β/α) [89,97,111,116,128,132,133,134,135,136]. Alpha reactivity (decrease from EC → EO or activation) was also analyzed [98].

Connectivity and network topology. Beyond power, many studies quantified functional coupling via coherence (local and long-range), phase synchrony/PLI, and connectivity-based graph metrics (clustering, path length, efficiency), including lagged linear connectivity to mitigate volume conduction [81,90,94,95,107,122,123,125]. Several works modeled time-varying alpha-band networks and entropy/variability indices at rest [121,122].

Microstates. High-density eyes-closed EEG was segmented into canonical microstates (A–E) with metrics such as duration, occurrence, coverage, global explained variance, and transition probabilities [79,115,117,118].

Advanced temporal structure. Some studies characterized long-range temporal correlations using detrended fluctuation analysis (DFA) within bands (yielding scaling/Hurst exponents) [96].

Preprocessing and QC. Pipelines typically included filtering, ocular/muscle artifact correction, and stationarity checks; examples include artifact subspace reconstruction on portable systems and explicit verification against drowsiness drift across recording halves [93,107]. Feature extraction sometimes uses PCA (frequency/temporal) to derive latent spectral or ERP components aligned to task epochs in rest–task designs [126,127].

Region of interest focus. Analyses frequently emphasized frontal (including midline) leads for executive-related theta/beta, with complementary parietal/occipital alpha and motor/IFC beta in inhibition-oriented work; some targeted frontal–posterior coupling explicitly [76,81,95,107,116,131,134].

### 3.3. Resting-State EEG Correlates of Working Memory

#### 3.3.1. Big Picture

Across the studies, resting-state EEG relates to WM in a pattern that is consistent when accounting for age, topography, and task demands: the clearest positive marker is a faster alpha rhythm, especially frontally—higher individual/peak alpha frequency predicts stronger manipulation-heavy WM (e.g., reverse Digit Span) over and above age, sex, and education, and even fluctuates with same-day WM “readiness” (PAF rises when underprepared individuals engage the task) [78,114]. In healthy older adults, less slow-wave activity at rest—lower delta/theta, particularly frontally—tends to accompany better WM in some studies [74] and is also seen in more active or high-reserve groups that show WM-adjacent/executive advantages and ‘younger’ EEG profiles, though not necessarily direct WM gains [92,113]; however, when WM is indexed by speeded match-to-sample responding, greater left-parietal delta (and marginal theta) can predict faster responses while higher right-parietal alpha/beta relates to poorer accuracy, underscoring that power–performance links can flip with region and whether the outcome is speed or accuracy [107]. Connectivity quality outperforms raw power: higher frontal coherence (beta/gamma) tracks better Digit Span Forward/Sequencing, whereas excessive fronto-posterior theta coherence predicts worse Sequencing (i.e., manipulation/updating), suggesting that over-synchronization in long-range theta can be maladaptive for ordered WM [95]. Measures of temporal organization and network flexibility converge on the same story: lower long-range temporal correlation exponents in slow bands (delta/theta) and more variable/efficient alpha-band networks at rest are associated with higher WM accuracy and stronger fronto-parietal integration that can reconfigure under load [96,121]. Development changes the sign of some associations: in children, greater frontal theta at rest relates positively to WM (Digit Span), whereas in aging, the advantageous profile typically involves reduced slow-band power and faster alpha [109]. Finally, task choice and sample composition matter: low-load or one-back paradigms often yield nulls even with careful EEG (e.g., [75,111], and large young-adult samples frequently show no reliable WM links for band ratios or microstate metrics [115,117,128], while huge aging cohorts sometimes find alpha/aperiodic markers map more robustly onto executive speed/control than onto memory per se) [108,129].

#### 3.3.2. Spectral Power (Delta/Theta/Alpha/Beta)

Across studies, delta/theta power at rest shows an age- and topology-dependent relationship to WM. In healthy older adults, lower slow-band power—especially frontal theta/delta—tracks better WM or WM-adjacent performance: WAIS-III Working Memory Index is higher when frontal theta (and delta) are lower [74]; MCI vs. healthy contrasts show elevated posterior theta in MCI and, within healthy elders, higher global theta relates to lower MoCA, consistent with a “more slow power → worse cognition” pattern [93]; high-reserve and more active lifestyle groups likewise exhibit reduced theta/delta together with better processing-speed/executive profiles and younger-looking EEG, but these studies did not demonstrate direct WM gains [92,113]. Critical counterpoints indicate regional/task contingencies: in an older sample performing a delayed match-to-sample WM task, higher left-parietal delta (and marginal theta) predicted faster reaction times (speed benefit), whereas accuracy related oppositely to higher right-parietal fast power (see below), underscoring that slow power can aid speed while not guaranteeing accuracy [107]. Links can differ by cohort: in an older-adult sample (56–70 y), frontal/parietal theta correlated positively with several cognitive measures and beta at Pz correlated negatively with WM span, implying that some theta may support executive/WM processes in elderly adults [76]; in children, stronger frontal theta at rest robustly associates with better Digit Span WM, and multivariate EEG patterns emphasize theta as the band most tied to income-linked WM differences [109,112]. Nevertheless, when WM load is low (e.g., one-back), slow-band power often fails to correlate with performance in healthy adults and school-age samples [75,111]. For alpha power, effects are region- and outcome-specific rather than uniformly beneficial: classic findings in elders link higher alpha power to speed/perceptual organization rather than WM per se [74], yet in the WM match-to-sample study higher right-parietal alpha related to lower accuracy, suggesting that greater posterior idling may hinder precise target–distractor discrimination in WM retrieval [107]; large aging cohorts also show that right-frontal alpha power relates to slower processing speed (again not WM-specific), and memory factors can be alpha-insensitive once broader models are considered [108]. (By contrast, alpha frequency—not power—shows clearer WM associations, but that is outside pure power metrics.) Finally, beta power provides the least consistent WM signal across studies: in elders, its correlations are sparse or unsystematic [74]; in the older WM study, higher right-parietal beta predicted lower WM accuracy [107]; in mid-life adults, beta at Pz showed a negative link with WM span [76]; and in other cohorts, beta power more often tracks attentional speed/consistency rather than WM capacity per se [85]. Band ratios echo these nuances: in young adults, a higher theta/alpha ratio is related to better short-term memory, whereas in older adults, the relation did not hold, and reasoning showed the opposite pattern (higher TAR → worse performance) [97]. Net result is that for WM, less frontal slow power tends to be advantageous in aging, some frontal theta is supportive in development/younger adults, alpha power effects hinge on topography (posterior increases may impede WM accuracy), and beta power shows mainly weak or negative WM associations that are task- and site-dependent. In the study [88], the results showed apparent age-related differences in WM during the Sternberg task. Behaviorally, both younger and older participants performed well overall, with high recognition accuracy in both groups. Accuracy was higher for short lists than long lists, as expected, but there were no significant differences between age groups. This finding reinforced that the task was manageable for both groups and that any neural discrepancies could not be easily attributed to significant performance disparities. When participants were resting with their eyes open, theta power was higher overall in the younger group (mean = 3.62 μV^2^) than in the older group (mean = 2.78 μV^2^). Although this difference approached significance, it did not reach the conventional threshold (F (1, 26) = 3.73, *p* = 0.06). Nevertheless, apparent effects of electrode location were found, with both groups showing greater theta power at frontal and central sites than at parietal and temporal regions. Notably, a significant interaction between electrode site and age emerged (F (17, 442) = 2.83, *p* = 0.00, ε = 0.50), and follow-up tests demonstrated that younger adults exhibited significantly higher resting theta power than older adults at specific sites, namely FCz, Cz, and C4. Thus, while resting theta reductions in older participants were measurable, they were relatively modest and spatially restricted compared to the widespread reductions seen during task performance.

#### 3.3.3. Aperiodic (“1/f”) Markers

Aperiodic (1/f-like) features of resting EEG—typically summarized by the exponent (spectral slope; higher = steeper, less “neural noise”) and the offset (broadband power level)—show broad, mostly executive-weighted links to cognition, with selective evidence that touches WM and clear age/education moderators. In a large young–adult sample, the aperiodic slope was a stable, scalp-wide trait component (~60% shared variance across electrodes) that did not map cleanly onto isolated WAIS-IV domains (including WM) but did track higher general intelligence (g) and faster choice reaction times, arguing for a domain-general efficiency signal rather than a WM-specific one [80]. Aging studies refine this picture. When periodic and aperiodic components were disentangled, older adults showed flatter slopes and lower offsets than young adults, and while periodic alpha measures linked to speed and interference control, episodic memory and memory composites were often insensitive to aperiodic parameters in extensive samples, underscoring limited direct ties to memory per se ([108], see also null aperiodic–attention links in [100]). By contrast, two lifespan/age-heterogeneous analyses connect aperiodic structure to executive/WM composites: across 17–71 years, higher exponents and offsets associated with better verbal fluency and with composite scores spanning executive function, WM, and psychomotor speed after controlling for age, with effects distributed over frontoparietal, central, and occipital clusters [104]; and in mid-to-late adulthood, higher exponents related to better baseline executive performance, whereas lower individual alpha peak frequency (IAPF) forecast greater 10-year decline, and critically, a mismatch between exponent and IAPF (e.g., high exponent with slow alpha, or vice versa) predicted the steepest long-term cognitive losses, most substantial within executive functioning that includes WM span [129]. Education modifies these associations in late life: among older adults with lower education, steeper slopes and higher offsets predicted better visual attention and higher working-memory accuracy, whereas in highly educated elders the exponent–performance relation reversed (higher exponents → worse speed/WM), implying that reserve proxies reshape how background signal structure supports control/memory operations [87]. Findings from cognitive-control paradigms further contextualize aperiodic markers: older vs. younger adults differ in 1/f offset alongside alpha power, and offset shows a positive, albeit weaker/exploratory, relation to proactive control rather than WM per se [101]; in young adults, the aperiodic exponent behaves as a state variable—rising from pre-trial to within-trial and more so under No-Go demands—with individuals who have noisier resting brains (flatter slopes) showing larger task-driven noise reduction, highlighting that exponent indexes a stability–flexibility trade-off relevant to control that can indirectly bound WM performance under interference [124]. Finally, strong nulls caution against overgeneralization: in well-powered young-adult samples, neither aperiodic offset nor related resting-state measures reliably predict interference resolution or broad executive composites when tasks are less WM-loaded or when cohorts are homogeneous [102,128]. Together, the most defensible reading is that resting aperiodic structure is a global efficiency/noise marker whose clearest cognitive correlates sit in executive control and speed; direct WM links emerge when WM is embedded in those constructs, are strongest in aging (and moderated by education/reserve), and become predictive over time when considered jointly with rhythmic pace markers like IAPF.

#### 3.3.4. Connectivity, Coherence, and Network Dynamics

Resting-state coupling measures converge on the fact that where and how networks synchronize matter more for WM than raw power. At the sensor level, coherence analyses in healthy older adults show a double dissociation by band and axis: stronger within-frontal coherence is beneficial—frontal beta coherence predicts higher Digit Span Forward and Sequencing, and frontal gamma coherence also relates positively to Digit Span Forward—whereas excessive fronto-posterior theta coherence is detrimental, predicting worse Digit Span Sequencing (i.e., poorer manipulation/updating) [95]. Region- and band-specificity appears again in an eyes-closed, delayed match-to-sample WM study in older adults: greater alpha coherence between right parietal and left frontal sites tracked slower reaction times (cost to speed), while stronger delta/theta coherence among frontal–temporal pairs related to higher accuracy (benefit to precision), underscoring that low-frequency long-range communication can support correctness even as specific alpha couplings slow responding [107]. In midlife samples, connectivity–WM links also depend on the network and frequency: higher cognitive reserve (education/IQ/occupation) associates with stronger low-alpha long-range lagged linear connectivity from occipital to distributed cortex at rest and better spatial WM strategies, while higher social reserve relates to stronger local and long-range theta/low-alpha connectivity (especially left-lateralized) together with fewer spatial WM errors; eyes-open vs. eyes-closed modulation is larger in high-reserve individuals, suggesting greater state gating of network communication that co-travels with WM advantages [90,94].

Beyond static coupling, network topology and dynamics at rest are informative for WM. Graph-theoretic analyses of resting alpha networks show that individuals with greater temporal variability/flexibility (higher fuzzy-entropy of edge time series) and more efficient topology (shorter characteristic path length, higher global/local efficiency, appropriate clustering) achieve higher visual WM accuracy; high-accuracy participants also exhibit stronger, flexible long-range fronto-parietal/occipital connections that scale with memory load, indicating a readiness to reconfigure toward task-relevant integration [121]. Temporal organization of the signal itself carries parallel information: lower long-range temporal correlation exponents (DFA/LRTC) in delta/theta—i.e., less strongly correlated slow fluctuations—associate with better WM, with effects most substantial over posterior scalp and independent of power, implying that “how noisy vs. persistent” slow activity is over time constrains WM capacity [96]. Complementary evidence from general reaction-time paradigms (not WM-specific) shows that a more globally efficient small-world organization during rest predicts faster responses. In contrast, globally stronger alpha/gamma synchronization and networks with greater clustering/longer path lengths predict slower responses, reinforcing that over-synchronized or locally clustered topologies can impair speeded performance. In contrast, integration-optimized networks facilitate rapid processing—constraints that plausibly bound WM speed–accuracy trade-offs [123].

Claims that microstate architecture at rest is a proxy for WM receive mixed support. In aging, older adults show reduced global explained variance and lower occurrence of microstates C and C′ (and D), alongside poorer allocentric spatial WM and dampened transitions into these control- and attention-related maps, linking microstate engagement to spatial memory performance across the lifespan [79]. However, in large healthy young-adult cohorts, microstate A–E parameters (duration, occurrence, coverage, and transitions) show no robust associations with WM accuracy or broader executive composites once multiple comparisons and latent modeling are respected, arguing against simple microstate-WM biomarkers in homogeneous young samples [115,117].

In the study [77], results showed distinct effects across frequency bands. Regarding the theta band, within the female group, lower theta complexity at rest correlated with better working memory performance (r = −0.67, *p* = 0.017). Such a relationship was not found in men. Regarding the lower alpha band (alpha1, 8–10 Hz), women again showed higher values than men across measures, with females displaying stronger desynchronization and greater complexity in this band. Regarding the upper alpha band (alpha2, 10–13 Hz), no significant effects of either task condition or sex were found in this frequency band.

Methodologically, these results highlight that band × direction × topology interactions matter: coupling that is frontal-local and faster (beta/gamma) tends to support maintenance and simple span, while over-synchronized long-range theta along the fronto-parietal axis can hinder sequencing/manipulation; low-frequency coherence along frontal–temporal routes may aid accuracy, and alpha couplings that bridge right posterior to frontal regions can slow responses. WM benefits most from resting networks that are integrated yet flexible, with efficient small-world properties, adaptive variability in alpha-band connectivity, and less persistent slow-band temporal correlations—features that are amplified by cognitive reserve and that deteriorate with aging, primarily when long-range synchronization becomes too rigid or misallocated across bands and axes.

#### 3.3.5. IAF

In the study [105], once the general factor of intelligence was modeled, no additional specific associations were found between IAF and the lower-order ability factors of memory, reasoning, or perceptual speed.

#### 3.3.6. Task Design and Sample Factors That Explain “Mixed” Results

“Mixed” findings across resting-EEG/WM studies are primarily explained by “working memory,” the participants tested, and the EEG measurement method. WM is not a single construct: simple maintenance (e.g., Digit Span Forward) places different demands than manipulation/updating (Reverse/Sequencing, Arithmetic), and both differ from visual/spatial WM (retro-cue arrays, allocentric navigation) and from low-load vigilance surrogates (one-back). Associations with resting EEG are strongest when WM tasks tax manipulation or high-fidelity maintenance, and weakest when the “WM” task is effectively recognition or 1-back. For example, resting measures did not relate to a light one-back card task in older adults even though the same EEG indexed recognition differences on a separate learning task [75]; eyes-closed resting features predicted Digit Span Sequencing (manipulation) via frontal coherence patterns and penalized over-synchronized fronto-posterior theta [95]; frontal PAF tracked the harder reverse span above age/education [78] and even showed state sensitivity to same-day Digit Span [114]; visual WM with retro-cues was best when resting alpha-network dynamics were flexible and efficient [121]; real-world allocentric spatial WM in elder cohorts covaried with microstate engagement that diminishes with age [79]. In children, WM measured by Digit Span aligns positively with frontal theta at rest ([109], echoed multivariately in [112]), whereas in healthy aging, the advantageous profile trends toward less delta/theta and faster alpha, mirroring broader slowing/reserve effects [74,92,113]. Thus, task load, modality, and WM subprocess (storage, manipulation, or spatial updating) critically determine the direction and magnitude of EEG–WM links.

Sample composition further shapes results. Age flips several associations (childhood frontal theta helpful; older-adult slow power often harmful), and cognitive reserve, education, lifestyle, and SES modulate coupling between EEG and behavior. High-reserve or more educated groups show stronger alpha-band connectivity modulation between eyes-open/closed and better spatial-WM strategy scores [90,94]; more active elders show less delta/theta, more alpha, and superior speed/problem-solving, though WM effects are subtler [92]; socially vulnerable mid-life adults exhibit elevated slow-band connectivity and poorer executive scores, indicating that background connectivity can mark control/WM capacity within environmental constraints [125]; children from lower-income families show EEG patterns (notably theta) that predict working-memory differences beyond vocabulary [112]. Conversely, homogeneous, high-ability university samples often yield evidence of range restriction and lower task demands; large, well-controlled cohorts found no reliable WM links for band ratios or microstates after controlling for multiple comparisons and latent models [115,117,128]. Even within aging mega-samples, resting markers (IAF/alpha/aperiodic) may map more strongly to processing speed or interference resolution than to memory per se when memory tasks are less demanding [108], and longitudinally, IAF × aperiodic-slope interactions forecast executive decline more than episodic memory [129].

Measurement choices also drive divergence. Outcome metric matters: some EEG patterns predict speed but not accuracy (e.g., higher left-parietal delta predicting faster responses, while right-parietal alpha/beta predicts lower accuracy in an older delayed match-to-sample WM task) [107]. Topography matters: frontal features (PAF, frontal coherence) are most WM-relevant, whereas excessive fronto-parietal theta can selectively impair sequencing/manipulation [95]. Recording state and length matter: eyes-open vs. eyes-closed produce different baselines and predictive power (in children, eyes-open ratios predicted inhibition/planning while eyes-closed did not [111]); six minutes of eyes-closed rest did not drift toward drowsiness in elders/MCI, supporting the reliability of that duration [93]. EEG feature definitions vary: studies mix absolute vs. relative power and z-scored age norms [74] vs. proportional spectra [76]; band edges differ (e.g., theta 4–7.5 vs. 4–6.5 Hz; delta 0.1–3 vs. 1–4 vs. 2–4 Hz), shifting what “slow power” captures. Some work isolates periodic vs. aperiodic components before inference [104,108], whereas others do not, risking misattribution of power changes to oscillations rather than 1/f background. Hardware and duration vary from wireless 14-channel, 60 s segments at 128 Hz ([107]) to high-density 64–128-channel, multi-minute recordings [80,121,129]; shorter, low-density protocols can still show effects but increase noise and emphasize robust, large-effect markers (e.g., strong parietal slow-power/RT links; [107]). Connectivity metrics differ—simple coherence vs. lagged linear connectivity vs. graph-theoretic efficiency vs. temporal entropy—capturing complementary properties; WM links appear when networks are integrated yet flexible (short paths, high efficiency, variable alpha edges) and falter when coupling is rigid or misallocated (over-synchronized fronto-parietal theta) [95,121,123]. Finally, statistics and correction practices matter: several raw correlations vanish under multiple-comparison control or Bayesian model comparison [115,117,128]; others survive only for manipulation-heavy WM indices. In short, “mixed” results are expected unless studies align WM demands with age/topography, standardize spectral and aperiodic modeling, separate speed from accuracy, choose the right resting state, and analyze connectivity and temporal structure rather than power alone.

### 3.4. Resting-State EEG Correlates of Inhibitory Control

#### 3.4.1. Big Picture

Across paradigms that tax inhibition—Stop-Signal/Go–No-Go, Stroop/flanker interference, antisaccade, and sustained attention with No-Go probes—resting EEG features predict who suppresses actions or interference more effectively. Three families of markers recur: (i) spectral power in theta, alpha, and beta bands; (ii) ratios and lateralization indices (e.g., theta/beta; β/α asymmetry); and (iii) connectivity/topology in frequency-specific networks. Effects are moderated by age/development, recording state/topography, and task demands (motor stopping vs. interference resolution vs. vigilance).

#### 3.4.2. Spectral Power Predictors

##### Theta (4–7/8 Hz)

Higher resting theta can support inhibitory control during conflict resolution. Individuals with higher baseline theta committed fewer false alarms on conflict No-Go trials, whereas those with low theta showed selective breakdowns under audiovisual conflict; the advantage localized to parietal sources (BA7/BA40) and was specific to total (not phase-locked) theta power [82]. Development sharpens this link: in a cross-sectional 8–30-year sample, resting theta rose in childhood and fell with age; from ~10.7 years onward, higher resting theta predicted stronger task-evoked No-Go theta, and from ~19.5 years it also predicted fewer false arms behaviorally—i.e., better inhibition—with increasing strength into adulthood [83]. Not all theta is beneficial: in a cued flanker paradigm, greater resting theta was associated with larger congruency costs (worse reactive control), suggesting theta–inhibition relations invert when the dominant demand is interference resolution rather than outright stopping [101].

##### Alpha/Individual Alpha Frequency (IAF)

Alpha frequency (pace) is a robust trait for inhibitory control in aging. Faster IAF predicted better SART discrimination (a vigilance task with No-Go probes), and IAF mediated the adverse effect of age on SART performance—older adults’ slower alpha partly explained their reduced inhibitory sensitivity [100]. By contrast, alpha power is nuanced and topography-dependent: higher parietal/occipital alpha at rest predicted slower but more stable vigilance (a conservative style) [84] and, in other contexts, frontal/central alpha related to greater variability—patterns that shape inhibition indirectly via speed–stability trade-offs [84].

##### Beta (13–30 Hz)

Resting beta shows band- and region-specific ties to stopping. In a motor-inhibition Stop-Signal Task, higher beta power in bilateral motor cortices, the right somatosensory cortex, and the right inferior frontal cortex predicted longer SSRT (worse stopping); greater beta coherence among these regions and higher beta-band global efficiency also tracked worse inhibition [81]. However, in sustained attention, sub-bands dissociated: mid/upper-beta (20–28 Hz) features in select regions correlated with better error inhibition and more stable performance, whereas lower-beta (12–16 Hz) features were associated with slower responses and reduced vigilance [84]. In oculomotor control, higher resting bias was associated with slower prosaccades in young adults, with no disproportionate antisaccade cost in older adults, implicating beta in motor readiness rather than selective inhibition per se [106].

##### Ratios and Lateralization Indices

Links to executive inhibition are mixed and context dependent. In a large healthy sample, TBR did not predict executive control on the ANT-I (null for the conflict/executive network [89]). In an emotional Go/No-Go, higher TBR was associated with reduced fearful modulation of inhibition (fearful faces less strongly inhibited participants) and lower self-reported attentional control, suggesting vulnerability when affective cues tax inhibitory gating [135]. Under stress, frontal TBR moderated attentional-control decline: higher TBR predicted larger stress-induced drops in control (a proximal risk factor for inhibitory failures), whereas low-TBR individuals were resilient [136]. It should be noted that several TBR studies emphasize reversal learning or risky decision making rather than pure inhibition and show poorer adaptation with higher TBR [132,133,134].

##### Prefrontal β/α Asymmetry

Stable lateralization of fast-to-alpha power over the prefrontal cortex tracks interference resistance. Individuals with left-lateralized β/α (greater left than right) exhibit more minor Stroop effects across verbal and spatial Stroop variants—better domain-general interference suppression localized to left prefrontal regions (pre-SMA, middle/inferior frontal areas) [125]. Related task-switching work shows that left-lateralized β/α in mid-MFG predicts stronger phasic (switch-trial) responses. In contrast, right-lateralized activity predicts stronger sustained control (mixing costs) [131], while mechanisms likely scaffold interference handling.

##### Connectivity, Coherence, and Topology

Network organization at rest constrains inhibitory speed and stability. In the beta band, the Stop-Signal study found that greater inter-regional coherence (motor–somatosensory–inferior frontal) and higher global efficiency predicted longer SSRT—suggesting that a strongly synchronized beta network at rest reflects a motor set that is harder to disengage during stopping [81]. In vigilance with No-Go probes, upper-beta features in certain regions tracked fewer commission errors and more stable performance, whereas temporal gamma connectivity marked lapses and inconsistency; parietal alpha favored stability at the cost of speed [84]. More generally, graph analyses outside strict inhibition paradigms show that globally efficient, small-world architectures predict faster responses, while over-clustering/longer paths and strong global alpha/gamma synchronization predict slower responses [123]—speed constraints that bound inhibitory performance even when error rates are unchanged.

##### Developmental and State Moderators

The theta–inhibition relationship emerges with maturation: only from late childhood does higher resting theta translate into stronger No-Go-related theta and, later, fewer false alarms [83]. In aging, slower IAF helps explain the decline in inhibitory sensitivity [100]. Aperiodic dynamics also reflect state adaptability: individuals with flatter resting spectral slopes (“noisier” brains) showed larger increases in steepness during No-Go vs. Go, indicating on-demand noise suppression under high control, whereas “low-noise” individuals were more stable across conditions [124]—a trait–state interplay that shapes inhibitory efficiency.

##### Boundary Conditions and Nulls

Not all paradigms show resting–inhibition links. Executive network performance on ANT-I was unrelated to the resting theta/beta ratio or delta–beta coupling [89]. A young-adult auditory Go/No-Go study reported no correlations between intrinsic band powers and accuracy/RT/RTV [126]. Interference tasks can likewise yield nulls: neither IAF nor alpha/theta power predicted Stroop/Navon interference in a well-powered sample with Bayesian support for the null [102]. These discrepancies typically reflect lower task demands, homogeneous high-ability samples (range restriction), or feature choice (e.g., band ratios vs. lateralization or connectivity).

##### Synthesis

The resting-state predictors of inhibitory control are frequency-, region-, and network-specific. Faster IAF (especially in aging) and left-lateralized prefrontal β/α support interference suppression; higher baseline theta benefits conflict-laden No-Go inhibition from late childhood on, but can worsen reactive flanker control; beta markers split by context—motor-network beta power/coherence/global efficiency presage worse stopping, while mid/upper-beta in select regions relates to fewer errors and stability in vigilance. Connectivity/topology constraints the speed–stability envelope within which inhibition operates. Globally efficient yet not over-synchronized networks are advantageous. Moderators (age, stress, and affect) tune these relationships, explaining why some studies find strong effects while others, by design, appropriately find none.

### 3.5. Resting-State EEG Correlates of Decision Making

#### 3.5.1. Big Picture

Across value-based, social, and speeded choice paradigms, resting EEG features forecast who decides faster, adapts to changing contingencies, and favors economically “rational” options over fairness or immediacy. Three motifs recur. (i) Slow-to-fast balance: Higher slow-wave prominence (theta/delta) relative to beta—often summarized as an elevated theta/beta ratio—predicts riskier and less adaptive choices, poorer reversal learning, and a stronger pull of immediate rewards [132,133,134,135]; when decisions are modeled as evidence accumulation, more slow power (occipital delta up, parietal theta down) aligns with lower drift rates—slower, less efficient decisions [75]. (ii) Alpha “pace” and network efficiency: Faster/more efficient alpha systems support better decision speed and, in social choice, greater acceptance of payoff-maximizing offers; overly strong posterior alpha tends to trade speed for stability [84,122]. (iii) Topology over power: Integrated yet not over-synchronized networks—shorter paths, higher efficiency, flexible reconfiguration—predict faster, more consistent decisions; rigid, highly clustered, or globally synchronized networks slow responding and reduce adaptability [85,123]. Aperiodic (1/f) structure adds a domain-general layer: steeper slopes (less neural noise) are associated with faster decisions and more efficient control adjustments [80,103,124].

#### 3.5.2. Speeded/Perceptual Choice and Vigilance

Both spectral content and topology constrain decision speed at rest. Steeper aperiodic slopes were associated with faster responses in a multilevel Hick-type reaction-time task [80]. In long sustained-attention runs, upper-beta and gamma power from left central/temporal regions forecast slower, more variable responding. In contrast, stronger parietal/occipital alpha predicted slower but steadier performance—i.e., a conservative speed–stability trade-off [84]. Graph metrics converge to greater global small-world efficiency at rest, which relates to shorter reaction times, while higher overall alpha/gamma synchronization, greater clustering, and longer characteristic path lengths predict slower responses [123]. Signal components map onto decision-stage ERPs: larger resting delta-1 amplitude (eyes closed) predicted shorter RTs, and higher resting alpha (alpha-3) predicted larger task P3b (evidence-accumulation) amplitudes [127]. In older adults performing a delayed match-to-sample, higher left-parietal delta (and marginal theta) related to faster responses but elevated right-parietal alpha/beta/gamma tracked lower accuracy—again illustrating speed–accuracy dissociations by band and topography [107]. Not all speeded paradigms show resting-power links: in an auditory Go/No-Go task with healthy young adults, intrinsic band powers did not predict accuracy, RT, or RT variability [126].

#### 3.5.3. Value-Based and Risky Choice (Reward/Punishment Learning)

Markers of slow-to-fast balance at rest are particularly diagnostic for risky choice. Individuals with higher theta/beta ratios were poorer at reversal learning: they failed to upshift risky choices when advantageous and to downshift them when detrimental [132]. In the Iowa Gambling Task, higher frontal (and parietal) theta/beta—and higher delta/beta—ratios predicted more disadvantageous deck selections across the session [133]; a replication showed the effect was explicitly driven by theta power rather than beta [134]. These findings are consistent with a “slower baseline” prioritizing immediate reward over long-term payoff. Complementing power metrics, network flexibility also matters. Globally elevated resting beta-2 power predicted slower visual-search decisions and worse shooting accuracy, together with stronger, more rigid fronto-parietal/occipito-frontal connectivity that reconfigured less under task demands; conversely, lower beta-2 power accompanied greater reconfiguration and better performance [85]. Recognition decisions fit the same pattern when formally modeled: slower diffusion-drift rates (less efficient evidence accumulation) co-occurred with higher occipital delta and lower parietal theta at rest in older adults [75].

#### 3.5.4. Social Decision Making (Fairness vs. Payoff)

Alpha-band network organization at rest predicted social bargaining style in the Ultimatum Game. Participants with more efficient alpha networks—stronger frontal–occipital clustering, higher global/local efficiency, shorter paths—accepted more offers (higher payoff-maximizing acceptance rates). In contrast, weaker alpha integration (especially along fronto-occipital links) characterized low-acceptance responders [122]. Thus, integration into the dominant resting rhythm appears biased toward economically rational acceptance rather than rejection for fairness-based rejection 

#### 3.5.5. Aperiodic and Alpha-Frequency Markers of Decisional Efficiency

Aperiodic slope behaves as a trait-like decisional efficiency index: steeper slopes are related to faster reaction times [80] and mediated age differences in speeded coding performance on a clinical battery [103]. The hill also adapts state-wise to control demands: individuals with flatter resting slopes (“noisier” brains) showed larger within-trial steepening (noise suppression) during No-Go vs. Go, indicating greater flexibility to stabilize processing when persistence is required [124]. Although alpha peak frequency is most robustly tied to inhibitory control with age [100], its broader interpretation as a temporal sampling rate suggests it may bound decision speed when interference is high.

#### 3.5.6. Connectivity and Topology: “Ready-to-Decide” Networks

Graph-theoretic properties at rest set the decision envelope. Faster choices align with globally efficient topologies (shorter path lengths, higher efficiencies) and are not overly clustered; excessive global synchronization—especially in alpha/gamma—slows decisions [123]. In applied contexts, rigid high-beta networks predict poorer decision performance and reduced task-driven reconfiguration [85], whereas efficient alpha-band integration supports payoff-maximizing social choices [122]. Together, these data argue that decision readiness is better indexed by network wiring than by the amount of power in any single band.

#### 3.5.7. Moderators: Age, Stress, Affect, and Reserve

Age and reserve reshape resting-decision links. Older adults show more slow activity and slower alpha, which aligns with reduced drift/processing speed [75]. At the same time, individuals with higher cognitive/social reserve display stronger alpha-band connectivity modulation between internal/external states—a property associated with better attention and strategy use that likely benefits decision consistency [90,94]. Affective and stress contexts matter: higher frontal theta/beta ratios coincided with reduced fear-based inhibitory bias in an emotional Go/No-Go [135] and predicted larger stress-related drops in attentional control under evaluative pressure [136], both of which can tilt choices toward immediacy or approach. Finally, orienting efficiency in the ANT-I was better in individuals with lower theta/beta ratios [89], consistent with a broader role for slow/fast balance in cue-driven selection during decision episodes.

#### 3.5.8. Boundary Conditions and Methodological Notes

Null or minor effects appear in homogeneous, high-ability young samples and in low-demand tasks [126]; decision readouts split by speed vs. accuracy and by social vs. monetary goals, so band–topography effects can reverse (e.g., posterior alpha supports stability but slows speed [84,107]). Connectivity and topology repeatedly outperform raw power as predictors [85,122,123], and aperiodic slope adds a domain-general efficiency layer [80,103,124]. Recording state (eyes open vs. closed), band definitions, and separating periodic from aperiodic components influence inferences. In aggregate, resting EEG markers of decision making are best conceived as (a) a slow/fast balance that biases risk and adaptation (theta/delta ↑ → riskier, less adaptive), (b) alpha-system pace/integration that sets speed–stability and payoff-rationality tendencies, and (c) network efficiency/flexibility that gates how rapidly and consistently evidence can be accumulated and acted upon.

### 3.6. Resting-State EEG Correlates of Cognitive Flexibility (Set-Shifting)

#### 3.6.1. Big Picture

Cognitive flexibility—the ability to update task sets, shift rules, and maintain goals proactively—shows reliable resting-EEG signatures distinct from, yet complementary to, those for working memory, inhibition, and decision making. Three motifs recur: (i) frontal spectral “tone” and alpha pace support proactive, rule-maintenance aspects of flexibility; (ii) lateralized prefrontal fast-to-alpha balance biases individuals toward phasic (switch-trial) versus sustained (mixed-block) control; and (iii) network organization at rest (coherence/topology) constrains how quickly sets can be reconfigured. Age and reserve systematically modulate these links.

#### 3.6.2. Spectral and Aperiodic Markers of Flexibility

##### Faster Alpha Pace Benefits Interference-Prone Set Management

In a large aging cohort, interference resolution (Stroop incongruent performance) scaled positively with individual alpha peak frequency (IAF), with effects centered over right frontal and bilateral parietotemporal cortex—consistent with faster temporal sampling aiding separation of competing task rules [108].

##### Parietal Low-Alpha Power Tracks Set-Shifting Success on WCST

Higher resting alpha-1 (8.6–10.2 Hz) over bilateral parietal sites (EO) predicted achieving more categories and fewer perseverations on the Wisconsin Card Sorting Test, linking posterior “idling” at low-alpha to a strategic baseline that supports rule discovery and maintenance [120].

##### Beta/Alpha Level and 1/f Background Relate to Proactive Control

Individuals with higher resting alpha power and larger aperiodic (1/f) offset showed stronger proactive control (cue-based goal maintenance) in a cued-flanker paradigm. In contrast, theta was associated with larger congruency costs (poorer reactive control) [101].

##### Right-Frontal High-Beta Indexes Nonverbal Set-Shifting

A greater high-beta magnitude at F8 correlated with faster completion of a figure-based trail-making switch (FTMT Part D), a nonverbal analog of the TMT-B targeting cognitive flexibility [130].

##### Processing-Speed/Flexibility Composite Markers

Central delta power in older adults positively tracked TMT-B performance (faster set-switching under sequencing demands), suggesting slow-wave tone can serve a compensatory role in late life for multi-step switching/inhibition blends [106].

##### More Theta and Better Cognitive Flexibility

In the study [119], theta power was positively associated with category fluency performance: individuals with higher resting theta tended to produce more animal names. No meaningful association was found between theta power and letter fluency. However, when controlling for theta power, the relationship between age and fluency decline remained unchanged. This indicated that theta power did not moderate or explain the link between age and reduced verbal fluency, even though it was related to performance level.

#### 3.6.3. Prefrontal Asymmetry: A Lever on Control Mode

Lateralized fast-to-alpha balance in the prefrontal cortex provides a trait-like bias for how people implement flexibility. Left-lateralized β/α in mid-MFG predicted smaller switching costs (better phasic, trial-wise reconfiguration). In contrast, right-lateralized β/α predicted smaller mixing costs (better sustained goal maintenance across mixed blocks) across three task-switching paradigms [131]. The same left-dominant β/α asymmetry also generalized to smaller Stroop costs across verbal and spatial variants (more efficient criterion setting), pointing to a domain-general mechanism for interference-resistant shifting [116].

#### 3.6.4. Connectivity and Topology: “Ready-to-Shift” Networks

Flexibility is best indexed by how networks are wired at rest, not just how much power they carry.

##### Frontal Coherence Benefits Executive Speed/Flexibility

Higher within-frontal delta coherence predicted faster TMT-B (set-switching) alongside advantages on other executive indices [95].

##### Global Efficiency > Global Synchronization

Graph studies show that shorter characteristic paths and higher small-world efficiency at rest are associated with faster responses. However, overly clustered, strongly synchronized alpha/gamma networks slow behavior—constraints that naturally limit how quickly rule sets can be swapped [123].

##### Flexibility Thrives on Reconfigurability

Individuals with more rigid, high-beta networks at rest showed poorer performance and less task-driven reconfiguration across attention/visuomotor contexts. In contrast, lower beta-2 power coupled with greater reconfiguration accompanied better performance—an architectural hallmark of cognitive flexibility [85].

##### Reserve-Related Coupling Supports Strategic Adaptation

Higher cognitive/social reserve aligned with stronger low-alpha long-range connectivity and state-dependent modulation (EO ↔ EC) and with better strategy use—network properties that likely scaffold efficient task-set updating [90,94].

#### 3.6.5. Development, Age, and Reserve as Moderators

Alpha pace slows with age, which partly explains decreases in interference-laden control [108]; conversely, education/reserve bolsters connectivity modulation and executive outcomes [90,94]. In late life, selective slow-band features (central delta) can mark compensatory support for complex switching blends [106], while in younger adults, proactive control relates more to posterior alpha and 1/f offset [101].

#### 3.6.6. Boundary Conditions and Nulls

Not all resting features generalize. In a large young-adult sample using a brief EF battery (including switching), classical spectral measures, ratios, and asymmetries showed strong Bayesian evidence for no reliable correlations, likely reflecting homogeneous high ability, modest task demands, and feature choice [128]. Microstate metrics likewise showed weak or null links to broad EF composites in healthy young adults [117].

#### 3.6.7. Synthesis

A conservative, cross-study profile for resting predictors of cognitive flexibility is as follows: faster alpha pace (higher IAF) and stronger parietal low-alpha support proactive goal maintenance and WCST-style set discovery; left-lateralized prefrontal β/α favors agile, phasic switching, while right-lateralized β/α favors sustained set maintenance; within-frontal coherence (especially low-frequency) and globally efficient—but not over-synchronized—networks provide the wiring for rapid reconfiguration; and in aging, selective slow-band tone and higher reserve can partially compensate. Together, these markers outline a trait-like “ready-to-shift” brain state that is separable from working memory storage, motor stopping, and reward-driven choice yet interacts with them when tasks blend demands.

### 3.7. Resting-State EEG Correlates of Sustained Attention (Vigilance and Stability)

#### 3.7.1. Big Picture

Sustained attention—the capacity to maintain goal-directed monitoring over extended periods and to keep response variability low—shows a distinct resting-EEG signature that is not reducible to working memory, inhibition, decision speed, or set-shifting. Three themes recur: (i) alpha “pace” shapes sensitivity vs. conservatism, (ii) high- vs. low-frequency power dissociate fast-but-variable from slow-but-stable response styles, and (iii) network efficiency and reconfigurability constrain both average vigilance and moment-to-moment stability.

#### 3.7.2. Spectral Power: Speed–Stability Trade-Offs

Resting alpha and high-frequency activity map onto different vigilance styles. In a 105 min SART, greater parietal/occipital alpha was associated with slower yet more consistent performance (conservative monitoring). In contrast, elevated upper-beta and gamma (left central/temporal) forecast slower and more variable responding and more lapses (temporal gamma in particular) [84]. Lower-beta (12–16 Hz) tended to accompany slower responses and lower vigilance, while mid/upper-beta features in some regions related to better error control and stability—highlighting band-specific roles rather than a unitary “beta effect” [84]. Outside prolonged vigilance, globally higher resting beta-2 power predicted worse attention in visual search and degraded real-world target accuracy (shooting), together with rigid connectivity that reconfigured poorly during task demands [85].

In the study [86], the EEG analysis revealed clear relationships between resting-state brain activity and performance on the attentional task. Specifically, higher absolute alpha power during rest—especially in posterior, occipital regions of the brain—was associated with greater attentional breadth. In other words, participants whose brains exhibited stronger alpha oscillations at rest tended to favor global, big-picture processing. In contrast, higher beta power at rest, particularly in right parietal and right occipital regions, was associated with narrower attentional breadth. These individuals were more inclined to focus on local details rather than on the overall structure. Importantly, theta activity did not significantly predict attentional breadth, suggesting that this frequency band is less relevant to global versus local attentional preferences. The researchers also conducted analyses that controlled for correlations between different frequency bands, since power in alpha, beta, and theta can covary across individuals. Even after statistically isolating the unique contribution of each band, the same pattern emerged: greater resting alpha power predicted broader attentional breadth, while greater resting beta power predicted narrower attentional breadth.

#### 3.7.3. Alpha Pace (IAF) and Attentional Sensitivity

Faster individual alpha frequency supports finer discrimination in sustained attention with inhibitory probes: higher IAF predicted better SART sensitivity in older adults, and IAF statistically mediated part of the age-performance relationship, implicating alpha pace as a mechanism linking aging to vigilance declines. Notably, this effect did not generalize to all sustained-attention tasks (e.g., no parallel IAF–RVP link), underscoring task specificity within the vigilance domain [100].

#### 3.7.4. Connectivity and Topology: Wiring for Vigilance

Vigilance benefits from networks that are efficient but not globally over-synchronized. Graph work shows that shorter path lengths and higher small-world efficiency at rest predict faster responses. In contrast, strong global alpha/gamma synchronization, high clustering, and longer paths predict slower responding—an architecture that naturally bounds sustained attention speed without necessarily improving stability [123]. In applied attention contexts, rigid high-beta networks at rest (strong fronto-parietal and fronto-occipital coupling) portended poorer performance and less adaptive reconfiguration when task demands changed [85]. Reserve-linked coupling patterns align with better vigilance: higher cognitive reserve was associated with stronger long-range low-alpha connectivity at rest and superior sustained-attention sensitivity (RVP A′), suggesting that state-gated alpha integration supports efficient monitoring [90].

#### 3.7.5. Predicting Vigilance Stability (Not Just Mean Level)

Significantly, resting features also predict variability, not only averages. In the long SART, small subsets of parietal alpha, mid-/upper-beta, and gamma features jointly predicted both average vigilance (CVS) and trial-to-trial variability (RTV), with upper-beta/gamma most informative for instability and lapses [84]. Thus, baseline high-frequency tone is a practical marker of who will struggle to sustain consistent performance over time.

#### 3.7.6. Moderators and Boundary Conditions

Age slows IAF and shifts the speed–stability balance toward slower monitoring; faster IAF partly counters this [100]. Lifestyle/reserve factors modulate coupling strength, associativity, and laterality to sustain attention better [90]. Not all vigilance paradigms tap the same neural levers: SART (with inhibitory No-Go probes) shows strong IAF links, whereas continuous sequence-detection (RVP) is more tightly tied to reserve-related connectivity than to alpha pace per se [90,100]. Finally, when attentional demands are brief and accuracy near the ceiling, resting power–performance links may vanish, indicating that sustained time-on-task is crucial for these associations to surface.

#### 3.7.7. Synthesis

A cautious, cross-study profile for sustained attention is as follows: (a) higher parietal/occipital alpha at rest favors stable (if slower) monitoring, while elevated upper-beta/gamma—primarily temporal—signals vulnerability to lapses and variability; (b) faster alpha pace (IAF) enhances inhibitory-laden vigilance sensitivity in aging; and (c) efficient, reconfigurable networks (short paths, modest clustering) support better vigilance, whereas rigid, high-beta coupling forecasts poorer attention and limited adaptability. Together, these markers define a “ready-to-monitor” resting state distinct from memory storage, stopping, value choice, or set-switching, specifically forecasting who can keep attention steady—and who will drift—over prolonged, monotonous demands.

### 3.8. General, Mixed Executive Functions

In the study [118], cognitive performance was assessed through a neuropsychological test battery. These tests measured several domains, including short-term and long-term memory, attention, verbal fluency, cognitive flexibility, crystallized intelligence (knowledge-based abilities), and fluid intelligence (reasoning and problem-solving skills). The researchers assessed cognitive performance using a neuropsychological test battery drawn from the LEMON dataset. Five specific tests were included, each targeting different aspects of cognition. The California Verbal Learning Task (CVLT) was used to measure both short-term and long-term memory. The Trail Making Test (TMT) served as an index of cognitive flexibility. To evaluate crystallized intelligence, which reflects accumulated knowledge and vocabulary, the researchers employed the Wortschatztest (WST). Fluid intelligence was measured using Subtest 3 of the Leistungsprüfsystem-2 (LPS-2), a reasoning-based assessment. Finally, verbal fluency, or the ability to produce words under specific constraints, was examined through the Regensburger Wortflüssigkeitstest (RWT). Although the full LEMON dataset included the Test of Attentional Performance (TAP), which measures aspects of attention such as alertness, inattention, and working memory, this study did not include TAP results. Instead, it focused on memory, flexibility, intelligence, and fluency as the core cognitive domains of interest. The analyses revealed several meaningful correlations between EEG microstate indices and the results of the cognitive tests. For the LPS-2, which measured fluid intelligence, scores were significantly associated with the mean duration of microstate A (positively correlated) and with the transition probabilities between microstates D and C, and C and D (tandemly correlated). This means that participants who showed greater stability in microstates A tended to perform better on fluid intelligence tasks. At the same time, frequent switching was associated with poorer reasoning. RWT also showed consistent relationships with microstate A. Specifically, the mean duration, occurrence, and time coverage of microstate A were all positively correlated with RWT scores. Thus, individuals whose EEG patterns showed longer-lasting, more frequent episodes of microstate A exhibited higher verbal fluency. For the WST, which assessed crystallized intelligence, significant correlations were found with the occurrence of microstate B (positively correlated) and with the transition probabilities from microstate E to B (positive) and from E to C (negative). In addition to these direct relationships, regression analyses confirmed that the identified microstate measures could significantly predict test outcomes. The mean duration of microstate A predicted both RWT and LPS-2 scores, while its occurrence and coverage specifically predicted RWT performance. Transitions between microstates D and C predicted LPS-2 scores, the occurrence of microstate B predicted WST results, and transitions from microstate E to B and from E to C also contributed to WST prediction. Regression analyses confirmed that these microstate indices could significantly predict scores on cognitive tests, such as the Regensburger Wortflüssigkeitstest (verbal fluency), the Leistungsprüfsystem (fluid intelligence), and the Wortschatztest (crystallized intelligence).

### 3.9. Development and Aging Moderators

Across development, the resting EEG–cognition link is not static; it changes sign, bandwidth, and topography with maturation, normal aging, cognitive reserve, and risk status. In early–middle childhood, resting rhythms reorganize rapidly: theta power decreases while alpha power and peak alpha frequency (APF) increase, and the theta/beta ratio (TBR) falls—each tracking gains in executive function. Children aged from three to nine years show higher eyes-closed theta/alpha that gradually shifts toward faster alpha with age, and lower TBR is already associated with stronger EF on the Minnesota Executive Function Scale, even after controlling for age and verbal ability [110]. In school-age cohorts, socioeconomic context shapes the same markers: higher posterior alpha accompais associated with vocabulary, and stronger frontal theta accompais associated with digit-span working memory; lower-SES children tend to show the opposite pattern [109]. Machine-learning analyses in 8–15-year-olds likewise singled out theta (4–6 Hz) as the band most predictive of working-memory ability and income status [112]. By 7–9 years, frontal alpha-to-theta and beta-to-theta ratios during eyes-open rest explain unique variance in inhibition and planning beyond age and naming speed, but do not robustly predict 1-back performance—underscoring that, early on, resting ratios map most cleanly onto control rather than storage [121].

Through adolescence → young adulthood, alpha timing becomes a central moderator. In a lifespan sample (11–70 y), frontal APF independently predicted reverse digit span, with a +1 Hz ≈ +0.21-digit increase, over and above age, sex, and education; APF itself slowed most strongly frontally with age [78]. Resting theta–alpha power ratios can flip their cognitive meaning across age: in a 20–29 vs. 70–79 year olds comparison, a higher theta/alpha ratio (TAR) related to better short-term memory in the young but to worse reasoning in the old—an explicit age-dependent sign reversal [97]. For inhibitory control, the resting–task theta link matures nonlinearly: in a cross-sectional 8–30 y sample, baseline theta began to predict No-Go-related theta at 10.7 years (neural coupling) and only later (19.5 years) did higher resting theta begin to predict fewer false alarms (behavior), indicating that the EEG–behavior mapping itself is age-gated [83].

In healthy aging, several periodic and aperiodic shifts reshape associations. Multiple cohorts show IAF slowing with age and robust behavioral ties: in older adults, faster IAF predicts better SART discrimination and mediates part of the age effect on sustained attention, whereas the same study found no IAF/RVP association and no aperiodic (slope/offset) links to vigilance after correction—arguing that vigilance costs are driven by rhythmic tempo rather than 1/f background [100]. An extensive late-life study that separated periodic from aperiodic content found that interference resolution (Stroop) scaled positively with IAF across right frontal and bilateral parietotemporal sites; processing speed improved with lower right-frontal alpha power; and episodic memory showed no reliable links to alpha or 1/f once separated [108]. Across adulthood, aperiodic exponent and offset decline; higher offset predicted better verbal fluency, and higher exponent predicted better fluency and executive composites independent of age [104]. Yet education moderates these associations: among older, low-education adults, slopes/higher offsets are associated with better attention and working memory, whereas among older, high-education adults, a higher exponent is associated with better processing and lower WM, a sign flip consistent with reserve-dependent operating points [87]. Longitudinally, lower baseline APF predicted greater 10-year decline (especially executive function), while aperiodic exponent tracked baseline executive ability but not change; APF × exponent interactions dominated prognosis—mismatched profiles (high exponent + low APF or vice versa) declined most, “matched” profiles remained stable [129].

Aging also alters network organization and microstates, with behavioral consequences. Older adults show reduced occurrence/variance of microstates C, C′, D, and lower transition probability into these maps, alongside poorer allocentric spatial working memory; they also display lower global explained variance and reduced map occurrence, especially in eyes-open recordings [79]. In a larger two-group sample (20–35 vs. 59–77 y), age strongly predicted microstate parameters, whereas working-memory accuracy did not reliably covary with microstates—suggesting that microstate differences are age-dominant traits rather than WM readouts in healthy adults [115]. Resting topology shifts with age as well: in oculomotor and cognitive batteries, older adults showed slower APF, greater beta power, and reduced parietal alpha asymmetry; higher central delta in older adults related to better set-shifting/inhibition on Trails-B (possible compensation), while in the young, higher beta predicted slower prosaccades (beta-weighted motor set) [106]. In an older WM sample, greater alpha coherence between right parietal–left frontal predicted slower responses, whereas delta/theta frontal–temporal coherence predicted higher accuracy, a band-dependent speed–accuracy dissociation uncommon in young adults [107]. Basic spectral aging effects can also diverge from stereotypes: one 20–91 y study reported declines in delta/theta power with age in healthy adults, while emergent delta increases over two years tracked learning decline and CSF AChE reductions—linking slow-wave increases to incipient cholinergic dysfunction rather than normal aging [91].

Cognitive reserve (CR) systematically reshapes aging EEG–cognition couplings. Higher CR (education/IQ/occupation) is associated with greater low-alpha long-range connectivity (occipital → distributed cortex) during eyes-closed rest and better sustained attention and spatial WM; high-CR individuals also show stronger eyes-open vs. eyes-closed modulation of alpha/theta connectivity—more effective state gating between internal and external modes [90]. In midlife–older adults (45–64 y), high CR was linked to greater coherence and larger EO–EC differences, with the hemispheric asymmetry of coherence flipping across age; high-CR participants also performed better on Digit Span and verbal fluency [94]. Spectrally, high-CR older adults exhibit lower delta/theta power (parietal/occipital/temporal) than low-CR peers across EO/EC, despite similar neuropsychological scores—a latent neural efficiency pattern that standard tests may miss [113]. Lifestyle covaries similarly: older adults with higher incidental physical activity show less delta/theta, more alpha, faster mean frequencies, and better speeded IQ subtests, whereas less active peers show a “slowed” frontal–temporal profile [92].

Finally, risk and preclinical states modify resting predictors. In MCI vs. healthy aging, posterior theta is reliably higher in MCI and correlates negatively with global cognition in controls; six-minute recordings remain stable without progressive drowsiness, supporting feasibility in older cohorts [93]. In longitudinal geriatric samples spanning AD/MCI/controls, higher baseline eyes-closed theta and reduced alpha reactivity predicted poorer global and executive outcomes ~20 months later, beyond age and baseline scores [98]. In older adults at risk for dementia but still “healthy,” baseline global EEG coherence correlated with global cognition. However, neither coherence nor slowing predicted five-year decline—likely underpowered due to attrition of those who declined the most [99]. Across adulthood, aperiodic slopes/offsets and IAF also track executive speed rather than episodic memory [104,105,108], and ratio markers adopt context-specific meanings with age: in children and teens, lower TBR indexes better EF [110,111]; in young adults it predicts orienting efficiency and resilience to evaluative stress [90,136]; in older adults, IAF—not TBR—more consistently constrains vigilance [100].

## 4. Discussion

Executive functions encompass a range of cognitive functions that enable effective daily functioning. Any decline in these functions imposes limitations across the educational, social, and professional domains. Resting-state EEG is routinely used in clinical practice and neuroscience to measure bioelectrical activity related to task performance. This systematic and mechanistic review aims to synthesize and analyze studies measuring resting-state EEG and executive function. Sixty-three studies were included, demonstrating the considerable interest of researchers in this topic. The studies varied considerably. They used different EEG and executive function measurement paradigms, and participants were from various age groups (all healthy). A discussion of the findings from the included studies is presented below.

### 4.1. What a “Resting Brain” Can (and Cannot) Tell Us About Executive Function

A resting EEG provides a trait-like portrait of executive readiness: it captures tempo, noise, and wiring features that help explain why some people are consistently faster, more accurate, or more stable on executive tasks. Most concretely, resting signals can rank-order performance within specific facets. Faster individual/peak alpha frequency tends to accompany stronger interference control and better vigilance sensitivity in aging; manipulation-heavy working memory benefits when frontal alpha pace is quicker and when within-frontal beta/gamma coherence is stronger, whereas excessive fronto-posterior theta coherence selectively undermines sequencing and updating. Decision speed and consistency are better indexed by network organization than by raw power: topologies with shorter path lengths and higher global efficiency support quicker, more reliable choices, while globally heightened alpha/gamma synchronization—or rigid, high-beta networks—slow responses and limit adaptability. For cognitive flexibility, the lateralized prefrontal fast-to-alpha balance acts as a left-dominant control-mode bias. Elevated left-prefrontal β/α relates to nimbler phasic switching, while right-dominant β/α relates to stronger sustained set maintenance across mixed blocks.

These associations are interpretable through speed–accuracy trade-offs that the resting state already foreshadows. Features that favor speed include a faster alpha pace, a more globally efficient topology, and, under some aging conditions, higher left-parietal delta, which shortens reaction times. Features that favor accuracy and stability include stronger low-frequency frontal–temporal coherence in older adults and higher parietal/occipital alpha that steadies monitoring at the cost of speed. Costs emerge when coupling is misplaced: elevated right-parietal alpha/beta often tracks poorer working-memory accuracy, excessive fronto-posterior theta impairs manipulation, and global alpha/gamma synchronization, despite being “strong,” is behaviorally sluggish. Beyond rhythms, the aperiodic backdrop (the 1/f exponent and offset) offers a domain-general index of neural efficiency: steeper slopes and higher offsets, especially when modeled separately from oscillations, tend to align with faster processing and stronger executive composites. Development and aging systematically reshape these links—frontal theta can benefit children’s working memory, but becomes a liability in healthy aging. A slower alpha pace in later life magnifies the association between resting markers and speeded control. Cognitive reserve and education further tilt the picture by enhancing alpha-band long-range integration and state gating (eyes open ↔ eyes closed) and can even invert the direction of some 1/f–behavior relations. In the longer term, combinations of rhythmic pace (alpha frequency) and aperiodic slope appear more prognostic for executive trajectories than either alone; mismatched pace–noise profiles tend to fare worst.

Equally important are the limitations of resting EEG. It cannot, on its own, diagnose executive dysfunction or specific clinical conditions, as single features or simple ratios (such as theta/beta) lack specificity and show strong age- and context-dependent reversals. Resting EEG also cannot capture trial-by-trial strategies or moment-to-moment control policies, because it reflects baseline neural readiness rather than online, task-driven adjustments. Scalp-level power measures do not allow unambiguous source localization, and power-only analyses that ignore the aperiodic component confound oscillatory activity with changes in the background spectral slope. Moreover, findings from resting EEG do not readily generalize across tasks, age groups, or samples unless key moderators are considered—for example, maintenance versus manipulation processes, stopping versus interference control, children versus older adults, or high- versus low-cognitive-reserve cohorts. Finally, resting EEG features cannot compensate for poor behavioral psychometrics, as low task reliability inherently constrains brain–behavior correlations.

Practically, if the aim is trait-level prediction, the most informative resting markers are a compact composite: individual/peak alpha frequency, aperiodic slope and offset, supportive within-frontal beta/gamma coherence (with fronto-posterior theta treated as a risk factor for manipulation), alpha-band network efficiency that favors integration without global over-synchrony, and prefrontal β/α asymmetry that signals a person’s default balance between phasic switching and sustained maintenance. Interpreted together—and explicitly modeled against age, reserve, task demands, and periodic–aperiodic structure—these features yield a precise, actionable account of what the resting brain can tell us about executive function: not diagnosis or momentary tactics, but a stable readiness profile that constrains how quickly, accurately, and consistently executive control can be deployed.

### 4.2. Inhibitory Control

Resting-state EEG shows a coherent pattern of associations with inhibitory control once task family, age, and network topology are considered. Motor stopping in particular scales with beta activity: individuals with higher beta power at rest in motor and inhibitory hubs (bilateral motor and somatosensory cortices and right inferior frontal cortex) exhibit longer stop-signal reaction times, and stronger beta-band coherence and network “efficiency” across these regions are likewise linked to poorer stopping—an instance in which greater organization in the wrong frequency band is maladaptive for inhibition. Theta plays a more conditional role. Higher baseline theta can buffer performance under cross-modal conflict in Go/No-Go paradigms—participants with low resting theta commit more false alarms when visual and auditory signals conflict, whereas those with higher theta are protected—and across development, the coupling between resting theta and No-Go-evoked theta strengthens with age and eventually predicts behavior. Yet in flanker paradigms with reactive interference, more resting theta is associated with larger congruency costs, indicating poorer reactive control. Alpha power itself is not a reliable marker of inhibition. Still, the alpha rhythm’s pace is faster at the individual/peak alpha frequency (IAF), which relates to better sustained attention and withholding on the SART and mediates age-related declines in that domain. Beyond oscillations, aperiodic spectral structure captures a stability–flexibility trade-off. Flatter slopes (more “neural noise”) at rest characterize older adults and lower executive efficiency. At the same time, during conflict tasks, exponents rise from pre-trial to within-trial and are higher for No-Go than Go, especially in those with noisier baselines—evidence of adaptive noise suppression when control demands increase. Lateralized balance in prefrontal dynamics also matters; greater left-lateral β/α activity predicts smaller Stroop costs (better interference resistance), whereas right-lateralization in overlapping regions supports sustained, ongoing control; the same mid-middle frontal gyrus biases control “mode” depending on asymmetry direction. Finally, slow/fast ratios that up-weight slow activity behave like liability markers. A higher frontal theta/beta ratio consistently forecasts poorer reversal learning, riskier and disadvantageous choices on the Iowa Gambling Task, reduced fearful modulation of stopping in emotional Go/No-Go, and larger stress-induced drops in attentional control, even where classic executive-control indices show nulls; lower ratios, conversely, are tied to more efficient orienting in the ANT.

These band-specific findings are mirrored at the network level. Beta-band over-synchronization is again unfavorable: greater resting coherence among bilateral motor/somatosensory cortices and inferior frontal cortex, and higher beta global efficiency, predict longer SSRTs. Slow-band hyper-connectivity shows a similar risk profile outside the motor system. Adults exposed to socioeconomic adversity exhibit elevated delta/theta connectivity at rest and poorer executive screening scores, with education partly buffering these effects. In contrast, reserve-related advantages appear as more flexible, state-sensitive coupling in low-alpha: individuals with higher cognitive or social reserve show stronger long-range low-alpha connectivity at rest and larger eyes-open vs. eyes-closed modulation, accompanied by better attention/working-memory strategy use. More generally, graph-theoretic topology at rest constrains speeded control: globally efficient, small-world organization predicts faster responses, whereas globally stronger alpha/gamma synchronization with high clustering and long path lengths predicts slower responses—constraints that help explain speed–accuracy trade-offs in inhibition tasks as well.

Development and aging reshape these links. In childhood, resting theta is high and, before roughly 10–11 years, is not reliably coupled to No-Go-related theta; through adolescence, that coupling emerges, and by early adulthood, higher resting theta also predicts fewer false alarms, indicating a maturing baseline-to-task linkage for inhibition. SART performance declines primarily in older adulthood via slowing IAF; faster IAF mediates better inhibitory discrimination despite age. Some slow-wave increases may compensate for demanding switching/inhibition tasks, and beta–behavior relations can shift with age and response modality. Large aging cohorts further suggest that resting EEG markers map more robustly onto interference resolution and processing speed than episodic memory, emphasizing their executive rather than mnemonic sensitivity.

Boundary conditions are essential. Not every inhibition task “reads out” from rest in healthy young adults: broad spectral measures often fail to predict accuracy, mean RT, or RT variability in simple auditory Go/No-Go paradigms, and classic Stroop/Navon interference can be insensitive to IAF, alpha/theta power, or 1/f metrics in homogeneous student samples; microstate features likewise show little relation to composite executive performance in such cohorts. In contrast, paradigms with explicit stopping demands (SST), prolonged vigilance (SART), or cross-modal conflict are more likely to reveal resting-state associations.

Taken together, the most defensible picture is that resting beta is a negative marker for motor stopping, theta confers context-dependent benefits (protective under conflict-laden No-Go but detrimental for reactive interference), alpha frequency—not power—indexes inhibitory readiness especially in aging, and the aperiodic slope tracks the capacity to stabilize neural activity when control is required. Elevated theta/beta ratios and slow-band hyper-connectivity flag vulnerability to poor adaptation, risky decision making, and stress-sensitive attentional lapses, whereas left-lateral prefrontal β/α asymmetry, faster IAF, flexible low-alpha coupling, and globally efficient small-world topology indicate stronger inhibitory control. Socioeconomic context and cognitive reserve reliably modulate these baselines, underlining that both neural architecture and life experience shape how the idling brain supports—or hinders—our ability to stop and resist interference.

### 4.3. Working Memory

Across studies, the most consistent resting-EEG predictor of working-memory (WM) capacity is the pace of the alpha rhythm rather than its amplitude. A faster frontal peak/individual alpha frequency (IAF) explains additional variance in manipulation-heavy span beyond age, sex, and education. In a lifespan sample, each 1 Hz increase in frontal IAF predicted ≈+0.21 digits on reverse digit span (the more complex, manipulation condition). IAF also carries a short-term, state component: baseline PAF recorded immediately before testing predicts same-day digit-span performance (but not performance on a different day), and low-PAF individuals tend to increase PAF after the task toward a more optimal range, consistent with a preparedness signal rather than a fixed trait alone. By contrast, alpha or theta power at rest is an unstable correlate of WM once periodic activity is separated from the aperiodic background and age is modulated. In large samples, alpha frequency relates more robustly to executive composites than band-power measures relate to WM per se, and IAF aligns primarily with general ability (g) rather than with a WM-specific factor once latent structure is accounted for.

Theta effects are strongly age- and task-dependent. In healthy older adults and mixed adult samples, higher resting theta—especially at frontal leads—tends to track poorer WM (e.g., negative associations with WAIS-III Working Memory; higher theta in MCI; inverse links with global cognition on the MoCA) and does not predict simple one-back performance even when recognition decisions show clear EEG–behavior relations. Temporal structure in slow bands is also informative: stronger long-range temporal correlations (higher DFA exponents) in theta/delta at rest are inversely related to WAIS-IV WM (Digit Span, Arithmetic) independent of power and scalp topography, suggesting that “stickier,” more correlated slow fluctuations at rest are a liability for WM. In children, the picture reverses: higher frontal theta is positively associated with digit-span WM and partially mediates socioeconomic disparities in WM; lower-SES cohorts show reduced theta alongside weaker WM. Age interactions also appear for ratios: in young adults, a higher theta/alpha ratio is associated with better short-term memory, whereas in older adults this relation is absent or even reversed (notably for reasoning).

Connectivity and topology sharpen these relationships beyond local amplitude. Higher resting beta coherence in frontal networks predicts better Digit Span Forward and Sequencing, and frontal gamma coherence predicts better Forward Span—evidence that locally synchronized fast activity in control hubs supports maintenance and serial ordering. Critically, greater fronto-posterior theta coherence is linked to worse Digit Span Sequencing, indicating that over-binding long-range circuits at slow rates hampers manipulation/updating even when frontal fast-band coupling is beneficial. Convergent results in community-dwelling older adults show that accuracy on a delayed match-to-sample WM task improves with stronger delta/theta fronto-temporal coherence, while higher right-parietal alpha/beta and right-frontal gamma power predict lower accuracy; increased alpha coherence between right parietal and left frontal sites slows responses—an instance where posterior high-frequency engagement and long-range alpha synchrony are maladaptive for WM precision and speed. Beyond static coupling, alpha-band network dynamics at rest—greater temporal variability (higher entropy) and more efficient long-range integration—predict higher accuracy in retro-cued visual WM, with cross-validated models reaching r ≈ 0.75 (RMSE ≈ 0.03) and replication in source space; effects scale with memory load, highlighting a capacity-sensitive signature.

Macro-state and aperiodic markers add boundary conditions. In aging, reduced occurrence and transition probability of microstate maps C/C′/D accompany poorer allocentric spatial WM and diminished engagement of attention/WM-related networks. In contrast, in healthy adults, canonical microstate parameters show no reliable association with 2-back accuracy after multiple-compensation correction. Aperiodic exponent/offset parameters relate to broad executive quality (including WM) across adulthood, but their direction in older adults is moderated by education: with lower education, steeper slopes and higher offsets predict better visual attention and WM; with higher education, steeper slopes predict slower processing and reduced WM capacity—an interaction consistent with reserve-related strategy differences. Over longer intervals, alpha pace and aperiodic slope interact to forecast executive change: “matched” profiles (high–high or low–low) show relative stability, whereas mismatched pace–noise pairs exhibit the most significant decade-long decline, with WM embedded in that executive composite. Lifestyle proxies of reserve point in the same direction: greater cognitive/social reserve is accompanied by stronger low-alpha long-range connectivity at rest and better spatial-WM strategies, even when behavioral means are similar.

Not all WM tasks are “read out” from the rest. Recognition-style one-back shows null EEG–WM associations, with diffusion-model drift in recognition memory correlating with occipital delta (higher) and parietal theta (lower) power, demonstrating a process dissociation between speeded recognition and WM updating. In homogeneous young-adult samples, broad spectral summaries, ratios, asymmetries, and even IAF often fail to correlate with WM indices or N-back accuracy, underscoring the need to separate periodic from aperiodic structure and to use network-level metrics when effects are subtle.

In synthesis, faster frontal alpha frequency, economical and flexible alpha-band topology, and restricted fronto-posterior theta binding characterize stronger WM—particularly manipulation and sequencing—whereas elevated adult theta power, higher fronto-posterior theta coherence, and posterior alpha/beta/gamma over-engagement mark risk for slower, less accurate WM. In children, higher frontal theta is supportive; in later life, the combination of alpha pace with aperiodic slope, together with cognitive reserve, best constrains who preserves versus loses WM capacity over time.

### 4.4. Decision Making

Resting-state EEG relates to decision making through two partially independent channels: (i) signals that set the rate and stability of evidence accumulation, and (ii) network features and band ratios that bias choice policy under reward–punishment trade-offs and contingency changes.

For evidence accumulation and decisional speed, posterior slow bands and alpha-band network efficiency are most informative. In older adults reporting subjective decline, a lower diffusion-model drift rate on a recognition task co-occurred with higher occipital delta and lower parietal theta power at rest, whereas these same EEG features were unrelated to one-back working memory, indicating a process-specific link to speeded recognition decisions rather than a generic memory load. Eyes-closed alpha-band topology further constrains decisional behavior: shorter characteristic path length and higher clustering, global, and local efficiency between frontal and occipital nodes predicted higher acceptance rates in the Ultimatum Game; weaker, less integrated networks predicted costly rejections of unfair offers. A regression model using these resting network properties predicted acceptance with r = 0.58 (leave-one-out) and RMSE ≈ 10.24%. Converging results from a simple choice paradigm show that greater overall alpha/gamma connectivity, higher clustering, and longer path length at rest predict slower Go responses.

In contrast, more small-world-efficient networks predict faster responses, underscoring that topology outperforms band-power summaries for latency prediction. Beyond oscillations, the non-rhythmic background matters: a steeper aperiodic slope (less neural noise) is associated with faster reaction times across difficulty levels, tying the 1/f scaffold to decisional speed. In auditory choice settings, larger delta-1 amplitude at rest also predicts shorter reaction times, linking very slow activity to rapid responding in simple Go/No-Go decisions.

Choice policy under uncertainty and change is most clearly indexed by the balance of slow to fast activity and high-beta rigidity. Across three studies, a higher frontal theta/beta ratio predicted poorer reversal learning and more disadvantageous, reward-driven selections in the Iowa Gambling Task; participants with elevated ratios persisted in risky decks when contingencies became unfavorable, whereas those with lower ratios showed better adaptation and cue-guided orienting efficiency. Where decomposed, this liability was explicitly driven by higher theta rather than lower beta power. Complementing the ratio effects, beta-2 (22–29 Hz) power at rest was a reproducible negative marker of decisional performance: individuals with higher global beta-2 showed longer visual-search reaction times and lower shooting accuracy, together with stronger intrinsic fronto-parietal and fronto-occipital connectivity that reconfigured less with task demands—i.e., a rigid high-beta network profile associated with slower, less precise choices across two sessions and post-training retest. These findings dovetail with topology results showing that globally stronger high-frequency synchronization, coupled with higher clustering and longer paths, is behaviorally sluggish, whereas efficient integration supports speed and consistency.

Boundary conditions are clear. Decision-related resting markers are task-family specific: slow-band power mapped onto recognition drift but not one-back working memory in the same cohort; ratio markers generalized across reversal learning and value-based gambling but speak less to pure perceptual speed; and topology measured with eyes closed was primarily diagnostic for social–economic choice. Taken together, the most precise synthesis is that efficient alpha-band fronto-occipital networks and a steeper aperiodic slope predispose to faster, more rational acceptance or response selection, while elevated theta/beta balance biases toward immediate reward and impairs adaptation when contingencies change; superimposed beta-2 rigidity marks slower, less accurate choices and limited network reconfiguration when demands shift.

### 4.5. Cognitive Flexibility

Cognitive flexibility—adapting task rules, reconfiguring sets, and balancing proactive maintenance with reactive reconfiguration—shows a coherent resting-EEG signature when lateralization, rhythm pace versus power, and coupling topology are considered together. The most specific baseline marker is a lateralized fast-to-alpha balance in mid-prefrontal cortex: greater left-lateral β/α activity predicts smaller switching costs (more efficient phasic set-shifting), whereas greater right-lateral β/α predicts smaller mixing costs (stronger sustained set maintenance) across verbal, spatial, and nonverbal switching paradigms, with additional contributions from right-lateral orbital gyri and pre-SMA for sustained control. A closely aligned interference component is captured by leftward prefrontal β/α at rest, which relates to smaller Stroop costs in both verbal and spatial variants, i.e., better resistance to goal-irrelevant information that otherwise hinders flexible updating.

Across broader samples, alpha phase dynamics, rather than alpha amplitude per se, appear to track interference resolution and flexible control. In particular, a faster individual alpha peak frequency (IAF) is associated with better Stroop interference resolution, with effects most consistently observed in right frontal, bilateral parietal and temporal, and right cingulate regions. Age shows a negative, whereas education shows a positive association with this flexibility factor, underscoring the combined contributions of neurobiological maturation/decline and cognitive reserve. By contrast, alpha power exhibits more task- and site-specific relationships. Higher resting-state lower-alpha (α1) power over parietal regions predicts better performance on the Wisconsin Card Sorting Test, consistent with a baseline neural state that facilitates efficient rule re-engagement and reduced perseveration during set shifting.

Coupling topology refines these links. Within frontal networks, slow-band organization can aid set reconfiguration: higher frontal delta coherence at rest is associated with faster Trails B completion (better switching), whereas posterior high-frequency over-synchrony is counterproductive—stronger posterior gamma coherence relates to poorer sequencing/manipulation and aligns with over-stabilized sets that resist change. Age moderates slow-band interpretations: in older adults, higher central delta power correlates with better Trails B performance (a likely compensatory recruitment), an association absent in young adults. Complementing coherence results, a nonverbal analog of Trails (the Figure TMT) links flexibility to focal fast activity: greater right-frontal high-beta magnitude predicts shorter completion times on the most shifting-demanding part, localizing a beneficial high-beta signature to right frontal control regions for visuospatial set-shifting.

Resting markers also differentiate control mode. In a cued flanker paradigm, higher alpha power and larger 1/f offset at rest are tied to stronger proactive control (using cues to prepare for conflict). In contrast, higher theta relates to larger congruency effects—poorer reactive control—independent of age group. Thus, posterior alpha/aperiodic profiles favor preparatory, goal-maintenance aspects of flexibility, while elevated theta biases toward heavier, less efficient on-the-fly resolution. Outside classic set-shifting, probabilistic reversal—another form of flexibility—depends on slow/fast balance: a higher midfrontal theta/beta ratio reliably predicts poorer adaptation when reinforcement contingencies invert, indicating a baseline tilt toward immediate reward and stickier response policies that impede rule change.

Not all resting measures add leverage. Canonical microstate metrics show little or no association with composite executive/flexibility performance in healthy young adults, even with extensive task batteries, where microstates correlate, effects concentrate on fluid intelligence rather than set shifting per se. By contrast, reserve consistently modulates the resting-state substrate of flexibility: higher cognitive or social reserve is associated with stronger low-alpha long-range connectivity and greater eyes-open/eyes-closed modulation, alongside better strategy use in spatial working memory, a constellation likely to support efficient set reconfiguration despite similar mean performance in some cohorts.

In synthesis, flexible control is foreshadowed by (i) left-lateral prefrontal β/α (phasic switching) versus right-lateral β/α (sustained set maintenance), (ii) faster alpha pace in fronto-parietal hubs (stronger interference resolution), and (iii) supportive slow-band frontal coherence with restrained posterior high-frequency synchrony. Vulnerability to inflexibility is signaled by elevated theta/beta ratios (reversal learning deficits) and over-synchronized posterior fast activity that “locks in” current sets. With aging, selective increases in central delta can compensate for switching, and education/reserve tilt the resting architecture toward more adaptive, proactively prepared control.

### 4.6. Sustained Attention

Sustained attention—keeping a stable task set over minutes while resisting drifts and lapses—shows a precise resting-EEG profile once rhythm pace, spectral geography, and network topology are distinguished. In aging, the single strongest baseline marker is the individual alpha frequency (IAF), not alpha amplitude: faster IAF predicts superior discrimination on the Sustained Attention to Response Task (SART) and statistically mediates the age → performance decline, whereas neither aperiodic exponent/offset nor aperiodic-adjusted alpha power adds explanatory value after correction. Thus, vigilance sensitivity in older adults tracks how fast the alpha “clock” runs at rest.

Spectral power topography differentiates speed from stability during long vigilance. Before a 105 min SART, higher parietal/occipital alpha power predicts slower, but more consistent, responding (a conservative, steady state). In contrast, elevated upper-beta (24–28 Hz) and gamma (28–48 Hz) over left central/temporal sites predict slower and more variable vigilance, characteristic of lapses and recovery cycles; conversely, greater midline parietal gamma relates to faster, more stable performance. Frontal/central alpha tends to forecast greater variability, underscoring that identical bands carry different meanings by scalp region. These patterns echo a general topological constraint: at rest, networks with shorter path lengths and stronger small-world efficiency support faster simple reactions, whereas globally heightened alpha/gamma synchronization with high clustering and long paths yields slower responses—an architecture that burdens rapid monitoring.

Resting baselines also bias attentional style. Individuals with higher posterior alpha power favor broader, global processing. In contrast, higher right parietal/occipital beta aligns with narrower, local focus—enduring preferences that shape how vigilance is expressed (wide monitoring vs. detail tracking). In cueing paradigms, a lower theta/beta ratio at frontal/parietal sites predicts more efficient orienting, and more substantial parietal delta–beta coupling aligns with better self-reported attentional control, even though these EEG metrics do not explain executive interference per se.

Contextual moderators are robust. Under evaluative stress, the frontal theta/beta ratio prospectively identifies who will lose attentional control: higher ratios predict markedly larger post-stressor declines, whereas lower ratios confer resilience. This effect outperforms trait self-reports and localizes to frontal leads. Life-experience factors shift the baseline as well. Higher cognitive/social risk is associated with stronger low-alpha long-range connectivity, modulation of greater eye-openness/eye-state regulation. These patterns correlate with better sustained-attention indices (e.g., higher RVP sensitivity), consistent with more effective state gating and resource deployment. Conversely, social vulnerability is marked by elevated resting delta/theta connectivity and poorer executive screening performance, indicating a slow-band hyper-synchrony that undermines sustained control; education attenuates these effects.

Taken together, sustained attention is strongest when the resting system combines a fast alpha pace, economical (small-world) integration without global over-synchrony, and a posterior distribution of high-frequency support (midline parietal gamma), while avoiding lateral temporal high-frequency overdrive and diffuse slow-band hyper-connectivity. Elevated theta/beta ratios index vulnerability—to contingency changes in attention and to stress—whereas reserve-related low-alpha integration and robust eyes-open/eyes-closed modulation appears protective. These effects are anatomically specific, frequency-specific, and—critically—more dependent on rhythm timing and network organization than on raw band power alone.

### 4.7. Discussion of Development and Aging Moderators

Findings portray a lifespan-sensitive EEG “operating regime” in which two ingredients—oscillatory tempo (especially individual/peak alpha frequency) and the aperiodic background—shift with maturation and aging, reweighting what the same scalp ratios mean at different ages. Normative work shows that alpha frequency accelerates through childhood and peaks in the late teens/twenties before decelerating in mid–older adulthood; alpha power also shows age trends once the 1/f background is accounted for. These developmental arcs are consistent with thalamo-cortical maturation in youth and tempo slowing later in life, offering a principled basis for the age-contingent sign flips observed for theta/alpha-style ratios.

Age-graded dissociations also fit with evidence that EEG power spectra mix periodic peaks with an aperiodic component whose slope and offset change markedly across the lifespan. Parameterization methods (e.g., FOOOF/specparam) demonstrate that developmental increases in alpha tempo and infant beta peaks coexist with steep, high-offset spectra early in life. In contrast, adulthood and aging typically bring a flatter aperiodic slope and lower offset—changes can masquerade as “oscillatory” differences if not modeled explicitly. This helps explain why the same band-ratio can index efficient control in children but something closer to slowing in older adults: the denominator and the background both move with age.

At the level of large-scale state dynamics, canonical microstate norms across ages show robust shifts in mean duration, occurrence, and transition structure from school age to late life. These parameters change strongly with age, even when behavior is held constant, which supports the conclusion that microstate alterations are largely age-dominant traits in healthy cohorts, with task read-outs emerging only in specific contexts. Methodological updates further caution that “canonical four” templates hide notable topographic variability across studies—another reason to interpret age effects on microstates as reorganizations of global states rather than one-to-one markers of specific cognitive processes.

Results in older adults—where alpha tempo captures variance in vigilance and sustained attention while some aperiodic links attenuate—align with recent source-level and scalp reports of age-related alpha slowing and reduced posterior alpha power during rest. These rhythmic changes, layered on a flatter aperiodic background, provide a physiological rationale for why inhibitory control and vigilance become increasingly constrained by timing rather than broad 1/f shifts in later life.

Finally, the moderating role of cognitive is well-grounded. CR is classically defined as the capacity to maintain performance despite brain aging or pathology through more efficient, higher-capacity, and more flexible network recruitment, shaped by lifetime exposures such as education, occupational complexity, and enriched activities. Contemporary frameworks and consensus statements emphasize that CR is distinct from brain reserve/maintenance and is best operationalized as better-than-expected performance given brain status, with neural implementations observed as reduced processing cost and task-dependent reconfiguration. EEG patterns that differ by education/experience (e.g., alpha-theta connectivity gating across eyes-open/closed) are therefore consistent with CR acting as a set-point shifter: high-CR individuals operate at faster temporal regimes and exhibit stronger state-dependent modulation, which can blunt or invert aging-typical EEG–behavior couplings without requiring superior test scores at baseline.

Methodologically, these patterns argue for (i) explicit periodic/aperiodic separation when comparing age groups; (ii) treating IAF/PAF as a covariate or matching variable, especially in vigilance and inhibitory paradigms; (iii) caution with band ratios (their numerator, denominator, and background all age); and (iv) modeling CR proxies (education, literacy/IQ, occupational complexity, lifestyle) as moderators rather than nuisance covariates. Conceptually, age-dependent sign reversals and reserve effects are precisely what lifespan electrophysiology predicts: the brain’s resting “set-up” changes over development and aging, and CR shifts that set-up again—so the same spectral metric need not carry the same cognitive meaning across groups.

## 5. Proposed Mechanisms of Executive Function Performance Based on Resting-State EEG

Explaining the mechanisms and neural correlations based on EEG is difficult. Examining the EEG and correlating it with executive function results often tells us little about the neural key that drives cognitive parameters. Deciphering the relevant neural behavior requires extensive neurophysiological research not directly related to cognitive functions or EEG. The following attempts are made to establish the neurophysiological basis of executive function performance.

### 5.1. Alpha: Inhibitory Gating and Temporal Sampling

Across the EF domains we reviewed, the most coherent mechanistic through-line for alpha (~8–13 Hz) is twofold: (i) gating by inhibition—alpha implements top-down, spatially and feature-specific suppression of task-irrelevant circuits; and (ii) temporal sampling—the intrinsic pace of alpha sets the size of perceptual/attentional “windows,” shaping how quickly and cleanly information is segregated, selected, and updated. Below, we synthesize convergent evidence from task-based EEG/MEG, invasive recordings, causal simulation, computational work, and clinical paradigms (avoiding resting-EEG correlates) to anchor the EF–alpha links observed in our results.

#### 5.1.1. Gating by Inhibition: What Alpha Does, Where, and How

An extensive task literature supports the “gating-by-inhibition” account: alpha power rises in cortical regions that should be suppressed and falls where processing must be enhanced—a proactive, selective mechanism seen across vision, audition, and touch. Conceptually, strong alpha imposes phasic (cycle-locked) inhibitory pulses that reduce local excitability and routing along task-irrelevant pathways [137,138]. Classic spatial-attention paradigms show anticipatory parietal–occipital alpha lateralization: alpha increases ipsilaterally to the to-be-ignored hemifield (suppression) and decreases contralaterally to the attended hemifield (facilitation), with the magnitude of this lateralization predicting distractor resistance. These effects generalize across modalities and tasks and have been replicated with source-resolved MEG/EEG and intracranial measures [137,139,140]. Brief rhythmic brain stimulation at participants’ alpha frequency (tACS) biases perception and attention—speeding or slowing temporal segregation and shifting detection thresholds—consistent with direct entrainment of inhibitory sampling. TMS/EEG studies likewise show that momentary prestimulus alpha states (phase/power) in early visual cortex predict whether stimulation elicits a percept or is quenched, indexing rapid alpha-gated excitability changes [141,142]. At the network level, fronto-parietal control regions sculpt posterior alpha: the microstate-level stage-setting is less informative than directed, frequency-specific control. For example, frontal eye fields and dorsal attention nodes causally modulate occipitoparietal alpha. Thereby, in humans and monkeys, alpha/beta rhythms preferentially carry feedback (top-down) influences while gamma/theta carry feedforward signals—exactly the division one expects if alpha implements selective suppression and predictive routing [143,144]. Newer work shows alpha travels as propagating waves with distinct directions for top-down versus bottom-up control during attention, refining the simple “static gain” view into a spatiotemporal control signal that sweeps the cortex to bias processing [145]. Crucially, alpha power/phase gates not just mean firing, but what other rhythms can do. Layer-resolved recordings in macaque V1 show that alpha phase modulates gamma power and spiking, establishing pulsed windows for local computation and interareal communication—an implementation detail with direct implications for EF speed/accuracy trade-offs [146].

Multiple lines of evidence point to thalamo-cortical drivers and GABAergic circuitry. Animal laminar recordings locate alpha generators and show negative alpha–spiking relations in sensory cortex; computational models reproduce alpha via thalamic high-threshold relay cells and predict its state-dependent routing effects; human MRS and pharmacology link inhibitory tone to the properties of perceptually relevant rhythms. These findings situate alpha at the intersection of thalamic pacemaking and cortical interneuron networks [147,148,149,150].

If alpha implements pulsed inhibition, its frequency should bound the temporal resolution of selection. That prediction is borne out: individual alpha frequency (IAF) correlates with the fineness of temporal discrimination (e.g., two-flash fusion thresholds), and meta-analytic estimates indicate medium-to-large associations between IAF and a range of temporal-processing measures. Driving alpha with tACS shifts these windows causally (faster entrainment → better segregation; slower → more integration). These effects provide a principled bridge from a resting trait (IAF) to online EF demands that hinge on fast sampling and conflict-free updating [151,152,153].

When tasks demand suppressing actions or distractors, larger anticipatory alpha over irrelevant representations predicts fewer false alarms and smaller Stroop/Flanker costs; conversely, failure to up-regulate alpha where needed leads to distractor leakage. Clinical paradigms highlight the exact mechanism: people with schizophrenia show reduced alpha event-related desynchronization (ERD) during basic visual processing—consistent with impaired downregulation of task-irrelevant cortex—while attention-alignment deficits track abnormal modulation of low-frequency rhythms [154].

#### 5.1.2. Clinical and Systems Contexts

During interference and WM tasks, individuals on the Alzheimer’s pathway exhibit abnormal alpha ERD (less task-appropriate desynchronization) and altered alpha-band connectivity under load—physiological signatures that co-vary with medial temporal atrophy and predict conversion [155,156,157]. In ADHD, MEG shows diminished alpha (and beta) ERD during somatosensory processing—consistent with deficient inhibitory gating rather than pure sensory deficits [158].

### 5.2. Theta: Control Signals from Medial Frontal Cortex

An extensive task-based literature converges on frontal-midline theta (FMθ; ~4–8 Hz over FCz/dACC–pre-SMA) as a control signal that is up-regulated when behavior must be monitored or adjusted—after errors, under stimulus–response conflict, during rule changes, and when choices are difficult or surprising. In time–frequency analyses, these increases are induced mainly (non-phase-locked to events), distinguishing them from classic ERPs and pointing to oscillatory coordination rather than mere evoked responses. Mechanistically, FMθ scales with the need for control (e.g., conflict and prediction error), predicts single-trial adjustments, and couples the medial frontal “monitor” to lateral prefrontal and subcortical “implementers” [45,159,160].

Source-resolved EEG/MEG and intracranial work localize control-related theta to dorsal ACC and adjacent medial frontal cortex (pre-SMA), which grows with error likelihood, conflict, and negative feedback [161]. These dynamics align with normative accounts—most prominently, the Expected Value of Control (EVC) theory—which posit that ACC evaluates the benefits vs. costs of allocating control and issues signals to recruit it [162]. Theta power and phase track this computation, rising when the EVC of additional control increases (e.g., on incongruent, high-conflict, or surprising trials) [163,164,165].

FMθ appears to coordinate distributed control networks via phase-synchronous coupling. After errors or during conflict, theta phase-synchrony between medial frontal cortex and lateral PFC/FEF increases; the strength of this mPFC ↔ lPFC coupling predicts the size of behavioral adjustments. During instruction implementation, mPFC establishes theta-phase connectivity with posterior task regions, consistent with routing task rules to sensorimotor processors. Critically, the phase of midfrontal theta—more than its amplitude—best organizes downstream activity and reaction-time adjustments [166,167,168].

A tight link between mPFC theta and the subthalamic nucleus (STN) explains how control signals slow or withhold actions under conflict. In patients with STN electrodes, conflict and switching evoke trial-locked increases in STN theta that phase-lock with mPFC; deep-brain stimulation (DBS) that disrupts this coupling reduces the normal, caution-inducing rise in decision threshold. Modeling and intracranial data agree that mPFC → STN theta, along the hyperdirect pathway, transiently raises the decision bound (“hold your horses”) and theta organizes within-trial caution. In contrast, beta dynamics track across-trial adjustments [169,170,171,172].

FMθ often nests faster activity, providing a temporal scaffold (“communication-through-coherence”). Human ECoG/EEG shows theta–gamma phase–amplitude coupling (PAC) during high-control states and memory protection, with gamma amplitude and spiking aligned to theta phase; frontal–hippocampal theta–gamma PAC increases with control demands when multiple items must be protected from interference. In frontal networks, theta phase also coordinates high-gamma information encoding across regions. These findings support a division of labor in which theta sets control timing while gamma/beta carry local computations [173,174,175].

During difficult or surprising decisions, FMθ covaries with pupil dilation—a proxy for locus-coeruleus noradrenergic activity. The joint evolution of theta and pupil suggests that medial-frontal control signals track and help regulate arousal, thereby optimizing performance, consistent with adaptive gain accounts [176].

If FMθ carries control commands, exogenous theta should alter control. Indeed, θ-tACS centered over mPFC reduces Stroop/Simon conflict effects and modulates post-error adjustments; phase-specific protocols that increase in-phase theta between mPFC and other nodes produce larger effects than out-of-phase stimulation. Relatedly, invasive theta-burst protocols (TBS) reveal frequency-specific entrainment and short-term plasticity in human frontal circuits, and prefrontal theta–gamma targeted stimulation can reshape or working-memory/control networks measured with EEG/TMS. While not every study finds behavioral benefits (task and montage matter), the overall pattern supports a causal role for theta-mediated coordination [177,178,179,180,181,182,183].

Not all theta increases are “conflict specific.” Some studies argue that midfrontal theta partly reflects time-on-task or generic difficulty; however, removing phase-locked components leaves robust conflict-related theta, and theta phase (not just power) predicts trial-wise adjustments—evidence against a purely nonspecific account. A balanced view is that FMθ indexes the need for and deployment of control, rising with conflict, surprise, and difficulty insofar as these raise EVC; its functional impact depends on where it synchronizes (lPFC vs. STN vs. posterior cortex) and on phase-specific coupling [159,165,184].

Although our review above concerned resting correlations, these task-based mechanistic data clarify why trait differences in theta-related systems can predict executive outcomes at rest. Individuals whose medial frontal circuits (i) more effectively generate FMθ bursts, (ii) phase-synchronize with lateral PFC and STN when needed, and (iii) flexibly couple theta with higher-frequency activity should exhibit better manipulation-heavy working memory, interference control, and adaptive decision policies—whereas blunted, mistimed, or hyper-reactive theta signals (as seen in ADHD and OCD, respectively) would tilt the speed–stability and caution–impulsivity trade-offs in opposite directions.

#### Clinical Signatures and Pathophysiology

Children/adolescents with ADHD show attenuated error-related FMθ and poorer post-error adjustments; twin-genetic work links variability in theta signaling to ADHD risk, suggesting that weakened medial-frontal control bursts may underlie reactive-control deficits [185,186]. By contrast, OCD and anxiety often show enhanced error-related theta/ERN, consistent with hyperactive performance monitoring; time-frequency work confirms larger error-theta in OCD. These alterations map onto symptom severity and may index risk for internalizing pathology [187,188]. Altered midfrontal error/theta signals also appear in major depression and across diagnoses, supporting FMθ as a transdiagnostic marker of control function (with directionality depending on phenotype: blunted in externalizing; heightened in internalizing) [189,190].

### 5.3. Beta: Maintaining the “Status Quo” vs. Releasing It

An extensive task-based literature—spanning human/animal M/EEG, intracranial recordings, stimulation, and clinical electrophysiology—supports a functional role for beta-band activity (~13–30 Hz) in stabilizing ongoing sensorimotor and cognitive sets (“status quo”) and, conversely, shows that down-regulating or temporally interrupting beta facilitates change (movement initiation, set updating, stopping) [46,191,192,193]. In motor systems, elevated beta accompanies tonic postural control and resistance to change, whereas beta suppression precedes movement and beta “rebound” follows it; in cognitive control networks, brief beta bursts over right inferior frontal/prefrontal cortex and the subthalamic nucleus (STN) mark moments of motor suppression and across-trial rule/set adjustments [172,194,195,196,197,198,199,200,201,202,203]. Pathological exaggeration of beta—exceptionally prolonged STN beta bursts in Parkinson’s disease (PD)—impairs flexibility and speed, and targeted suppression restores function [204]. Together, these data operationalize beta as a stabilizer that can be adaptively increased to hold the current state or decreased/interrupted to permit change.

In healthy adults, beta power over primary sensorimotor cortex (M1/S1) decreases before and during movement (movement-related beta desynchronization, MRBD) and then increases after movement (post-movement beta rebound, PMBR) [205,206]. PMBR scales with the evaluation of movement error/uncertainty, “resetting” the system to a default, stable state, consistent with the status quo account [207,208]. Pharmacological and MRS studies link these dynamics to GABAergic inhibition: benzodiazepine-like GABA_A modulators reliably elevate beta, and M1 beta measures (including PMBR) covary with cortical GABA levels and GABA-dependent plasticity [209,210,211]. Causally, entraining the cortex at ~20 Hz with tACS slows voluntary movement [191]. Mechanistically, single-trial work shows that transient beta bursts—rather than sustained oscillations—dominate sensorimotor beta; bursts just before imperative cues predict slower responses, and laminar/biophysical evidence ties bursts to coincident synaptic drives in deep/superficial layers [192,212,213,214].

In the corticobasal ganglia network, the STN and cortex exhibit coherent beta increases when actions are held or must be canceled [203,215]. Parallel evidence from animal models of dopaminergic dysregulation converges on the same principle: dopamine tone shapes beta-band synchrony and its behavioral consequences across corticobasal ganglia loops. In Parkinsonian models, dopamine depletion is consistently accompanied by exaggerated beta synchronization/bursting within basal ganglia circuits, and experimental manipulations of dopamine tone can shift beta characteristics (including frequency and coherence) across the cortex and basal ganglia, supporting a causal neuromodulatory lever on “status quo” dynamics [216,217,218]. Consistent with our interpretation that flexible behavior depends on appropriately state-dependent coupling rather than rigid synchrony, recent work in a hyperdopaminergic genetic model (DAT-KO rats) shows behavior-linked alterations in striatal–prefrontal LFP organization, including reduced cross-structure synchrony during active exploration and more uniform (less behavior-differentiated) patterns overall—features interpreted as reduced striatal flexibility in adapting motor acts [219]. In PD, beta is not tonically high but occurs in bursts whose longer duration predicts bradykinesia and rigidity [220]; dopaminergic therapy shortens these bursts and improves motor signs [204]. Adaptive deep brain stimulation (aDBS) that selectively truncates long STN beta bursts outperforms continuous DBS in pilot/early clinical studies, directly linking pathologic beta dynamics to behavior and treatment [221]. Conceptually, aDBS treats beta activity as a control signal rather than a purely descriptive biomarker. Stimulation is delivered only when local field potential (LFP) beta power exceeds a predefined threshold or when beta bursts become abnormally prolonged, with the explicit aim of disrupting pathological synchrony while avoiding unnecessary stimulation. This closed-loop architecture strengthens causal inference by directly linking beta suppression to improvements in motor function. Compared with continuous DBS, aDBS may reduce stimulation-related side effects and energy consumption by selectively targeting pathological beta states rather than applying indiscriminate, constant stimulation [215].

### 5.4. Network Topology: Integrated Yet Not Over-Synchronized

A central lesson from network neuroscience is that executive function thrives when large-scale brain networks strike a balance between segregation and integration—organized into specialized modules that can rapidly inter-communicate via connector hubs and transiently integrated “global” states—rather than when activity is either fragmented or globally locked into high synchrony. Graph theory gives this idea teeth: a short characteristic path length/high global efficiency index indicates ease of information flow; high clustering/modularity portrays local specialization; and the participation coefficient identifies connector hubs that bridge modules. Small-world architectures (high clustering with short paths) implement this balance efficiently and appear across modalities and species [53,222,223].

Time-resolved fMRI shows the brain shuttles between segregated and integrated network states during demanding cognition; integrated states recruit hubs, are accompanied by larger pupil diameter (arousal), and enable faster, more accurate performance. Pharmacologic elevation of catecholamines (atomoxetine) causally increases network integration and flexibility in task contexts, reinforcing a neuromodulatory lever for shifting the brain into integration-ready regimes. Complementary work argues that frontoparietal “flexible hubs” (high participation coefficient; globally variable connectivity) reconfigure their coupling across tasks to implement control. Together, this literature grounds a mechanistic link from transient integration → efficient control and EF [224,225,226].

Information processing suffers when global synchrony is excessive. Theory and experiment converge on near-critical dynamics—neither too ordered (rigid synchrony) nor too disordered (fragmentation)—as optimal for dynamic range, information transmission/capacity, and rapid switching among network states (metastability). Whole-brain modeling and MEG/EEG analyses identify resting dynamics near maximal metastability; cortex appears homeostatically tuned near criticality, and proximity to this regime tracks task performance. These results formalize the intuition that over-synchronization collapses the brain’s dynamic repertoire, while moderate, task-tuned coupling supports control [227,228,229,230].

Several architectural motifs repeatedly emerge as pro-EF.

High-degree, high-participation hubs (notably in frontoparietal and thalamic territories) coordinate cross-module traffic. A structural “rich club” of densely interlinked hubs forms a high-capacity backbone for long-range communication; lesion and modeling work show that perturbing rich-club links disproportionately degrades global communication. These hubs’ centrality also explains their vulnerability across disorders [231,232,233,234,235].

During learning and task switching, networks reconfigure modules and hub affiliations; individuals who reconfigure more adaptively learn faster and exert control more effectively. Neuromodulatory gain changes (linked to locus coeruleus arousal and pupil dilation) promote transitions into integrated states with hub recruitment, thereby tightening the link between arousal systems and topology [236,237,238].

Across modalities and methods, EF is best supported when (i) connector hubs (frontoparietal, cingulo-opercular, thalamic) can flexibly up-weight inter-module links (high participation) to meet control demands; (ii) the system can enter integrated states transiently—often under catecholaminergic gain—without falling into rigid global synchrony; and (iii) the structural scaffold (including the rich club) affords short effective paths. Too little coupling (fragmentation) or too much (hypersynchrony) harms EF by preventing coordination and collapsing the brain’s dynamic repertoire. This “integrated-yet-not-over-synchronized” regime is precisely the operational sweet spot predicted by small-world theory and critical brain dynamics.

## 6. Resting-State EEG: In Search of a Biomarker of Improved Neuroplasticity

Here, “improved neuroplasticity” means a brain state that learns or adapts more rapidly, flexibly reconfigures networks to meet demands, and resists age- or stress-related losses in executive control. Across the literature, a small set of resting-EEG features consistently tracks that profile more robustly than any single band-power metric. Foremost is faster alpha pace (IAF/PAF), especially frontally, which predicts manipulation-heavy working memory and interference-laden control, mediates the age-related vigilance decline, and even shows same-day state sensitivity. In healthy aging, less excess slow power—lower frontal theta/delta—co-occurs with stronger executive profiles and with higher reserve or more active lifestyles, whereas in development, the sign flips, and greater frontal theta supports working memory. Aperiodic (1/f) structure behaves as a domain-general efficiency/noise index: steeper slopes and higher offsets often align with faster decisions and better executive composites, and, longitudinally, the match between IAF and the exponent matters most—mismatched profiles forecast steeper decline, with education/reserve moderating both strength and direction. Connectivity and topology repeatedly outperform raw power: shorter paths, higher global/local efficiency, flexible alpha-band reconfiguration, and strong state gating (larger EO ↔ EC modulation) mark better working memory, inhibition, decision speed, and vigilance, whereas global over-synchronization—especially fronto-posterior theta or broadly strong alpha/gamma—slows responses, impairs manipulation, and reduces adaptability. Temporal organization carries parallel information: lower long-range temporal correlations (DFA/LRTC) in delta/theta are associated with higher working-memory capacity independent of power. Stable lateralization of fast-to-alpha power over the prefrontal cortex further biases control mode; left-lateralized β/α supports agile, interference-resistant switching, while right-lateralization favors sustained goal maintenance. By contrast, band ratios (e.g., theta/beta) are highly context-dependent and often null in homogeneous young samples; microstate metrics track age reliably but relate weakly to executive outcomes outside aging cohorts, and are best treated as secondary markers.

Because plasticity is multivariate, a composite outperforms any lone feature. A practical “Neuroplasticity Readiness Index” can be formed by age- and montage-normed z-scores that weight: higher IAF/PAF (frontally), an IAF–exponent match with offset interpreted in light of education/reserve, age-appropriate slow-band tone (lower frontal theta/delta in elders; neutral/positive theta in youth), graph efficiency and shorter paths with reduced global alpha/gamma synchronization, lower fronto-posterior theta coherence but stronger within-frontal beta/gamma coherence for simple span (while avoiding rigid motor-beta networks linked to worse stopping), lower slow-band DFA/LRTC with higher alpha-network flexibility, left-lateralized prefrontal β/α, and larger EO ↔ EC modulation of alpha/theta connectivity.

For biomarker deployment, recordings should include ≥5–6 min eyes-closed (plus eyes-open), ≥32 channels, vigilant artifact/drowsiness control, and explicit decomposition of periodic vs. aperiodic components before inference; report both absolute/relative power, fixed band edges, and individual-frequency measures. Connectivity should be estimated with lag-insensitive metrics and summarized with graph efficiency/path length and alpha-network temporal variability. Validation must separate speed from accuracy and dismaintenance, manipulation, and spatial updating, because low-load or ceiling tasks under-reveal EEG-behavior links. Moderators—age, education/reserve, lifestyle, SES—should be modeled explicitly, given repeated sign flips and gating effects. Trait-leaning measures such as IAF, aperiodic slope, and graph efficiency are well-suited for baseline stratification. In contrast, PAF shifts, EO ↔ EC modulation, alpha-network flexibility, and LRTC are functional state-sensitive endpoints for change detection.

Against that backdrop, plausible evidence of improved neuroplasticity over weeks to months (training, activity, neuromodulation) would include: upward shifts in IAF/PAF (especially frontal) with parallel gains in manipulation-heavy working memory or interference control; increases in network efficiency and alpha-network flexibility with reductions in global over-synchronization (notably fronto-posterior theta) alongside faster and more consistent performance; reductions in delta/theta DFA/LRTC with working-memory accuracy gains; movement toward a matched IAF–exponent profile and stabilization of that match; age-appropriate slow-power changes (e.g., reduced frontal theta/delta in elders without expecting the same in children); stronger EO ↔ EC modulation of alpha/theta connectivity tracking better vigilance and strategy use; and, for interference-prone flexibility, a shift toward left-lateralized prefrontal β/α with smaller switching/Stroop costs.

Important limitations remain. Several markers invert across development/age or with reserve (notably theta and the exponent), task dependence is substantial (nulls are expected with low load or homogeneous samples), ratios are unreliable as global indicators, and strongly synchronized motor-beta networks can predict worse stopping despite generic “beta up” heuristics. The bottom line is that a credible resting-EEG biomarker of improved neuroplasticity is not a single frequency band, but a multi-feature signature emphasizing faster alpha pace; efficient, flexibly reconfigurable networks with modest global synchronization; optimized temporal organization of slow activity; and an IAF–aperiodic match—always interpreted through the lenses of age and reserve. This composite aligns closely with who learns faster, reconfigures sets more readily, sustains attention more stably, and resists interference across the assembled studies.

## 7. Limitations and Future Directions

### 7.1. Study Design and Sampling

Most studies are cross-sectional and underpowered, limiting causal inference and generalizability. Several samples are small or convenient cohorts of students, while others focus primarily on healthy older adults with relatively high educational levels. Socioeconomic and cultural diversity is uneven; when SES or social vulnerability is measured, it is associated with both EEG and executive outcomes. Longitudinal work is rare and sometimes hampered by attrition or limited follow-up power.

### 7.2. Construct Coverage and Task Impurity

EF was indexed with heterogeneous tasks: working memory (digit span, n-back), inhibition (Go/No-Go, SST, antisaccade, Stroroop), switching (TMT-B, flanker), sustained attention (SART, RVP), and reasoning (Raven). Many studies relied on single tasks per domain, susceptible to task-specific variance and strategic differences. Where broader batteries or latent factors were used, EEG–EF links were often weaker or null. Diffusion-model parameters were informative in a few cases but were seldom used elsewhere.

### 7.3. Heterogeneity in EEG Acquisition and Preprocessing

Protocols varied widely in duration (1–20 min), eyes-closed vs. eyes-open, channel count (14–64+), and artifact handling. Short recordings increase sensitivity to state fluctuations (e.g., drowsiness, mind-wandering); only a few works have directly examined stability over minutes. Definitions of bands differ (e.g., theta 4–6.5 vs. 4–8 Hz; alpha subdivisions; beta up to 30 vs. 28 Hz), as do power metrics (absolute vs. relative, Z-scored vs. raw). Several studies used low-density systems or consumer headsets, complicating source-level inference. Connectivity metrics ranged from coherence to PLI/graph measures, with variable attention to volume-conduction/leakage. Microstate segmentation choices (A–D vs. A–E) and parameterization also differed.

### 7.4. Oscillatory vs. Aperiodic Confounds

Only a subset separated periodic (oscillatory) from aperiodic 1/f activity; without this, apparent “power” effects can reflect background slope/offset differences. Mixed findings around alpha/theta power across age and EF likely partly reflect unmodeled aperiodic variance.

### 7.5. Inconsistent Directionality and Age Interactions

Associations are often bidirectional across studies and are moderated by age and education/cognitive reserve. Examples are as follows:

1. Theta power associatestively assocwithted with EF in some older cohorts but positively in others; theta/alpha ratios predict better memory in youth but worse reasoning in older adults.
2. Alpha: Faster individual alpha frequency (IAF) is associated with better EF/processing speed in several samples, though not all studies replicate this finding.
3. Aperiodic slope/offset links to g, fluency, or EF appear in some datasets but not uniformly. These divergences underscore nonlinearities in development, cohort differences, and analytic variability.

### 7.6. Statistical Practices

Multiple comparisons (many bands × regions × metrics) with limited correction in some reports are common. Median splits and post hoc subgrouping appear in places. Machine-learning models are often validated solely through internal cross-validation, risking optimistic performance estimates. Incremental validity beyond demographics/education and baseline cognition is not consistently tested.

### 7.7. Ecological and Clinical Validity

While some studies use real-world-relevant outcomes (e.g., allocentric navigation, IPA, social vulnerability), most rely on laboratory tasks with uncertain ecological generalizability. Clinical translation remains preliminary: predictive value beyond brief cognitive screens is modest, and normative thresholds have not been established.

### 7.8. Future Directions

A pragmatic near-term priority ladder.

The agenda outlined below spans large-scale consortia, mechanistic modeling, and clinical translation. However, meaningful progress does not depend on “utopian” resources. We therefore delineate a minimal set of near-term priorities that most laboratories can realistically implement within the next few years, and that would substantially improve cross-study comparability, mechanistic interpretability, and cumulative knowledge, even in single-site investigations:

(1) Mandatory periodic-aperiodic spectral separation in all spectral analyses, with reporting of both oscillatory peak-centered power and aperiodic (1/f) parameters, as many apparent band-power effects are confounded by slope and offset.
(2) Latent-variable measurement of executive functions, using at least two tasks per EF domain or a brief multi-domain battery, to reduce task impurity and stabilize brain-behavior associations.
(3) Outcome framing that dissociates speed from accuracy, for example, via diffusion-model parameters or robust reaction-time distribution metrics, and that explicitly distinguishes working-memory maintenance from manipulation, given their domain-specific rsEEG relationships.
(4) Reproducible “minimum viable” acquisition, quality control, and reporting standards, including at least 5–6 min each of eyes-closed and eyes-open resting EEG, explicit monitoring and control of vigilance or drowsiness, and open, preregistered preprocessing and feature-extraction pipelines.

Collectively, these steps define a feasible baseline standard that can be adopted immediately and that would meaningfully increase the yield, robustness, and replicability of rsEEG-EF findings, irrespective of whether large multi-site cohorts are available.

#### 7.8.1. Harmonized, Adequately Powered, and Longitudinal Designs

To build a shared, reproducible framework that can (i) detect small but reliable EEG–EF associations, (ii) map age × EEG interactions across the lifespan, and (iii) separate trait from state effects, we recommend planning for small brain–behavior links (|r| ≈ 0.10–0.20) after multiple-comparisons control and powering accordingly: a multi-site discovery cohort of N ≈ 800–1200 balanced across age bins provides ≥80–90% power for |r| ≈ 0.12–0.15 with FDR control, paired with an independent replication cohort of N ≈ 300–500 using an identical protocol; for longitudinal change, oversample by +25–30% to offset attrition and retain ≥80% power for change–change effects. Sampling should span adolescents (12–17), young adults (18–35), midlife (36–59), and older adults (60–80+), with n ≥ 150 per band, stratified by sex, education (≤12/13–16/>16 years), and SES quartiles, with language background recorded; include only participants with normal/corrected vision and hearing, no major neuro/psychiatric illness, and log stable medications (e.g., stimulants, sedatives) plus caffeine/nicotine/sleep in the prior 24 h. To disentangle trait from state, use a measurement schedule with a baseline wave (T0) and follow-ups at 12 months (T1) and 24 months (T2), augmented by a “burst” of three short re-tests in weeks 2–4 after T0 to estimate within-person reliability and state variance, with pre-session controls for sleep (actigraphy or diary), recent physical activity, and mood/stress. EEG acquisition should be harmonized: ≥64 channels on a 10–10 montage, ≥500 Hz sampling (preferably 1 kHz), identical amplifiers/caps across sites or traveling-heads calibration; record both eyes-closed (8 min) and eyes-open fixation (8 min) in 1 min sub-epochs to monitor drift; include vigilance control (EOG, EMG, and pupillometry or an embedded psychomotor vigilance test), excluding drowsy epochs by predefined rules (alpha attenuation plus elevated delta). Enforce impedance < 10 kΩ, channel loss < 10%, artifact rejection < 25% of total time, and site-level QC dashboards; preprocessing must follow a preregistered EEG-BIDS pipeline (bad-channel interpolation, ICA/ASR, average reference) with public code and parameters. Feature definition must be comparable across sites and waves: separate periodic (band-limited power) from aperiodic 1/f components (exponent, offset) with fit diagnostics; define bands as delta 1–3 Hz, theta 4–7 Hz, alpha split into α1 8–10 Hz and α2 10–12 Hz, beta 13–30 Hz, gamma 30–45 Hz, and also compute individualized bands anchored to each person’s IAF; estimate connectivity with leakage-robust measures (imaginary coherence or wPLI) in source space (template head model, MRI-based when available); quantify dynamics via microstates (A–E; GEV, duration, occurrence, transitions), long-range temporal correlations (DFA), and graph metrics (efficiency, clustering), and report test–retest reliability (ICC) for every feature. Executive function should be modeled with at least two tasks per domain to reduce task impurity and enable latent variables: inhibition (Stroop and Stop-Signal with SSRT), updating/working memory (2-/3-back and complex span), shifting (number–letter and category-switch), processing speed (Digit–Symbol, TMT-A), and reasoning (Raven/Matrix), using alternate forms across waves and extracting computational readouts (e.g., drift rate, boundary separation, non-decision time from diffusion models; ex-Gaussian RT; lapse rates) to improve mechanistic interpretability. Covariates must include age, sex, education, SES, sleep quantity/quality, medications, mood/anxiety, physical activity (including incidental), and cognitive-reserve indices (education, occupational complexity, vocabulary), plus site covariates (amplifier, cap model, room noise, time of day, experimenter ID); apply ComBat or equivalent harmonization for site effects after QC and verify that biological variance is preserved. The preregistered analysis plan should specify multilevel models with random intercepts for person and site, using EF latent variables as outcomes or predictors of EEG features; model change with latent growth curves and random-intercept cross-lagged panels to separate between- vs. within-person effects and to test age × EEG interactions; control multiplicity via FDR within coherent feature families (periodic, aperiodic, connectivity, dynamics); require internal split-half replication within the discovery sample and external replication in the independent cohort before making claims; quantify incremental validity over demographics, cognitive reserve, and baseline cognition, reporting ΔR^2^, calibration, and decision-curve analysis rather than correlations alone. Reliability targets should be ICC ≥ 0.70 for candidate biomarkers (e.g., power metrics, IAF, aperiodic parameters, SSRT), and deliver age- and education-stratified centiles for key EEG features (IAF, exponent, theta/alpha ratios); embrace open science via preregistration or registered reports, BIDS-formatted raw and preprocessed data, full analysis code, and site QC metrics under a clear data-use agreement. To manage attrition and burden, provide flexible scheduling, reimbursement, brief home check-ins for burst sessions (with validated portable EEG where appropriate), and reminders to achieve ≥80% retention through T2, while keeping per-wave burden ≤ 2.5 h (EEG ≤ 45 min net recording; EF battery ≤ 60–75 min). The tangible deliverable should be a turnkey protocol pack—SOPs, preprocessing code, EF battery scripts, and QC dashboards—that enables any site to join a pooled, continuously growing EEG–EF consortium with compatible data from day one.

#### 7.8.2. Mechanistic Precision: Beyond Band Power

To move from descriptive correlations toward mechanism, analyses must separate oscillatory events from background activity, quantify how rhythms are organized in time, and test how information flows within control networks. First, every spectral result should be decomposed into a periodic (oscillatory) and an aperiodic (1/f) component, because many “power” effects ride on slope/offset changes that reflect excitation–inhibition balance rather than true oscillations. Concretely, fit spectra in 1–45 Hz with a knee-enabled spectral parameterization (e.g., SpecParam/FOOOF; peak-width limits 0.5–12 Hz, max n_peaks 8, goodness-of-fit R^2^ reported per channel) and/or IRASA (non-integer resampling factors 1.10–1.90, step 0.05) to obtain exponent and offset alongside peak-centered power; treat periodic power both raw and aperiodic-adjusted, and reject fits with R^2^ < 0.90 or with residual harmonics that indicate non-sinusoidal contamination. Frequencies should be individualized rather than fixed: compute IAF by center-of-gravity over 8–13 Hz at POz/Oz/PO3/PO4 with automatic QC flags, then define θ as 0.5·IAF–0.2·IAF above and below its nominal range (e.g., θ ≈ IAF–6 to IAF–3 Hz), split α into α1 (IAF–4 to IAF–2) and α2 (IAF–2 to IAF), and set β to 13–30 Hz; report analyses with both individualized and canonical bands to demonstrate robustness.

Second, quantify oscillatory bursts rather than averaging power across minutes. Bandpass each individualized band with zero-phase FIR (transition width 1 Hz; −6 dB at band edges), extract the analytic signal, and detect bursts using cycle-by-cycle criteria (instantaneous amplitude > 2.5 SD above the band’s baseline with a minimum of three contiguous cycles and peak frequency within ±15% of the individualized peak). For each region, compute burst rate (events/s), mean duration (cycles), amplitude, inter-burst interval, and coefficient of variation; report distributions and test–retest ICCs. Mechanistically, frontal-midline θ burst rate and duration are hypothesized trait markers of control signaling, posterior α burst rate and duty cycle index sensory gating, and central β burst persistence indexes motor maintenance/suppression; these hypotheses should be preregistered and linked to EF domains (updating/inhibition/switching) rather than omnibus “EF” scores.

Third, address temporal structure directly. Estimate long-range temporal correlations with DFA on band-limited amplitude envelopes and on broadband residuals (after removing peaks) over window sizes 1–20 s in log-spaced steps; report α_DFA with linearity checks (adj. R^2^ > 0.95 of log–log fit) and exclude segments violating (weak) stationarity. Complemented with wavelet-based Hurst exponents and multiscale entropy (MSE; scales 1–20, SampEn m = 2, r = 0.15·SD). Lower scaling exponents in slow bands have been linked to more flexible (less temporally sticky) dynamics; explicitly test whether lower θ/δ DFA at parietal/posterior sites predicts better updating/working-memory scores, replicating or refuting prior LRTC–WM links. Where possible, compute burst-triggered averages of the aperiodic exponent to test whether bursts transiently “stiffen” 1/f (putative E/I shifts), which would ground trait EF associations in a biophysically interpretable parameter.

Fourth, examine cross-frequency interactions using methods robust to waveform shape. Before coupling, mitigate non-sinusoidal confounds by subtracting the aperiodic fit and rejecting epochs with high harmonic-to-fundamental ratios; quantify phase–amplitude coupling with debiased PAC (dPAC) and cross-bispectrum controls, compute comodulograms for phase ∈ {θ, α} and amplitude ∈ {β, low-γ}, and assess significance with IAAFT surrogates (≥200 shuffles) controlling the family-wise rate via cluster-based permutation. Report whether fronto-midline θ phase modulates parietal β/β/low-γ amplitude at rest, and whether stronger, more focal PAC (smaller spatial extent, higher dPAC z-score) predicts better inhibition or set-shifting; conversely, test whether diffuse, global PAC relates to slower processing (putative inefficiency).

Fifth, move from correlation to directional and network-level descriptions. Compute leakage-robust, frequency-specific directed connectivity (phase-slope index with multitaper spectral estimates, time–bandwidth product 3–4, 2 s windows, 50% overlap) in source space using a standard BEM template when MRI is unavailable; quantify frontal → parietal PSI in θ and α2 and parietal → frontal PSI in β, then summarize with graph measures (global efficiency, participation coefficient) without hard thresholds by integrating across costs or via minimum-spanning trees. Add a dynamic layer using envelope HMMs (6–8 states on band-limited source envelopes; report fractional occupancy, mean lifetime, switching rate) to ask whether higher switching rates among α-suppressed “externally oriented” states and θ-elevated “control” states predict better executive performance. Validate all connectivity with sensor-level controls (imaginary coherence/wPLI) and EMG regressors, because residual EMG can inflate β/γ estimates; reject nodes whose spectrum shows a 30–45 Hz plateau with frontotemporal topography characteristic of muscle.

Sixth, integrate prestimulus–task transfer tests to tie resting markers to known control mechanisms without abandoning the resting paradigm. In a subset of participants, collect a short flanker or stop-signal block and test preregistered mediation: do IAF and α burst duty cycle predict α desynchronization magnitude and SSRT, and does the aperiodic exponent (E/I proxy) explain individual differences in drift rate under conflict? Use multilevel mediation with bootstrap CIs, controlling for age, education, and site; report incremental validity over demographics and cognitive reserve.

Seventh, rigorous artifact and confound control tailored to high-frequency and coupling analyses should be imposed. Include vertical/horizontal EOG, bilateral EMG, and (ideally) mastoid/neck EMG; remove ocular/muscle ICA components with ICLabel probability ≥0.7, regress residual EMG power from β/γ metrics, and perform “eyes-flicker” sensitivity checks to ensure microsaccades do not drive PAC/PSI. Quantify vigilance with pupillometry or an embedded PVT; exclude epochs that meet drowsiness rules (alpha attenuation plus delta surge or pupil constriction) and show that effects hold after excluding the drowsiest quartile.

Eighth, specify statistical and reliability expectations up front. For each mechanistic metric (e.g., θ burst rate, α_DFA, fronto → parietal θ-PSI, θ–β dPAC), report split-half and 2–4-week test–retest (ICC [A,1]); proceed to substantive inference only for ICC ≥ 0.70 or include reliability attenuation corrections. Use multiverse analyses that vary reasonable pipeline choices (e.g., burst thresholds ± 0.5 SD, window sizes for DFA, PSI smoothing ±1 Hz) and display “specification curves” so that claimed effects are not pipeline-specific. Correct multiplicity within each mechanistic family by FDR and present Bayes factors alongside *p*-values to quantify evidence for both effects and nulls. Finally, tie interpretation to a priori mechanistic predictions—for example (i) higher frontal θ burst rate and longer burst duration, with stronger frontal → parietal θ-PSI, predict better inhibition and set-shifting; (ii) faster IAF and higher α burst rate with lower α duty cycle over occipito-parietal cortex predict faster processing speed; (iii) steeper aperiodic exponent predicts better executive composite but explains unique variance distinct from oscillatory bursts; (iv) lower θ/δ DFA (weaker long-range temporal correlations) over parietal cortex predicts better updating/working memory—then register confirmatory tests in the replication cohort. By operationalizing oscillations as transient, networked events embedded in an aperiodic backdrop, and by quantifying temporal structure, directionality, and cross-frequency control signals with reliability and causal plausibility in mind, the field can replace ambiguous “more/less power” narratives with falsifiable mechanisms that map onto executive operations.

#### 7.8.3. Stronger Construct Modeling of EF

EF must be modeled as latent constructs rather than single-task scores, with preregistered measurement models and psychometrics strong enough to survive multiple testing and longitudinal analysis. Concretely, we recommend a unity–diversity framework in which a higher-order Common EF factor coexists with domain factors for Inhibition, Updating, and Shifting, plus auxiliary factors for Processing Speed and Reasoning to absorb variance not specific to EF. Each domain should be indicated by at least two independently implemented tasks to reduce method variance: for Inhibition, a color–word Stroop (200–300 trials; 50% congruent; color patches matched in luminance; 144 Hz display) and a stop-signal task (4 × 128 trials; 25% stop; one-up/one-down staircase to ~50% inhibition; SSRT computed by the integration method with go-omissions removed and go-RT slowing monitored by speed-emphasis probes every ~40 trials); optionally add an antisaccade block (≥240 trials; 70/30 anti/pro; eye-tracker validated saccade latency and error). For updating, include 2-back and 3-back (3 × 120 trials each; 30% targets; lure controls; stimulus duration 500 ms; ISI 2000 ms) and a complex span (operation/span with verified scoring rules for absolute and partial credit; list lengths 3–7; at least three sets per length). For Shifting, implement number–letter and color–shape tasks with single-task (A-only, B-only) and mixed blocks (ABAB…), switch probability 50%, cue–target SOA 150–300 ms jittered; compute switch costs (switch–repeat within mixed blocks) and mixing costs (mixed-repeat–single-task). For Processing Speed, include Digit–Symbol substitution (computerized; alternate symbol sets across waves) and TMT-A; for Reasoning, a 20–30 item matrices test with IRT-calibrated forms to enable linking. All tasks must be time-stamped with millisecond accuracy (≥1000 Hz USB polling; 144 Hz monitor), run in identical engines across sites, and counterbalanced to control order and fatigue; stimulus colors and luminance are spectrally calibrated; responses < 200 ms or >2.5 SD within condition are flagged, with winsorization only if preregistered. To address task impurity and speed–accuracy tradeoffs, primary outcomes are computational parameters derived from hierarchical Bayesian diffusion models (HDDM) or equivalent: drift rate *v* (quality of evidence), boundary *a* (caution), and non-decision time *t* (encoding/motor), estimated separately for key conditions (e.g., congruent/incongruent; go/stop context) with weakly informative priors, posterior predictive checks, and convergence criteria (R^^^ ≤ 1.05; effective sample size ≥ 1000); ex-Gaussian RT parameters and lapse rates are reported as sensitivity analyses. For the stop-signal task, we compute SSRT by the integration method (go-RT CDF quantile at *p* (respond|stop) with replacement of omissions by maximum RT), verify flat trigger failure rates, and include an adaptive “no-stop” speed block to detect strategic slowing; antisaccade is scored with both error rate and latency–variance decomposition. All tasks have alternate forms across waves (counterbalanced) and are IRT-linked so that longitudinal scale drift is minimized; practice effects are modeled explicitly using either a dual-baseline (T1/T0) design or retest-control subsamples, and a practice effect covariate is included in all growth models. The psychometric plan is preregistered: we fit confirmatory factor models (CFA) comparing (i) single common EF, (ii) three-factor EF, and (iii) bifactor (Common EF + orthogonal domain specifics), selecting by fit indices (CFI/TLI ≥ 0.95, RMSEA ≤ 0.06, SRMR ≤ 0.08) and parsimony; composite reliability (ω_total and ω_hierarchical) and H-coefficients are reported for each latent. We require measurement invariance both cross-sectionally (age, sex, education/SES strata) and longitudinally (configural → metric → scalar; partial invariance permitted with clearly documented freed loadings/intercepts); when full scalar invariance fails, we adopt alignment optimization and anchor items chosen a priori. Missing data are handled with FIML under MAR, with sensitivity multiple imputation if >10% missing in any indicator; outliers are addressed by robust estimation (MLR) and multiverse checks (e.g., trimming thresholds, accuracy floors). To integrate EEG and cognition without re-introducing task impurity, we treat EEG summaries as latent neural factors (e.g., an IAF factor from multiple parieto-occipital sites; an aperiodic-exponent factor from frontal/central clusters; a frontal-theta burst factor from multiple channels), each with reliability (test–retest ICC) ≥ 0.70; structural models then link neural latents to EF latents, not to single indicators, and report standardized paths, ΔR^2^ over demographics/education/baseline cognition, and calibration error. EF change is modeled with latent growth curves (intercept/slope; random effects) and RI-CLPM to partition between-person from within-person coupling between EEG and EF over time; slopes are expressed in IRT logits or latent-SD units per year, and we test age × EEG interactions on slopes. Differential item functioning (DIF) by SES/education and language background is screened with MIMIC models; any flagged items are downweighted or excluded in the IRT bank used for linking. Finally, to ensure transportability, we publish complete task scripts, IRT calibrations, parameter priors, and scoring code; we also report attenuation-corrected correlations (ρ = r/√(rel_x·rel_y)) alongside raw effects so readers can see the impact of reliability on EEG–EF associations. We make explicit claims that survive after accounting for reliability, invariance, and practice effects.

#### 7.8.4. Predictive Modeling with Real-World Endpoints

Build models that forecast outcomes people and clinicians care about, not just laboratory scores, using strictly leakage-free pipelines and externally validated performance. Define a priori three endpoint classes with clear ascertainment windows and censoring rules: (i) near-term functional efficiency (12–24 months) such as changes in processing-speed composites, complex IADL performance (timed bill-pay, medication box management), driving-simulator safety indices, digital behavior markers (smartphone typing latency variability, adherence to app-prompted routines), mobility (gait speed/dual-task cost), and work/academic productivity metrics; (ii) clinical transitions and events such as incident MCI or executive dysfunction diagnosis, progression on CDR-SB/TMT-B norms, falls requiring medical care, loss of driving privileges, hospital readmission within 30/90 days; (iii) time-to-decline trajectories in latent EF factors (inhibition, updating, shifting) and processing speed. For binary endpoints, specify the decision threshold tied to action (e.g., referral for neuropsychological evaluation, driving reassessment) before modeling; for continuous change, predefine minimal clinically important differences (MCID) in latent-SD units (e.g., 0.3 SD worsening/year); for survival endpoints, register index date (baseline EEG) and competing risks (e.g., death, relocation), with event-free censoring and adjudication procedures. Construct a baseline model with age, sex, education, SES, sleep and medication covariates, vascular risk, and cognitive-reserve proxies (vocabulary, occupational complexity), optionally adding brief cognitive screens; then add EEG blocks (IAF, aperiodic exponent/offset, band-limited burst metrics, directed θ/α connectivity, network efficiency, DFA/LRTC indices, microstate dynamics) built only from training folds to avoid information leakage. Standardize features within age-by-site strata using parameters learned in training folds; reduce dimensionality with within-fold PCA/PLS on feature families or use penalized models (elastic-net with α ∈ [0.1, 0.9]) and group lasso to respect spatial/feature families; control site effects with ComBat after QC (fit on training, apply to test) and include site as a random effect where mixed models are used. Use nested validation: inner 5–10-fold CV for hyperparameters; outer leave-one-site-out or blocked time-split (T0 train → T1/T2 test) to assess transport and temporal generalization; reserve a fully external cohort for final confirmation. Prefer transparent baselines (logistic/linear/multilevel, Cox/Fine–Gray for events) and report them alongside any nonlinear models (gradient boosting, random forest, calibrated SVM); apply probability calibration (isotonic or Platt) learned in the inner loop; quantify uncertainty with 1000× bootstrap on the outer estimates. Report, for binary endpoints, AUROC, AUPRC (when prevalence < 20%), Brier score, calibration intercept/slope, calibration curves, and expected calibration error; for continuous change, MAE/RMSE, R^2^, prediction interval coverage; for time-to-event, Harrell’s c-index, time-dependent ROC/AUPRC at 1/3/5 years, integrated Brier score, calibration-in-the-large, and Greenwood–Nam–D’Agostino tests. Always quantify incremental utility of EEG over the baseline: ΔAUROC/ΔR^2^/Δc-index with 95% CIs, IDI, and (category-free) NRI, and decision-curve analysis across clinically plausible thresholds to show net benefit; provide risk reclassification tables and number needed to evaluate to ground utility. Predefine events-per-parameter targets (EPP ≥ 50 for stable penalized regression; ≥200 for flexible learners), cap model complexity accordingly, and publish learning curves to demonstrate the data regime required for deployment. Guard against leakage by ensuring that any transformation (harmonization, PCA, feature selection, discretization, imputation) is fit only on training data; forbid mixing post-baseline information (e.g., T1 cognition) into features when predicting T1 outcomes; when using digital endpoints collected continuously, aggregate them in windows that end before the outcome window starts. For interpretability, favor sparse coefficients and standardized effect sizes; when using complex learners, apply permutation importance and SHAP computed in test sets only, aggregate at the feature-family level (e.g., “frontal θ burst rate”), and verify stability via bootstrap—avoid causal claims from local attributions. To support deployment, derive a parsimonious risk score (≤5–10 predictors) from the penalized model via coefficient shrinkage or step-down optimization that preserves ≥95% of full-model utility; present nomograms and a simple web/API calculator with confidence/credibility intervals and action thresholds mapped to interventions (e.g., additional testing vs. lifestyle program vs. clinical referral). Enforce fairness and transportability checks: stratify performance by age band, sex, education, SES, language background, and site; report subgroup AUROC/c-index, calibration slope, and equalized odds gaps with CIs; if drift is detected (population stability index PSI > 0.2 on key EEG latents or calibration slope < 0.8), trigger intercept-only or slope-and-intercept recalibration and schedule model revision; maintain an MLOps log of data shifts, calibration metrics, and intervention uptake. Use temporal validation (train T0 → predict T1; retrain T0–T1 → predict T2) to show stability across years; for survival models, evaluate dynamic prediction by landmarking at 12 and 24 months with updated EEG (if collected) to assess whether a change in EEG features improves discrimination (Δc-index) and reclassification. Handle missing predictors via within-fold multiple imputation (m = 20, predictive mean matching) with missingness indicators; predefine robust defaults when building risk scores (e.g., median-by-age-band imputation) and show sensitivity of decisions to imputation. Ensure privacy/feasibility with federated learning across sites (parameter averaging or FedProx) so models train without moving raw EEG; compare federated vs. pooled performance and release federated reference weights. Finally, anchor model claims to clinical utility: specify the workflow (who is screened, when, with what burden), estimate throughput and cost, and compute net benefit and cost per appropriate referral under realistic prevalence; preregister endpoints, metrics, thresholds, and analysis plans; publish code, trained weights, calibration plots, and full error analyses so that independent teams can reproduce performance and judge whether adding resting-state EEG meaningfully improves decisions beyond demographics, cognitive reserve, and brief cognitive testing.

Because predictive rsEEG models could be misused if interpreted as deterministic “risk labels,” we propose guardrails that treat EEG-derived risk scores as decision-support for clinical monitoring and resource allocation—not as a basis for exclusionary decisions. First, model outputs should be communicated as probabilistic, uncertainty-bounded estimates (e.g., calibrated risk with confidence/credibility intervals) and tied only to beneficial, low-harm actions (e.g., offering a fuller assessment, sleep optimization, cognitive health programs), rather than punitive thresholds. Second, deployment requires explicit use restrictions and governance (consent language that prohibits non-medical uses such as employment/insurance screening, human-in-the-loop review, and an appeal pathway for patients). Third, to reduce stigmatization and prevent reinforcement of social inequality, fairness auditing must go beyond reporting covariate adjustment: we recommend stratified calibration and error analyses by education/SES/language background and transparent reporting of subgroup false-positive/false-negative burdens; if disparities emerge, models should be recalibrated or redesigned (e.g., group-aware recalibration, revised features) before clinical use. Fourth, because education/SES may moderate EEG–EF links and act as proxies for structural disadvantage, these variables should be handled as context for interpretation (and for equitable calibration), not as justifications for diminished care; the goal is to avoid “risk phenotype” labeling that shifts responsibility onto individuals rather than directing support where reserve or opportunity has been constrained. Finally, privacy and regulatory compliance must be treated as first-order design constraints (data minimization, auditable versioning, and secure/federated learning where feasible), with prospective monitoring for dataset shift and unintended downstream harms during real-world use.

#### 7.8.5. Intervention and Translational Pathways

Translate resting-EEG findings into changeable targets by defining—upfront—(i) a biomarker phenotype to stratify participants, (ii) a target-engagement metric that must move in the expected direction during/after the intervention, and (iii) a behavioral endpoint with clinical relevance. Phenotyping uses the harmonized features above: individual alpha frequency (IAF), aperiodic exponent/offset, frontal-midline θ burst rate/duration, θ/β ratio at Fz, posterior α burst duty cycle, directed θ connectivity (frontal → parietal PSI), motor-network β power/coherence, and DFA/LRTC indices in δ–θ. Map phenotypes to actionable hypotheses: slow IAF and flattened aperiodic slope suggest reduced temporal sampling and lower E/I precision (prioritize α-centric engagement); high θ/β or low baseline θ with conflict-sensitive inhibition costs suggests deficient control signaling (θ-centric engagement); elevated resting β power/coherence over motor and inferior frontal systems with prolonged SSRT indicates over-stabilized motor networks (β-downregulation); high global synchrony and long path length at rest with slower reaction times flags inefficient integration (network-efficiency training). Target-engagement thresholds are specified a priori and verified in-session: for IAF, +0.2–0.3 Hz acute shift with stabilization across sessions; for exponent, +0.05–0.10; for θ, +15–30% burst-rate or +0.5–1 cycle burst-duration increase at Fz/FCz; for β, −10–20% power and −0.1–0.2 reduction in inter-MC/IFC coherence; for directed θ PSI, +0.05–0.10 frontal → parietal; for DFA in δ–θ, −0.05–0.10 toward less temporal stickiness. Behavioral co-primaries are diffusion-model drift-rate gains on inhibition/shifting tasks, SSRT shortening ≥ 20 ms, and latent EF slope improvements ≥ 0.3 SD over 3–6 months.

Interventions are deployed in mechanism-anchored modules with explicit dosing and montages. (1) Frequency-tuned non-invasive stimulation: Individualized α-tACS (IAF ± 0.1 Hz) over parieto-occipital cortex (POz/O1–O2 return), 1–2 mA peak-to-peak, 20–30 min, 3×/week for 4–6 weeks, with eyes-open fixation to encourage α event formation; expected engagement is faster IAF and reduced α duty cycle with improved processing speed and interference resolution. θ-tACS at 6 Hz (or θcenter from individualized band) with Fz–Pz or F3/F4–Pz montages for 20–30 min targets control signaling; requires in-session increases in θ burst rate/duration and frontal → parietal PSI; pair with brief conflict tasks (flanker/stop) during stimulation to maximize state-dependence. β-suppression neuromodulation uses amplitude-modulated tACS centered at 20 Hz over bilateral M1/I, FC, or inhibitory rTMS over the pre-SMA/IFG (if available in the trial), with target engagement defined as acute βe-β power/coherence reduction and SSRT gains. (2) Closed-loop neurofeedback: real-time displays of θ/β at Fz, α burst duty cycle at POz, or motor-β amplitude at C3/C4 using 32–64-channel systems; 20 min blocks × 2 per session, 12–15 sessions over 6–8 weeks. Feedback algorithms enforce event-based reinforcement (reward only when burst criteria are met) and adapt thresholds within a session to maintain ~60–70% success; require session-wise learning slopes and between-session retention as success criteria. (3) Task-anchored cognitive training: Inhibition/shifting suites (Stroop/stop/antisaccade, number–letter) with adaptive difficulty titrated to maintain ~80% accuracy, 30–45 min, 3–4×/week for 6 weeks; hypothesized transfer is via increased frontal θ bursting and strengthened directed θ connectivity; verify by pre/post EEG with burst and PSI metrics. Updating/WM training (2-/3-back + complex span) is reserved for profiles with low parietal DFA and weak posterior α-burst control; it requires increases in posterior α-burst rate and reductions in DFA exponents. (4) State modulators that shift the exact EEG dimensions: Sleep optimization (CBT-I components, 6 sessions) and physical-activity prescriptions emphasizing incidental activity (step goals, stair prompts) and 2×/week strength/balance; success is movement of δ/θ down and α up in fronto-temporal leads with faster mean frequency, mirroring the “active” pattern in older adults. Cholinergic augmentation (trial context only, e.g., donepezil/rivastigmine micro-dosing) is considered for profiles with emergent δ increases and poorer memory/inhibition, with engagement defined as δ reduction and steepening of the exponent; pharmacologic use remains investigational here and is tied to safety monitoring.

Trial architecture follows a target-engagement → efficacy → optimization sequence. A two-arm Phase 0/1 (active vs. sham; N ≈ 60–80 per phenotype) establishes whether the EEG target moves as specified and whether the proximal behavioral mediator (drift rate or SSRT) changes; randomization is stratified by site/age/education; sham procedures mimic sensations; both EEG and mediator thresholds predefine success. Mechanistic Phase 2 uses a 2 × 2 factorial (e.g., θ-tACS × inhibition-training) to test additivity/synergy with diffusion-model parameters as mediators; sample N ≈ 160–240 total (powered for small interaction, f^2^ ≈ 0.02) with blinded assessors and pre-registered analyses. Adaptive deployment uses SMART: start with the modality matched to phenotype; non-responders (no EEG engagement by session 4, or <10 ms SSRT gain) are re-randomized to augment (add training) or switch (e.g., θ-NF ↔ θ-tACS), yielding decision rules for stepped care. In parallel, N-of-1 multiple-crossover micro-randomized trials (ABAB… with 2–3-day washouts) tune stimulation frequency (±0.5 Hz around IAF/θcenter), intensity (1.0–2.0 mA), and montage per person, with drift rate as the daily proximal outcome; individual posterior estimates feed the group policy. Across all designs, analyses are mediation-first: EEG engagement → task-level mediator → EF endpoint, with sensitivity to unmeasured confounding; claims of efficacy require both the path a (EEG change) and b (mediator → behavior) to be non-zero.

Implementation emphasizes clinically plausible workflows. Screening uses a 20 min resting EEG and a 30 min EF battery to assign phenotype and baseline risk; interventions are delivered in 6–8 weeks, 2–4 sessions/week, with remote-capable options (validated 16- to 32-channel dry/wet systems) for neurofeedback and training; each session logs device QC (impedances, bad-channel count, retained minutes) and real-time engagement metrics. Safety is continuously monitored: stimulation adverse events (skin irritation, headache, phosphenes), blinding integrity, and cognitive/affect checklists; stopping rules include sustained sleep disruption, mood deterioration, or paradoxical EEG changes (e.g., β rebound > 20% across two consecutive sessions). Equity is built in by subsidizing equipment and travel, offering tele-delivery, pacing sessions for older adults, and verifying that calibration and performance are stable across SES/education/language strata; if subgroup calibration drifts (e.g., different baseline exponent distributions), parameter targets are age/education adjusted. Translation to practice requires parsimonious decision aids derived from the predictive model based on given age/education and baseline EEG latents; the tool recommends the first-line module, dosing (sessions/week, minutes), and a week-4 decision point; it outputs expected gain bands and confidence intervals, and triggers referral if early engagement fails. Regulatory and data governance are addressed by treating algorithms as SaMD with versioning, pre-specification of updates, immutable audit logs, and consented data sharing; deployment uses federated learning so clinics can improve policies without exporting raw EEG. Finally, success is defined not only by statistically reliable EF improvements but by net clinical benefit: improved everyday functioning (IADLs, mobility dual-task cost), fewer safety-critical errors (driving, falls), and durable EEG normalization (IAF speed, steeper exponent, healthier θ/β balance) at 3–12 months, with cost-per-appropriate-referral and number-needed-to-treat reported to justify uptake.

#### 7.8.6. Multimodal Integration (EEG + fMRI/MRI/PET/fNIRS)

A notable gap in the current rsEEG literature is the limited integration of electrophysiological markers with complementary neuroimaging measures that index the same distributed networks supporting executive readiness. Mechanistic synthesis would benefit from multimodal designs that explicitly map rsEEG “tempo” (individual alpha peak/peak frequency), “noise” (aperiodic 1/f parameters), and connectivity features onto canonical large-scale systems such as the salience network and central executive network, which interact with the default-mode system during cognitive control and network switching [239,240,241].

Convergent evidence already indicates that spontaneous EEG fluctuations relate to fMRI resting-state networks: simultaneous EEG–fMRI work has linked ongoing spectral power dynamics (especially in the alpha range) to resting-state connectivity and network activity, supporting the idea that rsEEG indices can serve as temporally resolved readouts of large-scale network states [242,243,244]. Importantly, emerging studies also show that aperiodic EEG components are not merely nuisance variance: in simultaneous EEG–fMRI, aperiodic measures exhibit associations with distributed hemodynamic patterns (including salience/prefrontal-linked activity), suggesting a bridge between putative E/I–E/I-arousal-related spectral parameters and network-level fMRI physiology [245,246].

Beyond fMRI, multimodal extensions can strengthen biological interpretation. Structural MRI and diffusion imaging can anchor EEG features to individual differences in network anatomy and structural connectivity (supporting interpretation of EEG connectivity markers), while FDG-PET can link electrophysiological phenotypes to regional and network-level metabolic demand; prior EEG–FDG-PET work has reported relationships between alpha-band metrics and cortical glucose metabolism in clinical risk contexts [247,248]. Finally, concurrent EEG–fNIRS provides a pragmatic pathway for scalable and portable multimodal monitoring, with established methodological guidance for joint acquisition/analysis and growing evidence that the modalities capture complementary aspects of network function [249,250].

Practically, we suggest that future mechanistic studies prioritize (i) simultaneous EEG–fMRI (or EEG-fNIRS where MRI is not feasible) to relate EEG tempo/noise/connectivity to salience–executive network dynamics and (ii) multimodal fusion approaches (e.g., joint network models and cross-modal calibration) that test whether EEG-derived phenotypes track hemodynamic/metabolic network states and improve prediction/monitoring beyond any single modality [250,251].

## 8. Conclusions

Across 63 studies, resting-state EEG offers a low-cost, millisecond-resolved window onto trait-like “executive readiness.” The most informative picture is not any single band but a composite profile capturing (i) tempo (individual/peak alpha frequency (IAF/PAF), (ii) neural “noise” (the aperiodic 1/f slope and offset), and (iii) wiring (frequency-specific coupling and network topology). Faster alpha pace, steeper aperiodic slopes, and globally efficient—but not over-synchronized—networks consistently align with better executive speed, interference control, and consistency, whereas misplaced or excessive coupling (e.g., rigid fronto-posterior theta; globally heightened alpha/gamma synchrony) predicts slower or less adaptable performance.

Links are domain-specific. For working memory, IAF—not alpha power—is the clearest positive marker, primarily for manipulation-heavy spans; supportive within-frontal beta/gamma coherence helps, while excess fronto-posterior theta selectively undermines sequencing/updating. Decision speed and consistency are better indexed by topology (shorter paths, higher efficiency) than raw power. In inhibition, motor-network beta at rest (power/coherence/global “efficiency”) forecasts worse stopping, whereas left-lateralized prefrontal β/α and faster IAF favor interference resistance and vigilance sensitivity.

Moderation by age, development, and reserve is the rule. Frontal theta can support children’s working memory, yet becomes a liability in healthy aging; slowing alpha pace in later life strengthens the link between resting markers and speeded control. Cognitive reserve (education/experience) shifts set-points toward faster temporal regimes and stronger eyes-open ↔ eyes-closed gating in alpha/theta connectivity, sometimes inverting 1/f–behavior relations despite similar test scores. These patterns argue for modeling age and reserve as interacting moderators rather than nuisance covariates.

Equally important is what rest cannot tell us. Resting EEG does not independently diagnose executive impairment; simple ratios (e.g., theta/beta) are context-sensitive and show age-dependent reversals. It indexes baseline readiness rather than trial-by-trial tactics, and scalp power alone cannot localize sources or disentangle oscillations from background slope without explicit periodic/aperiodic decomposition. Findings also depend on EF task demands (maintenance vs. manipulation, stopping vs. interference) and outcome framing (speed vs. accuracy); weak behavioral psychometrics cap brain–behavior correlations.

Methodological implications follow. Biomarker-quality recordings should include ≥5–6 min eyes-closed (plus eyes-open), ≥32 channels, vigilant artifact/drowsiness control, and mandatory periodic–aperiodic separation. Connectivity should rely on lag-insensitive metrics and be summarized by graph efficiency/path length and alpha-network temporal variability; analyses must separate speed from accuracy and distinguish WM maintenance, manipulation, and spatial updating.

The literature’s main limitations—small, cross-sectional, and often homogeneous samples; heterogeneous pipelines; oscillatory/aperiodic confounds; and inconsistent directionality—are solvable. Harmonized, adequately powered, and longitudinal designs that pre-register periodic/aperiodic models, stratify by age and reserve, and prioritize robust EF batteries will enable precise, generalizable estimates (|r| ≈ 0.10–0.20) and credible change–change inferences.

Taken together, the pragmatic takeaway is a multi-feature signature: faster alpha pace; steeper 1/f slopes with appropriate offsets; efficient, flexibly reconfigurable alpha-band networks with modest global synchronization; supportive within-frontal beta/gamma coherence (with fronto-posterior theta treated as a risk factor for manipulation); and prefrontal β/α asymmetry indexing a person’s default balance between phasic switching and sustained maintenance. Interpreted through the lenses of age and reserve, this composite best captures who learns faster, reconfigures sets more readily, sustains attention more stably, and resists interference, i.e., the traits that underwrite everyday executive success.

Finally, clinical translation should proceed cautiously but optimistically: use the composite as a stratification and monitoring tool—not a diagnosis—to guide and evaluate interventions (training, lifestyle, neuromodulation). Success means convergent improvement in EF alongside movement toward the target resting profile (IAF ↑, 1/f steepening, healthier slow/fast balance, more efficient/flexible networks) with equity-minded deployment and rigorous validation against meaningful real-world outcomes.

## Figures and Tables

**Table 1:** Studies included in the review.

Main Associations	Cognitive/EF Measures	EEG Metric (s)	Resting EEG Protocol	Population/Notes	Sample (n, Age, Sex)	Study
Lower δ/θ ↔ higher WAIS subtests (esp VC and WM). Excess frontal/posterior δ ↔ poorer VC; higher frontal θ ↔ poorer WM. Higher α ↔ better Processing Speed and Perceptual Organization. β few/inconsistent.	WAIS-III: Verbal Comprehension, Working Memory, Perceptual Organization, Processing Speed; FSIQ	Absolute and relative band power (age Z)	Eyes closed; 19 electrodes; bands: δ 1.5–3.5, θ 4–7.5, α 8–12.5, β 13–19; AP and RP	Community-dwelling; age-standardized EEG Z-scores	27 healthy older adults; mean 67.2 y; 12 M/15 F	[74]
Lower drift rate (worse recognition) ↔ higher occipital δ and lower parietal θ. No relationship with One Back WM. Age ↑ associated with ↑ δ/θ; age not tied to memory scores.	CogState: One Card Learning (recognition memory; drift rate), One Back (working memory)	Band power by region (occipital/parietal emphasis)	Eyes closed; bands: δ 2–4, θ 4–8, α 8–12, β 12–30	Subjective cognitive decline; no dementia	44 healthy older adults with SCD; mean ~74 y; 7 M/37 F; MMSE ≥ 25	[75]
Higher frontal/parietal θ ↔ better verbal recall, reasoning (SPM), category fluency, and more stable SART performance. No δ/α relationships; β showed limited negative correlations with reasoning (SPM) and WM span at Pz.	RAVLT; RBMT; Raven’s SPM; Category Fluency; Digit Span; SART; MMSE; NART	Relative band power by site	Eyes closed 4 min; F/C/P sites; bands: δ 1–3.5, θ 4–6.5, α 7.5–12.5, β 13–30; relative power	Extensive neuropsychological battery	73 healthy adults; 56–70 y (mean ~61); mixed sex	[76]
In women, lower resting θ complexity ↔ better WM (r = −0.67). Women also showed stronger α1 desynchronization and higher complexity; α2 no effects.	Picture WM (12 items; recall)	Band-specific complexity (Dcg); desynchronization	Eyes closed; bands: θ 4–7, α1 8–10, α2 10–13; linear and nonlinear (Dcg complexity)	Within-subject rest vs. WM recall	21 healthy adults; mean 62.5 y; 9 M/12 F	[77]
Frontal α peak frequency predicts reverse digit span beyond age/sex/education (~+0.21 digits per +1 Hz). APF slows with age; reverse span declines most with age.	Computerized Digit Span (forward and reverse)	Frontal and posterior alpha peak frequency	Eyes closed 2 min; anterior/posterior electrodes; α peak frequency (APF)	Lifespan sample	550 healthy participants; 11–70 y (mean 33); mixed sex	[78]
Older adults: worse spatial WM; lower GE-variance; lower occurrence of C, C′, D; reduced transitions into C/C′/D—suggesting reduced engagement of WM/attention-related networks.	Real-world allocentric spatial WM task (arena)	Microstate occurrence, transitions, global explained variance	Eyes open and closed; microstates A, B, C, C′, D; clustering	High-density EEG microstates; allocentric spatial WM	Young: n = 20 (25–30 y); Older: n = 25 (64–75 y)	[79]
Eyes-open aperiodic slope ↔ higher g. Steeper slopes ↔ faster RTs (eyes-open and closed). No strong links to single WAIS domains.	WAIS-IV domains; general g; Hick RT subset (n = 110)	Aperiodic slope (global component ~60% variance)	Eyes open and closed; aperiodic slope (1/f) across scalp	Dense 64-ch EEG; eyes open/closed	166 undergraduates; 18–52 y (mean ~23); ~60% women	[80]
Higher β power (MC/SC/IFC), higher β coherence, and higher β global efficiency ↔ longer SSRT (poorer inhibition).	Stop-Signal Task (SSRT)	β power; β coherence; β network global efficiency	13 min (12 min analyzed) eyes closed; β 15–30 Hz; regional and network measures	Motor inhibition (SST) focus	30 healthy young adults; mean 21.6 y; 8 M	[81]
Low baseline θ → more false alarms under conflict; high baseline θ protective. Larger conflict-related increases in total θ for low-baseline group localized to superior parietal/angular gyrus. Effects specific to total θ (not evoked/PLF).	Go/No-Go with auditory conflict (visual ‘DRÜCK’/’STOPP’)	Baseline total θ; evoked θ; PLF; source (parietal BA7/BA40)	Baseline θ power; ability to upregulate θ vs. baseline	Conflict-modulated Go/No-Go; theta upregulation challenge	66 healthy adults; ~25.7 y; 33 M/33 F	[82]
Rest θ highest in children, decreases with age. No-Go > Go θ from SMA/SFG. After ~10.7 y: higher rest θ ↔ stronger task θ; after ~19.5 y: higher rest θ ↔ fewer false alarms.	Go/No-Go (70% Go; 30% No-Go)	Rest θ; task-related θ sources (SMA/SFG, BA6)	Eyes open 2 min; θ 4–7 Hz	Developmental modulation of rest–task θ link	166 participants; 8–30 y; 95 F	[83]
Upper-β and γ (left central/temporal) → slower RT and more variable vigilance. Parietal α → slower but steadier performance. Mid/upper-β in some regions → better error inhibition and stability. Temporal γ → lapses and variability.	SART: commission/omission; RT; vigilance (TVS/CVS)	α, β (low/mid/upper), γ power ratios by region	2.5 min eyes open + 2.5 min eyes closed; 64-ch; band-power ratios across 14 ROIs	105 min SART vigilance	10 healthy adults; 22–45.5 y (mean ~30); 6 F/4 M	[84]
Higher resting β-2 → longer RTs and lower accuracy (both sessions). Higher β-2 ↔ stronger resting connectivity but less task-reconfiguration (rigid networks). Lower β-2 ↔ better flexibility and performance gains.	Visual search (RT); shooting accuracy (pneumatic gun)	Global β-2 power; frontoparietal/frontooccipital connectivity and flexibility	Eyes open at rest (and during tasks); emphasis on β-2 (22–29 Hz)	Two sessions ~2 months apart; training between	36 healthy men; mean ~22 y	[85]
Higher posterior α → broader/global bias. Higher right parietal/occipital β → narrower/local bias. θ not predictive.	Navon letters (global vs. local)	Absolute band power (regional)	Rest α (8–12), β (13–30), θ (4–7); posterior/right-lateral focus	Alternating eyes open/closed blocks	48 right-handed undergraduates; mean ~19 y; majority female	[86]
Older adults: slower IAPF; lower exponent/offset. In older low-edu: higher exponent/offset → better attention and WM. In older high-edu: higher exponent → worse speed and WM. Frontal power in older high-edu → faster alertness RT.	Processing speed (alertness; TMT-B); Working memory (WM_TAP); CVLT (delayed)	Aperiodic slope (exponent) and offset; IAPF	Aperiodic exponent and offset; periodic IAPF; frontal exploratory analyses	Education as cognitive reserve moderator	179 total: Young high-edu n = 123 (20–35 y); Older high-edu n = 24; Older low-edu n = 32 (60–77 y)	[87]
Younger > older in resting θ at FCz/Cz/C4; overall accuracy high; no significant correlations between resting θ and recognition performance.	Recognition (old/new) after 4- vs. 8-word lists	Rest θ power across sites (FCz, Cz, C4 emphasis)	θ sub-band 4.88–6.84 Hz; 32-ch; ROI FCz	Modified Sternberg WM; resting baseline eyes open	28 total: 14 young (mean 21.9 y), 14 older (mean 68.4 y); ~equal sex	[88]
Lower θ/β ratio (i.e., relatively more β) → better orienting. Stronger parietal δ–β coupling → higher self-reported attentional control. No link to executive (conflict) score.	ANT-I (executive, orienting, alerting); Attentional Control Scale	Theta/beta ratio (frontal/parietal); delta–beta coupling (frontal/parietal)	8 min baseline alternating eyes open/closed	ANT-I plus self-report; trait anxiety measured	110 healthy adults; 18–55 y (mean ~29); 83 F/27 M	[89]
Higher cognitive CR → greater long-range low-α LLC (occipital–cortex) and better sustained attention and SWM. Higher social CR → greater local θ/low-α LLC (EO) and better SWM strategies. Physical CR showed weak/atypical patterns.	CANTAB: RVP (sustained attention), PAL (episodic), SWM (spatial WM)	Local and long-range LLC in δ, θ, low/high α, low/high β, γ	Eyes closed; high-density; lagged linear connectivity (LLC) across bands	Cognitive/social/physical lifestyle factors as CR proxies	104 adults; 35–75 y (mean ~57); ~75% women	[90]
With age: reductions across bands, esp δ/θ amplitude and δ power. In oldest, δ ↑ ↔ more memory errors; δ power negatively correlated with CSF AChE (↓ cholinergic function). Longitudinal: subset with learning decline showed δ increases.	Digit Symbol; Block Design; TMT A/B; word list learning/recognition	Amplitude and power (absolute/relative) by band	Eyes closed; bands: δ 1.5–3.9, θ 4.1–7.3, α 7.6–13.9, β 14.2–20	CSF AChE in subset; temporo-occipital derivations	52 healthy; 20–91y; subset (n = 15, ≥50 y) 2-year follow-up	[91]
Active group → higher Processing Speed and Performance IQ; EEG profile: less δ/θ, more α (frontotemporal), faster mean δ frequency. Passive group: higher δ RP (F7, T3), higher θ AP/RP (C4, F4, T3, Fz), lower α AP/RP (F3, F7, T3).	WAIS-III-R indices; subtests (Matrix Reasoning, Digit–Symbol Coding, Picture Arrangement)	Absolute and relative power; mean frequency by band	Eyes closed; 19 electrodes; FFT power in δ/θ/α/β	IPA via Yale Physical Activity Survey; similar education/CR/SES	97 older adults ≥ 60 y (mean ~67); 64 F/33 M; Active (n = 48) vs. Passive (n = 49) by incidental physical activity	[92]
No drift toward drowsiness across 6 min. MCI > HC in posterior θ. In MCI, higher α/β ↔ poorer CVLT memory; in HC, higher θ ↔ lower MoCA. δ trend higher in MCI (ns).	MoCA (global); CVLT-II (memory)	Band power by region; posterior emphasis for θ	Eyes closed 7 min (6 min analyzed); 31 electrodes; δ 0.1–3, θ 4–8, α 8–12, β 12–28	Excludes 4 for noise; segment-length comparison (2 s vs. 8 s)	40 older adults: 20 HC (~72 y), 20 MCI (~76 y)	[93]
Age: younger L > R coherence; older R > L. Eyes-closed > open coherence stronger in high-CR. Younger: low-CR > high-CR coherence; older: high-CR > low-CR. High-CR better on memory, fluency, WM.	CVLT (memory), Digit Span (WM), Verbal Fluency	Intra-/interhemispheric coherence across bands	Eyes open and eyes closed; coherence via NeuroGuide across delta (1–4), theta (4–8), low α (8–10), high α (10–12), β (12.5–25), γ (30–50) Hz	Cognitively normal adults; grouped by cognitive reserve (education, NART-R verbal IQ)	n = 90; 45–64 y (M ≈ 58.5); 58 women	[94]
Higher frontal β → better Digit Span Fwd/Seq; frontal γ → better Digit Span Fwd; frontal δ → fewer CVLT recognition errors and faster TMT-B. Posterior δ → fewer CVLT errors; posterior γ ↘ with Digit Span Seq. Fronto-posterior (esp θ) ↑ → worse WM (Digit Span Seq).	WAIS Digit Span (Fwd, Seq), CVLT-II (learning/recognition), D-KEFS Verbal Fluency, TMT-A/B	Regional and long-range coherence by band	Eyes closed; coherence within frontal, posterior, and fronto-posterior; delta (1–4), theta (4–8), α (8–12), β (12–25), γ (30–50) Hz	Healthy older adults; coherence vs. cognition	n = 66; 50–88 y (M ≈ 67); ~64% female	[95]
Higher WM → lower DFA exponents in θ and δ (strongest posterior); weaker effect in α; none in β/γ; effects independent of power and not specific to FCz.	WAIS-IV Digit Span and Arithmetic (WM)	DFA scaling (Hurst) exponents per band; spatial modeling	Eyes closed; 64-ch; bands: δ (2–3.5), θ (4.5–6.5), α (8–13), β (18–26), γ (36–46) Hz	Healthy adults; working memory ability vs. long-range temporal correlations (LRTCs)	n = 54; 18–52 y (M ≈ 25); 32 women	[96]
Young: higher TAR → better STM (and trend for reasoning). Older: relation absent/reversed (higher TAR → poorer reasoning). TAR lowest in eyes-closed rest.	Word-list STM (4 trials), Raven’s Matrices (reasoning)	TAR = θ/α power; band powers across conditions	19 electrodes; eyes open and closed rest and during memory/reasoning tasks; θ (4–8), α (8–12)	Healthy young vs. older; examined theta–alpha power ratio (TAR)	n = 36; young 20–29 (n = 16, M ≈ 20.7), older 70–79 (n = 20, M ≈ 72.9)	[97]
Greater baseline EC-θ → worse future cognition (lower CAMCOG, slower TMT). Reduced α-reactivity → poorer future language and global cognition. EEG alone predicted ~43% variance in future cognition; cognitive tests ~92%; combined ~93%.	CAMCOG (global), WMS, Boston Naming, TMT; repeat at follow-up	Theta power; alpha reactivity indices	Eyes closed, eyes open, and memory activation task; focus on θ (4–8) power and α (8–13) reactivity (EO/EC and task)	Elderly AD, amnestic MCI, controls; longitudinal (~20 months)	Baseline: AD n = 14, MCI n = 20, Controls n = 24; 20-mo FU: 8/12/21; >60 y	[98]
Baseline: higher global coherence ↔ better cognition (esp memory; some attention/EF) beyond age. Slowing score ≈ ns. 5 y FU: neither global coherence nor slowing predicted cognition/decline (limited by attrition and n = 22).	ADAS-Cog; CERAD fluency and TMT-A/B; WAIS Digit Span and Digit–Symbol; MVGT recall; Everyday Cognition WM; TICS-M at FU	Global coherence; slowing ratio; localized band/region analyses	Resting awake; global coherence 1–30 Hz; slowing score = (δ + θ)/(α + β)	Older adults with memory complaints/at risk; longitudinal 5-year subset	n = 70; 60–88 y (M ≈ 7 2); FU n = 22 at 5 y	[99]
Age → slower IAF. Faster IAF → better SART discrimination (independent of age/sex/education); no effect for RVP. IAF mediated age → SART. Aperiodic/aIAP not linked to attention after correction.	SART (inhibitory control sensitivity), RVP (vigilance)	IAF; aIAP; aperiodic exponent and offset	Resting-state; metrics include IAF, aperiodic-adjusted α power (aIAP), aperiodic exponent and offset	Healthy older adults; sustained attention (SART, RVP)	n = 96; 50–84 y (M ≈ 65); majority women	[100]
Older: lower α power and reduced 1/f offset; θ no age diff. Higher α power → better proactive control; higher 1/f offset → modestly better proactive control. Higher θ power → larger congruency effect (worse reactive control).	Cued Flanker: Reactive Control (congruency), Proactive Control (conflict expectation)	Alpha power; 1/f offset; theta power	1 min eyes open + 1 min eyes closed; α (8–12), θ (4–8), 1/f activity	Healthy; proactive vs. reactive control in cued flanker	n = 39; younger 18–30 (n = 20, 14 F), older 65–80 (n = 19, 11 F)	[101]
No associations of IAF, α/θ power, or 1/f offset with interference effects in Stroop/Navon; Bayesian evidence favored null.	Stroop; Navon (local vs. global interference)	IAF; α power; θ power; 1/f offset	64-ch; ~2.5 min eyes open and eyes closed; extract IAF, α/θ power, 1/f offset	Test IAF/oscillatory and aperiodic markers vs. inhibitory control	n = 127; young adults (M ≈ 24); university sample	[102]
Older → flatter slopes (except occipital) and lower RBANS. Spectral slope mediated age effect on Coding (Processing Speed) only; no mediation for RBANS total, Attention domain overall, or Delayed Memory.	RBANS total + domains (Immediate Mem, Visuospatial, Language, Attention, Delayed Mem); focus on Coding Subtest (Processing Speed)	Aperiodic spectral slope (1/f)	Eyes closed; spectral slope from frontal/central/parietal/occipital sites	Healthy; spectral slope vs. RBANS domains	n = 44; young < 35 (n = 21, M ≈ 23), older > 59 (n = 23, M ≈ 71)	[103]
Age ↓ exponent and offset and ↓ cognition. Lower offset ↔ poorer Verbal Fluency (effect apparent from ~33 y; stronger with age). Higher exponent ↔ better Verbal Fluency and composite EF/WM/psychomotor speed, controlling for age.	CWIT (inhibition), WAIS Digit Span, RAVLT, TMT, Verbal Fluency	Aperiodic exponent (slope) and offset (broadband power)	4 min eyes closed; exponent and offset estimated; PCA/clustered scalp regions	Lifespan sample; aperiodic activity vs. cognition	n = 111; 17–71 y (M ≈ 37.5); 68 women	[104]
IAF correlated with g (r ≈ 0.40) across ages; no specific links to subdomains after accounting for g; effect size similar in young and older.	Berlin Intelligence Structure (Perceptual Speed, Memory, Reasoning) modeled under g via SEM	Individual alpha frequency (IAF)	Eyes open and closed; repeated ~6 months apart	Healthy; test IAF vs. general intelligence (g)	COGITO: total n = 287 (145 young 20–31; 142 older 65–81); EEG subset n = 85 (45/40), 2 timepoints	[105]
Older: slower α peak; ↑β power; reduced parietal α asymmetry. Young: higher resting β → slower prosaccade RT. Older: higher central δ → better TMT-B. Broad age-dependent EEG–behavior links.	Digit and spatial span (WM), TMT (switching/inhibition), word fluency, NART; eye tasks (pro/anti-saccade, Go/No-Go)	Alpha peak frequency; α/β/θ/δ power; parietal α asymmetry	Eyes open and closed; power in δ (1–4), θ (4–8), α (8–12), β (12–20); alpha peak frequency; hemispheric asymmetry	Healthy aging; EEG + oculomotor and cognitive tasks	Final n = 75; young 18–30 (n = 31, M ≈ 24), older 61–90 (n = 44, M ≈ 71)	[106]
Faster RTs ↔ ↑δ (and marginal ↑θ) at left parietal (P7). Higher α/β at right parietal (P8) and γ at right frontal (AF4) ↔ lower accuracy. ↑α coherence (R parietal–L frontal) ↔ slower RTs; ↑δ/θ fronto-temporal coherence ↔ better accuracy.	Accuracy and RTs on Bluegrass delayed match-to-sample	Band powers by site; coherence (inter-site) by band	60 s eyes open + 60 s eyes closed; bands δ (1–4), θ (4–8), α (8–13), β (13–28), γ (28–46); coherence between pairs	Healthy older; portable 14-ch Emotiv; Bluegrass delayed match-to-sample	n = 43; 60–91 y (M = 71.6); 20 men, 23 women	[107]
Interference resolution ↑ with higher IAF (R frontal, bilateral parietal/temporal, R cingulate). Faster Processing Speed ↔ lower R-frontal α power. No EEG links for episodic memory. Age−, education + (esp for interference resolution).	TMT, Stroop, WMS, Vocabulary → factors: Processing Speed, Episodic Memory, Interference Resolution	IAF; α power (regional esp R frontal); θ power; 1/f slope	Eyes closed 20 min; metrics include IAF, α/θ power, 1/f slope	Large healthy aging cohort; 20 min eyes-closed resting EEG; oscillatory vs. aperiodic separated	n = 1703; 60–80 y (M ≈ 70); 880 women	[108]
Higher vocabulary ↔ stronger alpha; lower alpha ↔ poorer vocabulary (pronounced in lower-SES). Higher frontal theta ↔ better working memory; lower SES tended to show reduced theta and weaker WM.	Vocabulary; Digit Span (working memory)	Alpha power; frontal theta power	Resting state (task-free; eyes not specified)	SES-diverse children (higher vs. lower SES)	N = 90, 45 8–15-year-olds from low-income homes and 45 age and sex matched children	
					from higher income homes	
With age: ↓theta, ↑alpha, faster alpha peak. EC: ↑theta/alpha; EO: ↑beta/gamma. Lower theta/beta ratio ↔ stronger EF (holds controlling for age and verbal ability).	Minnesota Executive Function Scale (MEFS)	Theta, alpha, beta, gamma; peak alpha frequency; theta/beta ratio	Resting EEG, eyes closed (EC) and eyes open (EO)	Typically developing; predominantly white, middle-class	n = 162; 3, 4, 5, and 9-year-olds; sex not stated	[110]
EO: Higher ATR and BTR → better inhibition; EO: Higher BTR → better planning (ATR ns after controls). EC: no relations. WM: no EEG predictors.	Inhibition (Receptive Attention); WM (1-back drift rate); Planning (Planned Codes); Verbal Naming Speed (Control)	Frontal alpha/theta ratio (ATR); beta/theta ratio (BTR)	32-channel EEG; EO and EC	Typically developing; right-handed; grades 2–3	n = 59; 7–9 yrs (M = 8.63, SD = 0.56); 24 girls/35 boys	[111]
Theta patterns specifically linked to income and to working memory ability; multivariate EEG–SES–cognition relationships detectable beyond univariate analyses.	PPVT (vocabulary); Reverse Digit Span (WM)	Theta (4–6 Hz), alpha, beta, low/high gamma	rsEEG, eyes closed; child-optimized preprocessing; ML (SVR/SVM)	School-aged; SES indexed by income and maternal education	Final n = 161 (of 195); 8–15 yrs (≈11), 90 female	[112]
No behavioral group differences. High CR showed lower theta (parietal EC; occipital EO; temporal both) and lower delta (temporal EO) vs. low CR; no alpha/beta differences.	Broad neuropsych battery (attention, WM, flexibility, visuospatial, fluency, memory)	Delta, theta, alpha1, alpha2, beta (power)	3 min EO + 3 min EC; regional analyses	Healthy older adults; CR by Cognitive Reserve Questionnaire (median split at 16)	n = 74; 55–74 yrs; High CR: 41 (21 M/20 F); Low CR: 33 (15 M/18 F)	[113]
Baseline PAF correlates with same-day Digit Span (not cross-day). Low baseline PAF ↑ after task (state “correction”). Both baseline PAF and WM higher on day 2 (practice effects).	WAIS-R Digit Span (WM)	Peak alpha frequency (PAF)	EC baseline pre-task and post-task (two days)	Two sessions; state-related readiness focus	n = 19; 19–23 yrs; college students	[114]
After correction, no robust WM associations with microstate metrics; age and gender far stronger predictors of microstate properties.	Working memory (2-back accuracy); mood, personality, attention covariates	Microstates A–E; GEV, mean duration, occurrence, transitions	16 min resting; alternating 1 min EC/EO; 62-channel	Healthy adults	n = 191; Young 20–35 (n = 128), Older 59–77 (n = 59); balanced gender	[115]
Greater left-lateralized β/α (higher left vs. right) → smaller Stroop effects (better interference resistance); localized to left prefrontal (pre-SMA, middle frontal, IFJ).	Verbal and Spatial Stroop (RT/errors; Stroop effect)	Beta/alpha (β/α) power ratio hemispheric asymmetry (Right–Left)	Resting EEG	Interference control focus	n = 56; healthy university students; sex not stated	[116]
No significant links between microstate C/D metrics and EF; small non-sig. trend (more D occurrence → lower EF). Demographics predicted EF (↑education better; ↑age worse).	9-task EF battery (inhibition, updating, shifting); composite and latent EF	Microstates A–D; duration, occurrence, coverage, GEV	Resting EO/EC; microstate analysis on EC	Healthy young adults	n = 140; 18–35 yrs (M ≈ 24.7); ≈16 yrs education	[117]
Microstate A duration ↑ → higher fluid intelligence (LPS-2); transitions D ↔ C ↑ → lower fluid intelligence. Microstate A duration/occurrence/coverage ↑ → higher verbal fluency (RWT). Microstate B occurrence and transitions E→B (+)/E → C (–) → higher WST. Regression and SEM confirmed predictiveness.	CVLT (memory), TMT (flexibility), WST (crystallized), LPS-2 (fluid), RWT (verbal fluency)	Microstate duration, occurrence, coverage; transition probabilities	Eyes-closed; 62-channel; microstate clustering (A–E)	Healthy adults; strict inclusion	n = 168; 20–77 yrs; 58 women (LEMON dataset)	[118]
Age: linear decline, steeper for category than letter. Education ↑ performance (no moderation of decline); no sex effects. Theta ↓ with age; theta ↔ category fluency (positive), not letter; theta did not mediate age–fluency link.	Letter fluency (F-A-S); Category fluency (animals)	Theta power (4–7.5 Hz)	2 min EO; 32-channel; frontal and temporal ROIs	International, healthy; education varied	n = 471; 21–82 yrs; ~51% women	[119]
Higher parietal alpha-1 power at rest (esp. EO) → better WCST (↑NCA, ↓perseveration). Resting alpha may set strategic baseline for EF.	WCST (Heaton); Number of Categories Achieved (NCA), % Perseverative Errors (PPE)	Alpha power and hemispheric asymmetry	Eyes-closed and eyes-open rest; also visuo-motor baseline; 6 sites (bilateral F/T/P); alpha-1 (8.6–10.2 Hz), alpha-2 (10.9–12.5 Hz)	Healthy undergrads (Univ. of Ankara)	n = 16; M = 8/F = 8; M_age = 20.2 (SD 1.4)	[120]
Greater resting network variability (frontal/right central/right parietal) and higher efficiency metrics → higher WM accuracy; cross-validated prediction r = 0.753, RMSE = 0.029. High-accuracy group: stronger/more flexible long-range links.	Visual retro-cue WM (480 trials); accuracy and RT across loads	Temporal variability (fuzzy entropy); network metrics: characteristic path length, clustering, global/local efficiency	5 min eyes-closed; 64-ch; alpha 8–13 Hz; dynamic networks from overlapping epochs via coherence	Healthy young adults	n = 25; 15 M/10 F; 20–27 (M = 23.36)	[121]
Alpha-band frontal–occipital connectivity/efficiency ↑ → higher acceptance rates (more “rational” choices). Predictive model r = 0.58 (*p* = 0.01), RMSE = 10.24%.	Ultimatum Game (responder); acceptance rate	Functional connectivity; graph metrics: clustering (C), global/local efficiency (Ge/Le), path length (L)	5 min eyes-closed; 64-ch; bands δ–γ	Right-handed young adults (UESTC)	n = 16 (from 18); 11 M; 21–25 (M = 23.45)	[122]
Higher resting α and γ mean PLI → longer RTs. In γ: RT ↑ with clustering and path length; RT ↓ with small-worldness → globally efficient networks predict faster responses.	Go/No-Go; RT (M ≈ 307 ms), accuracy (≈98%)	Mean PLI; graph metrics (clustering, path length, small-worldness)	5 min eyes-closed; 64-ch HydroCel; five bands; Phase Lag Index (PLI); 5 clean epochs	Healthy college students	n = 11 final (of 12); 4 F/8 M; 19–21	[123]
High-noise group (lower exponent) up-regulates exponent in No-Go > Go (adaptive noise reduction); low-noise group shows stable pattern. Behaviorally, incongruency slows RT; ↑false alarms in high-noise group only.	Conflict Go/No-Go (audio-visual congruent/incongruent); RT, hits, false alarms	Aperiodic exponent (“neural noise”) at rest and task	2 min fixation; aperiodic exponent from resting spectrum	Healthy young adults	n = 65; 33 F; M_age ≈ 25.7; M_IQ ≈ 109	[124]
Vulnerable group: lower IFS; higher δ/θ/β connectivity (no power diffs). Across all: δ/θ connectivity ↑ ↔ IFS ↓. Schooling ↔ IFS ↑ and δ/θ connectivity ↓. Δ-connectivity + fewer schooling years classify vulnerability (86% accuracy; 82% sens., 89% spec.).	INECO Frontal Screening (IFS)	Spectral power; phase synchrony connectivity	≥10 min eyes-open fixation; high-density EEG; bands δ, θ, α, β	Community adults (Chile); groups matched on age/sex	n = 76; 38 socially vulnerable vs. 38 controls; 34–47; education: 13.8 vs. 18.2 yrs	[125]
No reliable correlations between resting spectral power and behavioral performance → baseline oscillations did not predict Go/No-Go efficiency.	Auditory Go/No-Go; accuracy, RT, RT variability; ERPs also recorded	Resting spectral power by band	Rest EC and EO; bands: δ (1–3), θ (4–7), α-1 (8–10), α-2 (11–13), β-1 (14–20), β-2 (21–29)	Healthy university students	n = 40; 16 M; 18–27 (M = 20.3, SD = 2.3); right-handed	[126]
Higher rest δ-1 (EC) → shorter RTs. Higher rest α-3 → larger P3b. RTV ↑ with P2 amplitude; mean RT ↓ with P3b amplitude. EC→EO reactivity not predictive.	Auditory Go/No-Go; RT, RTV, errors; ERPs (P2, P3b)	Rest components (e.g., δ-1, α-3); task ERPs via t-PCA (P2, P3b)	Rest EC and EO; f-PCA of resting spectra; components incl. δ-1, α-3	Healthy students	n = 20; 8 M; 18–30; right-handed	[127]
Strong Bayesian support for null: resting rsEEG spectral measures (power/ratios/asymmetry) not reliably associated with EF indices (despite high reliability and broad variance).	Brief EF battery: Choice RT (WM), Switching (flexibility), Anti-saccade (inhibition), Mental Rotation	Relative power; band ratios (θ/α, β/α, etc.); hemispheric asymmetry; coherence	Rest EO and EC; 19 sites; θ (4–7), α (7–13; low/upper), β (12–24); ratios, asymmetry, coherence	Healthy young adults	n = 162 usable (of 165); M_age ≈ 22.5	[128]
Higher exponent → better baseline cognition (esp. EF), not change. Lower IAPF → greater 10-yr cognitive decline (esp. EF). IAPF × exponent interaction: “matched” pairs predict stability; “mismatched” predict greatest decline.	BTACT: EF and episodic memory composites; baseline and change	Individual Alpha Peak Frequency (IAPF); aperiodic exponent	128-ch; rest EO and EC; frontal/central focus	Community adults; longitudinal	n = 235; 36–83 (M ≈ 55); 60% F; diverse education; ~10-yr follow-up	[129]
FTMT correlates with TMT and RFFT; TMT shows no beta link. FTMT Part D time negatively correlated with right frontal high-beta (F8) → stronger right-frontal beta ↔ faster FTMT; supports FTMT sensitivity to right frontal EF.	FTMT (new), TMT (A/B), Ruff Figural Fluency (RFFT)	Low/high beta magnitude (right vs. left frontal)	QEEG focusing on frontal beta at F7/F8 (resting)	Healthy undergrads	n = 42; all male; 18–29 (M ≈ 20); right-handed	[130]
Left-lateralized β/α in mMFG → smaller switching costs (better phasic control). Right-lateralized β/α in mMFG → smaller mixing costs (better sustained control). Right-lateralized orbital gyri and mid-posterior SFG/pre-SMA → better sustained control.	Three task-switching paradigms (verbal, spatial, color–shape); switching costs (phasic) and mixing costs (sustained)	Prefrontal β/α lateralization (mMFG); also orbital gyri and mid-posterior SFG/pre-SMA	Resting-state EEG focused on prefrontal cortex; spectral power; lateralization analysis (baseline before tasks)	Healthy university students; question: whether prefrontal asymmetry at rest explains phasic vs. sustained control across domains	n = 56; M age ≈ 22.9; 41 F	[131]
Higher θ/β (esp. Fz) → poorer reversal learning (maladaptive risk adjustment). Ratio predicted performance beyond theta or beta alone. Punishment sensitivity modestly ↓ correlated with θ/β; BIS/BAS did not predict learning.	Computerized gambling reversal-learning task with three 80/20 contingency phases	Theta/beta (θ/β) ratio, esp. at Fz (also checked central/parietal)	4 min total (≈2 min eyes-open/2 min eyes-closed); 32 electrodes; frontal midline focus (e.g., Fz)	Naïve to task; BIS/BAS prior to EEG	n = 128; M age ≈ 22; 87 F; mostly right-handed	[132]
Higher θ/β and δ/β → more disadvantageous/risky choices overall. Frontal θ/β and δ/β and parietal δ/β positively related to disadvantageous choices.	Iowa Gambling Task (100 draws; 5 × 20 blocks); median-split + correlations	δ/β and θ/β ratios	4 min resting EEG; 10/20 montage (frontal, central, parietal, occipital); bands: δ, θ, β; computed ratios at frontal and parietal sites	Healthy students	n = 28; all female; M age = 20; right-handed	[133]
Higher resting θ/β → poorer IGT learning. Effect driven by higher theta power; beta not significantly related.	Iowa Gambling Task (learning to avoid disadvantageous decks)	θ/β ratio; also examined theta and beta power separately	4 min (2 min eyes-open + 2 min eyes-closed); 9 electrodes (10/20); artifact-cleaned; frontal/central focus (Fz, Cz)	Healthy students	n = 31; 8 M; M age = 23.2	[134]
Higher SW/FW (θ/β) → reduced fearful modulation of inhibition; negatively correlated with ACS (lower self-reported attentional control); positively with approach motivation; negatively with anxiety.	Emotional Go/No-Go (happy vs. fearful faces); trait scales (ACS, STAI-T, BIS/BAS)	Slow-wave/fast-wave (SW/FW), esp. θ/β	Frontal electrodes; short alternating eyes-open/eyes-closed blocks; spectral power density; slow-wave/fast-wave ratios	Trait questionnaires: STAI-T, BIS/BAS, ACS	n = 28; right-handed young women; 19–28 (M = 22.7)	[135]
Frontal θ/β negatively correlated with ACS (r = −0.33). θ/β moderated stress impact on attentional control: higher ratio → larger post-stress decline (~28% variance explained). No moderation for state anxiety changes.	Stress manipulation (camera intro + evaluative timed arithmetic) vs. easy control; state attentional control (VAS) pre/post; ACS	θ/β ratio (frontal focus)	Resting frontal EEG; θ/β ratio; site comparison (frontal > central > parietal)	Randomized to stress (CPA-like) vs. control; trait STAI-T and ACS; VAS pre/post	n = 77 analyzed (30 M/47 F) from N = 80; M age = 19.6	[136]

**Table 2:** Risk of bias assessment of included studies.

Bias in Selection of the Reported Result	Bias in Measurement of Outcomes	Bias Due to Missing Data	Bias Due to Deviations from Intended Interventions	Bias in Classification of Interventions/Exposures	Bias in Selection of Participants	Bias Due to Confounding	Study
Serious risk	Low to moderate risk	Low risk	Low risk	Low risk	Moderate risk	Moderate to serious risk	[74]
Moderate risk	Low to moderate risk	Low risk	Low risk	Low risk	Moderate risk	Moderate risk	[75]
Moderate to serious risk	Moderate risk	Low risk	Low risk	Low risk	Moderate risk	Serious risk	[76]
Moderate to serious risk	Moderate risk	Low risk	Low risk	Low risk	Serious risk	Moderate risk	[77]
Moderate risk	Low risk	Low to moderate risk	Low risk	Low risk	Moderate risk	Moderate risk	[78]
Moderate risk	Low risk	Low risk	Low risk	Low risk	Low to moderate risk	Moderate risk	[79]
Moderate risk	Low risk	Moderate risk	Low risk	Low risk	Moderate risk	Moderate to serious risk	[80]
Moderate risk	Low risk	Low risk	Low risk	Low risk	Low risk	Low to moderate risk	[81]
Moderate risk	Low risk	Low risk	Low risk	Moderate risk	Low risk	Moderate to serious risk	[82]
Moderate risk	Low risk	Low risk	Not applicable	Low risk	Low risk	Low to moderate risk	[83]
Moderate risk	Low to moderate risk	Low risk	Low risk	Low risk	Low to moderate risk	Moderate risk	[84]
Moderate risk	Low risk	Moderate risk	Moderate risk	Low risk	Low to moderate risk	Moderate to serious risk	[85]
Moderate risk	Low to moderate risk	Low risk	Low risk	Low risk	Low risk	Moderate risk	[86]
Moderate risk	Moderate risk	Low risk	Low risk	Low to moderate risk	Moderate risk	Moderate to serious risk	[87]
Low to moderate risk	Low to moderate risk	Low risk	Low risk	Low risk	Low to moderate risk	Moderate to serious risk	[88]
Moderate risk	Low risk	Low to moderate risk	Low risk	Low risk	Low to moderate risk	Moderate risk	[89]
Moderate risk	Moderate risk	Low to moderate risk	Low risk	Moderate to serious risk	Moderate risk	Serious risk	[90]
Moderate risk	Low to moderate risk	Moderate risk	Low risk	Low risk	Moderate risk	Moderate to serious risk	[91]
Low to moderate risk	Moderate risk	Low risk	Low risk	Moderate to serious risk	Low to moderate risk	Moderate risk	[92]
Moderate risk	Low risk	Low risk	Low risk	Low risk	Low to moderate risk	Moderate risk	[93]
Moderate risk	Low risk	Low risk	Low risk	Low risk	Low to moderate risk	Moderate risk	[94]
Moderate risk	Low risk	Low risk	Low risk	Low risk	Moderate risk	Moderate to serious risk	[95]
Low to moderate risk	Moderate risk	Low risk	Low risk	Low risk	Low risk	Low to moderate risk	[96]
Moderate risk	Moderate risk	Low risk	Low risk	Low risk	Moderate to serious risk	Moderate risk	[97]
Moderate to serious risk	Moderate risk	Serious risk	Low risk	Low to moderate risk	Serious risk	Serious risk	[98]
Moderate risk	Moderate risk	Serious risk	Low risk	Low risk	Moderate risk	Moderate to serious risk	[99]
Moderate risk	Low to moderate risk	Low risk	Low risk	Low risk	Moderate risk	Moderate to serious risk	[100]
Moderate risk	Low risk	Low risk	Low risk	Low risk	Low to moderate risk	Moderate risk	[101]
Low risk	Low risk	Low risk	Low risk	Low risk	Low risk	Low to moderate risk	[102]
Moderate risk	Moderate risk	Low risk	Low risk	Low risk	Low to moderate risk	Moderate risk	[103]
Moderate risk	Low risk	Moderate risk	Low risk	Low risk	Low risk	Moderate risk	[104]
Moderate risk	Low risk	Low risk	Low risk	Low risk	Low risk	Low to moderate risk	[105]
Moderate risk	Low risk	Moderate risk	Low risk	Low risk	Low risk	Low to moderate risk	[106]
Moderate to serious risk	Moderate risk	Low risk	Low risk	Low risk	Moderate risk	Moderate to serious risk	[107]
Moderate risk	Low risk	Low risk	Low risk	Low risk	Low risk	Low to moderate risk	[108]
Moderate risk	Low risk	Low to moderate risk	Low risk	Low risk	Low to moderate risk	Moderate to serious risk	[109]
Moderate risk	Low risk	Moderate risk	Low risk	Low risk	Moderate risk	Moderate risk	[110]
Moderate risk	Low risk	Low to moderate risk	Low risk	Low risk	Low risk	Low to moderate risk	[111]
Low to moderate risk	Moderate risk	Moderate risk	Low risk	Moderate risk	Low risk	Moderate to serious risk	[112]
Low to moderate risk	Low risk	Low risk	Low risk	Low risk	Low to moderate risk	Moderate risk	[113]
Serious risk	Moderate to serious risk	Moderate risk	Low risk	Low risk	Low to moderate risk	Moderate risk	[114]
Moderate risk	Low risk	Low risk	Low risk	Low risk	Low to moderate risk	Low risk	[115]
Moderate risk	Low risk	Low risk	Low risk	Low risk	Low risk	Low to moderate risk	[116]
Low risk	Low to moderate risk	Low risk	Low risk	Low risk	Low risk	Low risk	[117]
Moderate risk	Low risk	Moderate risk	Low risk	Low risk	Low risk	Moderate to serious risk	[118]
Moderate risk	Low risk	Low risk	Low risk	Low risk	Low to moderate risk	Moderate risk	[119]
Moderate risk	Low to moderate risk	Low risk	Low risk	Low risk	Low risk	Low to moderate risk	[120]
Moderate risk	Low to moderate risk	Low risk	Low risk	Low risk	Low risk	Low to moderate risk	[121]
Moderate risk	Low risk	Low risk	Low risk	Low risk	Low to moderate risk	Moderate risk	[122]
Moderate risk	Low risk	Low to moderate risk	Low risk	Low risk	Moderate risk	Moderate to serious risk	[123]
Moderate risk	Low risk	Low risk	Low risk	Low to moderate risk	Low risk	Moderate to serious risk	[124]
Moderate to serious risk	Moderate risk	Low risk	Low risk	Low risk	Moderate risk	Moderate to serious risk	[125]
Moderate risk	Low to moderate risk	Low risk	Low risk	Low risk	Low risk	Low to moderate risk	[126]
Moderate risk	Low risk	Low risk	Low risk	Low risk	Low risk	Low to moderate risk	[127]
Low to moderate risk	Low risk	Low risk	Low risk	Low risk	Moderate risk	Low to moderate risk	[128]
Low risk	Low risk	Moderate risk	Low risk	Low risk	Low to moderate risk	Moderate risk	[129]
Moderate risk	Moderate to serious risk	Low risk	Low risk	Low risk	Moderate risk	Serious risk	[130]
Moderate risk	Low risk	Low risk	Low risk	Low risk	Low risk	Low to moderate risk	[131]
Moderate risk	Low to moderate risk	Low risk	Low risk	Low risk	Low risk	Low to moderate risk	[132]
Serious risk	Moderate risk	Low risk	Low risk	Moderate risk	Low to moderate risk	Serious risk	[133]
Moderate risk	Low risk	Low risk	Low risk	Low risk	Low to moderate risk	Moderate risk	[134]
Moderate to serious risk	Moderate risk	Low risk	Low risk	Low risk	Low to moderate risk	Moderate to serious risk	[135]
Low to moderate risk	Moderate risk	Low risk	Moderate risk	Low risk	Low risk	Moderate risk	[136]

## Data Availability

No new data were created or analyzed in this study. Data sharing is not applicable to this article.

## Supplementary Materials

The following supporting information can be downloaded at: https://www.mdpi.com/article/10.3390/jcm15031306/s1, File S1: PRISMA-ScR Checklist.
